# Are UX Evaluation Methods Providing the Same Big Picture?

**DOI:** 10.3390/s21103480

**Published:** 2021-05-17

**Authors:** Walter Takashi Nakamura, Iftekhar Ahmed, David Redmiles, Edson Oliveira, David Fernandes, Elaine H. T. de Oliveira, Tayana Conte

**Affiliations:** 1 Institute of Computing (IComp), Federal University of Amazonas (UFAM), Avenida Rodrigo Otávio 6200, Manaus 69067-005, Brazil; david@icomp.ufam.edu.br (D.F.); elaine@icomp.ufam.edu.br (E.H.T.d.O.); tayana@icomp.ufam.edu.br (T.C.); 2 Department of Informatics, University of California Irvine (UCI), Irvine, CA 92697, USA; iftekha@uci.edu (I.A.); redmiles@ics.uci.edu (D.R.); 3 Secretaria de Estado da Fazenda do Amazonas–SEFAZ/AM, Avenida Andre Araujo 150, Manaus 69060-000, Brazil; edson.cesar@sefaz.am.gov.br

**Keywords:** user experience, long-term user experience, longitudinal UX evaluation, user experience evaluation methods

## Abstract

The success of a software application is related to users’ willingness to keep using it. In this sense, evaluating User eXperience (UX) became an important part of the software development process. Researchers have been carrying out studies by employing various methods to evaluate the UX of software products. Some studies reported varied and even contradictory results when applying different UX evaluation methods, making it difficult for practitioners to identify which results to rely upon. However, these works did not evaluate the developers’ perspectives and their impacts on the decision process. Moreover, such studies focused on one-shot evaluations, which cannot assess whether the methods provide the same big picture of the experience (i.e., deteriorating, improving, or stable). This paper presents a longitudinal study in which 68 students evaluated the UX of an online judge system by employing AttrakDiff, UEQ, and Sentence Completion methods at three moments along a semester. This study reveals contrasting results between the methods, which affected developers’ decisions and interpretations. With this work, we intend to draw the HCI community’s attention to the contrast between different UX evaluation methods and the impact of their outcomes in the software development process.

## 1. Introduction

User eXperience (UX) is a term that emerged as an umbrella phrase for new ways of understanding and studying the quality-in-use of interactive products [1]. While usability primarily focuses on pragmatic aspects, such as users’ cognition and performance, UX also augments the subjective, addressing not only aspects related to task accomplishment, but also affect, sensations, and emotions [2], thus subsuming usability. With the increased availability of competing software products, it is of utmost importance to create a positive user experience since it can heavily influence adoption and continued use of software applications [3]. Consequently, in recent years, UX has received increased attention from researchers and practitioners [4].

Researchers have been investigating various ways to design software with a positive UX [5], including developing different UX evaluation methods to assess the experience the software conveys and improve its quality based on users’ feedback. In this context, standardized questionnaires based on scales have been among the most employed types of UX evaluation methods [1,4]. It is because they are quick and easy to use, allowing gathering both positive and negative experiences, and users do not need to interact with a moderator to make their evaluation [6]. However, recent research has been pointing out some concerns about their outcomes.

The main limitation is that most of these methods are purely quantitative, failing to address the causes behind the ratings provided through the scales. Users, for instance, cannot fully report their experiences due to the lack of a field to provide a written report of the experience [7,8]. Moreover, studies have been pointing out varied results (sometimes even contradictory) when applying different methods [7,9,10] or evaluating in other moments of the interaction (e.g., retrospectively or concurrently) [11,12]. This issue is critical, as developers can make different decisions according to the results obtained from these methods [10], which, in turn, could affect the UX conveyed by the application and, consequently, users’ acceptance and continuous usage. Finally, prior research into different UX evaluation methods primarily focused on conducting single experiments, which cannot address the nuances of users’ experiences over time [13]. From a practitioner’s point of view, understanding how user experiences with the software product evolves in the long run (i.e., improving, deteriorating, or being stable) is essential to identify how well the product meets user needs, and to trace out strategies to keep users engaged and motivated to use it. In this sense, it is essential to investigate the outcomes of different UX evaluation methods, and how practitioners interpret results when they are different and contradictory.

To address the above challenges and fill gaps in the literature, we set about to evaluate these methods more comprehensively from the developers’ and end users’ perspectives. We carried out a longitudinal study of an educational system with 68 undergraduate students from the Federal University of Amazonas (UFAM) enrolled in four majors. We employed three UX evaluation methods to evaluate an online judge system and compared the results of the methods. We also presented the results to the system developers and conducted interviews to understand the challenges they face when adopting such methods in their workflow.

With this work, we intend to draw the attention of the human–computer interaction (HCI) community to the existing UX evaluation methods and the impact of their outcomes in developers’ decisions during a software development process. We expect that our results will promote further discussions in the HCI community about the methods being employed in the industry, leading to better evaluation methods.

Overall, the work we report on here makes the following contributions:

- We report on a longitudinal analysis of the outcomes from three different UX evaluation methods and interviews with developers, providing empirical evidence on the impact of these outcomes in the development process.
- Based on our results, we outline implications for practitioners and researchers, and provide suggestions to improve the UX evaluation methods to get more comprehensive and consistent results.

The remainder of the paper is as follows: Section 2 presents the related work; Section 3 presents the methodology we followed in conducting the study; Section 4 presents the results; in Section 5, we discuss our main findings and the implications for practitioners and researchers; in Section 6, we present the threats to validity; finally, Section 7 concludes the paper.

## 2. Related Work

Different UX evaluation methods have been proposed in the literature, focusing on varying aspects, such as enjoyment, aesthetics, and affect [14,15,16,17]. Our current work builds upon—and is informed by—prior research on such methods.

Specifically, several studies evaluating UX methods have focused on applying them in short-term experiences, primarily focusing on product qualities during initial use [18,19]. The majority of these studies also apply UX methods in a single session [4]. By contrast, in longitudinal studies, repeated measurements are taken over a more extended period, allowing researchers to identify and observe any changing characteristic [20]. Due to the potential of richer data and more nuanced findings, there is an increasing interest in conducting longitudinal studies [21].

Other studies performing comparative evaluations of different UX methods have begun to indicate variations in results, making it challenging to identify which results to trust and, consequently, affecting developers’ decisions in the development process [7,10]. Marques et al. [7] investigated the adequacy of scale-based methods to identify problems that affect UX evaluation of mobile applications. Their results showed that participants using one of the methods tended to evaluate the UX conveyed by the system positive, while others considered it neutral. Borsci et al. [10] investigated the relationship among three different evaluation methods by analyzing the variation of the outcomes. The results indicated that the average scores from one of the methods were significantly higher than of the other two. The authors state that this variation may lead practitioners to make different decisions about the platform under evaluation. Practitioners relying only on the results from the method that led to higher scores may report to designers that the platform is very satisfactory and no further analysis is needed. Alternatively, practitioners basing their decisions on the other two methods would likely report that the platform is reasonably satisfactory, but further analysis and redesign could improve its UX. Although Borsci et al. [10] discussed the implications for practitioners, they did not validate their assumptions by interviewing the developers of the platform and showing them the results to get their feedback.

In the most similar study to ours, Laugwitz et al. [15] evaluated whether the scales from UEQ are related to the scales from AttrakDiff. They conducted a study with 16 students to evaluate the UX of a customer relationship management system, each of the students employing both methods. The results indicated that dimensions from UEQ that are similar in concept with the dimensions from AttrakDiff were highly correlated. Laugwitz et al. [15] did not investigate whether these methods provided similar results over time and the implications of these differences, if any, on the development process. In contrast to the single study conducted by Laugwitz et al. [15], we conducted a longitudinal study to investigate whether these two methods provide similar results. We also interviewed the developers of the evaluated system to get feedback on the results from practitioners’ perspective.

In sum, existing research highlights the existence of variations among the results from different UX evaluation methods. However, researchers have yet to evaluate this variation from a longitudinal perspective. Moreover, these works did not assess the impact of such variations on future releases from developers’ point of view. To fill these gaps, we conducted a longitudinal study to capture users’ experience over time. We also investigated the influence of these outcomes on the development process from developers’ perspectives by interviewing the evaluated system developers.

## 3. Methodology

In the following subsections, we describe the methodology we followed to collect, process, and analyze the data.

### 3.1. UX Evaluation Methods

Several UX evaluation methods have been proposed in the literature, which can be classified into three major categories [22]: observation, psychophysiological measurements, and self-reporting. In observation, the user interacts with a product while being observed by a moderator who takes notes about the user’s facial, body, and vocal expressions to capture the user’s emotions and reactions. Psychophysiological measurements consist of using sensors to get objective measurements, such as pupil dilatation, heartbeat, and skin conductivity, which can be used, for instance, to detect changes in user’s emotions or behavior [13]. Finally, self-reporting consists of the user evaluating their UX by using methods such as questionnaires, diaries, and participating in interviews.

As our main goal was to assess the variance of UX dimensions over time, we sought methods that would evaluate a variety of UX dimensions and provide measurable outcomes, in addition to being fast and easy to apply. In this study, we focused on standardized questionnaires, which are one of the most versatile and employed types of evaluation method [1,4], being possible to be applied in different periods of experience (e.g., before, during, and after use) without requiring any special equipment or software [23]. Among the questionnaire-based methods, we selected two: user experience questionnaire (UEQ) [15] and AttrakDiff [14]. We chose these methods because they are two of the most well-known and used standardized UX evaluation questionnaires [24], employed in several studies [13,25,26,27,28] and contexts, from the evaluation of educational platforms [7,29,30] to the evaluation of human-robot interaction [31]. Additionally, they were designed based on the same theoretical UX model proposed by Hassenzahl [32] and are comparable [15]. We also employed a third method, Sentence Completion [33], to provide an independent view of the results from the two quantitative methods.

AttrakDiff consists of a 7-point semantic differential scale comprising 28 pairs of opposite adjectives (shown in Figure 4). In this scale, the user marks the point closest to the adjective that better represents his/her experience. This method evaluates four UX dimensions [34]: (i) pragmatic quality (PQ): refers to the manipulation of the environment with efficiency and effectiveness; (ii) hedonic quality identity (HQI): evaluates the extent the product allows users to express themselves and be perceived by relevant others; (iii) hedonic quality stimulation (HQS): related to the excitement that the product provides for the user; and (iv) attractiveness (ATT): relates to the user’s general impression of the product.

UEQ also uses a 7-point semantic differential scale. It comprises 26 pairs of opposite adjectives (shown in Figure 5), and evaluates 6 UX dimensions [15,29]: (i) perspicuity (PERSP): relates to the understandability and ease of use; (ii) Effectiveness (EFF): refers to the capacity of performing tasks with efficiency and effectiveness; (iii) dependability (DEP): evaluates whether the user is in control of the product; (iv) stimulation (STIM): evaluates user’s motivation to further use the product; (v) novelty (NOV): relates to the extent the product grabs the attention from users; and (vi) attractiveness (ATT): relates to users’ general impression of the product.

According to Laugwitz et al. [15], the dimensions evaluated by UEQ highly correlate with those evaluated by AttrakDiff. Thus, it is possible not only to identify which dimensions of UX vary over time, but also to compare if both methods capture the same perspectives of the experience. Table 1 presents the correlated dimensions between UEQ and AttrakDiff [15].

To complement the quantitative results from UEQ and AttrakDiff, we also applied sentence completion [33]. In this method, the respondents are provided with the beginning of sentences and complete them in ways that are meaningful to them [35], allowing us to get their responses according to the information we need. The participants had to complete the following sentences: “What I liked the most in CodeBench is…”, and “The worst thing in CodeBench is…”

### 3.2. Participants and Material

We conducted the study in three rounds during the first semester of 2019, initially with 195 participants from UFAM, all undergraduate students from four different majors (mathematics, physics, materials engineering, and mechanical engineering), enrolled in the Introduction to Programming course. A total of 114 students participated in all three rounds. Among them, we excluded the data from 46 students due to evidence of careless responses (details are given in Section 3.3). In the end, due to course dropout, we analyzed the data from 68 participants.

The students evaluated the UX of an online judge system for teaching and learning programming similar to URI Judge (https://www.urionlinejudge.com.br/, accessed on 22 December 2020), called CodeBench (http://codebench.icomp.ufam.edu.br/, accessed on 22 December 2020). This system provides features to detect code plagiarism, share pedagogical materials, and an extensive database of programming exercises. This system also provides an integrated development environment (IDE) with which students can develop solutions for the activities assigned by the teacher and get instant feedback when submitted. Additionally, the system uses gamification to engage students by integrating a role-playing game (RPG)-like game for programming practices. Students can explore a given scenario in this game, interact with other students and non-player characters (NPCs), carry out missions, and perform programming tasks. When the students accomplish a mission, they are rewarded with items and improvements in their character’s skills.

### 3.3. Procedure

In this section, we present the procedures we followed for carrying out the longitudinal study and the interviews with the stakeholders.

#### 3.3.1. Longitudinal Study

We conducted the study in three rounds in a completely randomized design. The first round was held two weeks after the course began when the students started using the online judge system. The second round was conducted a month later. Finally, the third round was carried out two months later, at the end of the semester.

We randomly divided and assigned the participants into two groups. Each group used only one method (UEQ or AttrakDiff) to minimize participants’ effort and fatigue since they would answer the same questionnaire three times throughout the study. Moreover, as the methods have some similar items, the responses to one method might influence the responses to the other. We applied sentence completion to obtain qualitative data regarding participants’ perceptions at the beginning and the end of the course. The questionnaires were integrated into CodeBench, so every round, the students answered the questionnaires before starting using the system. The system automatically assigned each participant that logged into the system to a different method.

Before the beginning of each round, one of the authors presented details about the study in each class. We carried out the first round in the second half of March 2019, two weeks after the beginning of the course. The aim was to capture students’ initial perceptions about CodeBench. In this round, we introduced the concepts of usability and UX, and their importance in users’ satisfaction and in the design of successful products. We also explained the importance of our study without giving too many details. Additionally, we instructed how to fill out the questionnaires and talked about the importance of being honest about their experience with the system. Finally, we asked the participants to review and sign a consent form, explaining their identities’ confidentiality. The second round was carried out a month later, in the second half of April, after the participants became more familiar with the system. Finally, we carried out the third round at the end of the semester, in the middle of June. We asked the students to perform the last evaluation of their experiences with the system.

#### 3.3.2. Identifying Careless Respondents

Before analyzing the results, we reviewed the data for identifying careless respondents, as they can significantly impact experimental analyses [36]. Several works compared different methods for identifying careless respondents [37,38,39], such as calculating the Mahalanobis distance, psychometric synonyms, average time, and longstring. We selected the longstring method due to its effectiveness and the nature of the UX evaluation methods we employed. This method assumes that “too many consecutive identical responses may indicate a lack of effort” from the participant [38]. According to Curran [37] and DeSimone et al. [38], longstring screening is recommended when using multidimensional questionnaires comprised of a mixture of positively and negatively scored items, as the UEQ and AttrakDiff methods.

**Figure 1:**
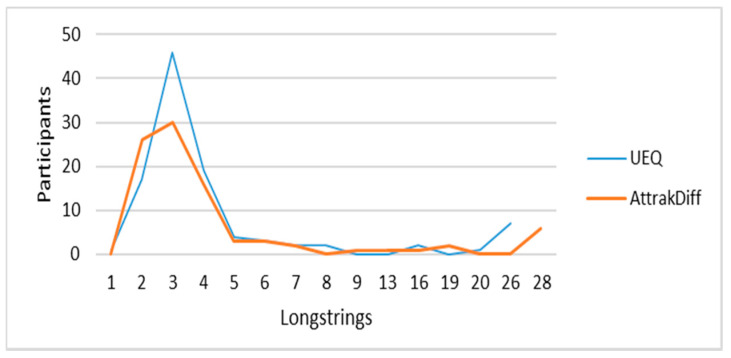
Frequency of longstrings per method.

Following the approach recommended by Huang et al. [39], we analyzed the frequency of longstrings. We defined the cutoff point as 7, as most of the participants had between 1 and 6 longstrings in both groups (see Figure 1). Participants whose longstring were equal to or greater than 50% of the questions were automatically considered careless respondents and removed. We performed an additional analysis for the participants with 7 or more invariant answers but less than 50% of the questions. As both UEQ and AttrakDiff have some reversed pairs of adjectives (positive on the left and negative on the right), we analyzed whether the invariant answers occurred in the reversals. Participants with such invariant answers were excluded. Finally, as this is a longitudinal study, if a participant was identified as a careless respondent in one round, we removed his/her data from the analysis. Table 2 shows the distribution of the participants.

#### 3.3.3. Interviews with the Stakeholders

After conducting the three rounds and analyzing the data, we presented the results to three stakeholders to get their feedback on each method’s results and investigate the impact of these results in design decisions. The three stakeholders were as follows: (i) the developer of the online judge system; (ii) a researcher working with this developer to improve its UX; and (iii) a professor that uses this system in her classes and is interested in improving the system). We interviewed each stakeholder individually in a separate session. We presented our findings in a presentation and audio-recorded the interview for further analysis.

## 4. Results

In the following subsections, we present the results from the UX evaluation methods and the interviews with the stakeholders.

### 4.1. Reliability of the Instruments

We started by assessing the reliability of the instruments using Cronbach’s alpha for each dimension per round. It is a measure that ranges from 0 to 1 and evaluates the internal consistency of scales [40]. Although there is not a general rule for the desired value of alpha, it is common to use the rule-of-thumb of 0.70 for an instrument to have an acceptable level of consistency [40,41].

The results for UEQ (Table 3) showed a good internal consistency for attractiveness, perspicuity, efficiency, stimulation, and novelty dimensions, with Cronbach’s alpha > 0.7. The consistency of dependability, on the other hand, was below the acceptable value.

The results for AttrakDiff (Table 4) showed a good internal consistency for attractiveness (ATT), hedonic quality stimulation (HQS), and hedonic quality identity (HQI). However, the pragmatic quality (PQ) dimension showed a consistency below the acceptable value.

### 4.2. Within UX Dimensions over Time

**Figure 2:**
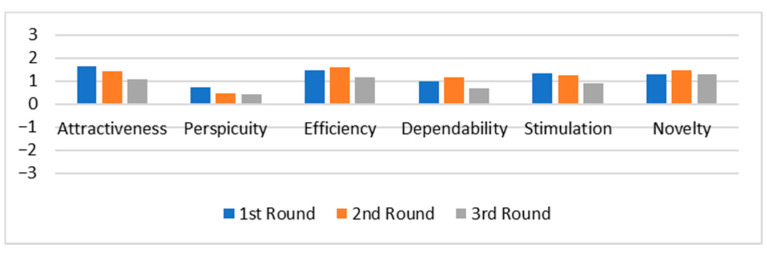
Mean of each UX dimension from UEQ.

**Figure 3:**
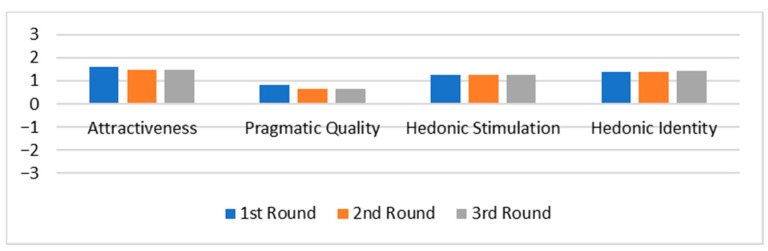
Mean of each UX dimension from AttrakDiff.

To identify which UX dimensions vary over time, we first calculated the mean of each UX dimension per participant in each round. Then, we calculated the overall mean of each UX dimension. Figure 2 and Figure 3 present the results from UEQ and AttrakDiff, respectively. The values of the mean range from −3 to 3. A mean between −1 and 1 represents a neutral perception of the experience, while values above 1 and below −1 represent a positive and negative perception, respectively.

The result from UEQ (Figure 2) indicated that the experience of the participants deteriorated over time in all dimensions, except Novelty. In order to identify whether there was a significant difference between the results from each round, we performed Friedman’s non-parametric statistical test [42]. The results for UEQ indicated a significant difference in four out of six dimensions: attractiveness (x2(2) = 13.888, *p* = 0.001), efficiency (x2(2) = 10.303, *p* = 0.006), dependability (x2(2) = 8.778, *p* = 0.012), and stimulation (x2(2) = 7.672, *p* = 0.022). However, no significant differences were found in perspicuity (*p* = 0.146) and novelty (*p* = 0.834) dimensions.

In order to identify in which rounds the differences actually occurred, we carried out pairwise comparisons. To do so, we ran the post-hoc Wilcoxon signed-rank test. For ATT, the significant difference was from the first to the third round (Z = −3.453, *p* = 0.001, medium effect size r = −0.419), and from the second to the third round (Z = −2.023, *p* = 0.043, small effect size r = −0.245). For EFF, the significant difference was from the first to the third round (Z = −2.162, *p* = 0.031, small effect size r = −0.262), and from the second to the third round (Z = −2.557, *p* = 0.011, medium effect size r = −0.310). For DEP, the significant difference was from the first to the third round (Z = −1.999, *p* = 0.046, small effect size r = −0.242), and from the second to the third round (Z = −2.517, *p* = 0.012, medium effect size r = −0.305). Finally, for STIM, the significant difference was from the first to the third round (Z = −2.628, *p* = 0.009, medium effect size r = 0.319). Overall, this indicates that four out of six UX dimensions from UEQ (66.7%) deteriorated significantly over time since the students started using the system.

The results from AttrakDiff (Figure 3) provided a different view of the experience. All UX dimensions remained stable, except for attractiveness and pragmatic quality, which had a slight decrease. However, we did not find any statistically significant difference in the three rounds using Friedman’s non-parametric statistical test (ATT: *p* = 0.912; PQ: *p* = 0.785; HQS: *p* = 1.000; HQI: *p* = 0.900) and so we did not perform Wilcoxon signed-rank test.

These results reveal that the methods provided an opposing view regarding the UX over time. While UEQ indicated a deteriorating experience, AttrakDiff showed a stable experience. Moreover, although the concept behind the Attractiveness dimension of UEQ is nearly identical to the one from AttrakDiff [15], the results were very different. While the UEQ Group reported a deterioration of the system’s attractiveness over time, the AttrakDiff group reported a stable experience. In the end, the participants who used AttrakDiff perceived their experience with the system as positive, while participants who used UEQ perceived it as neutral.

Observation 1: UEQ and AttrakDiff provide different views regarding UX over time. Despite being nearly identical in concept, the results of the attractiveness dimension from UEQ indicated a different tendency of UX compared to AttrakDiff.

**Figure 4:**
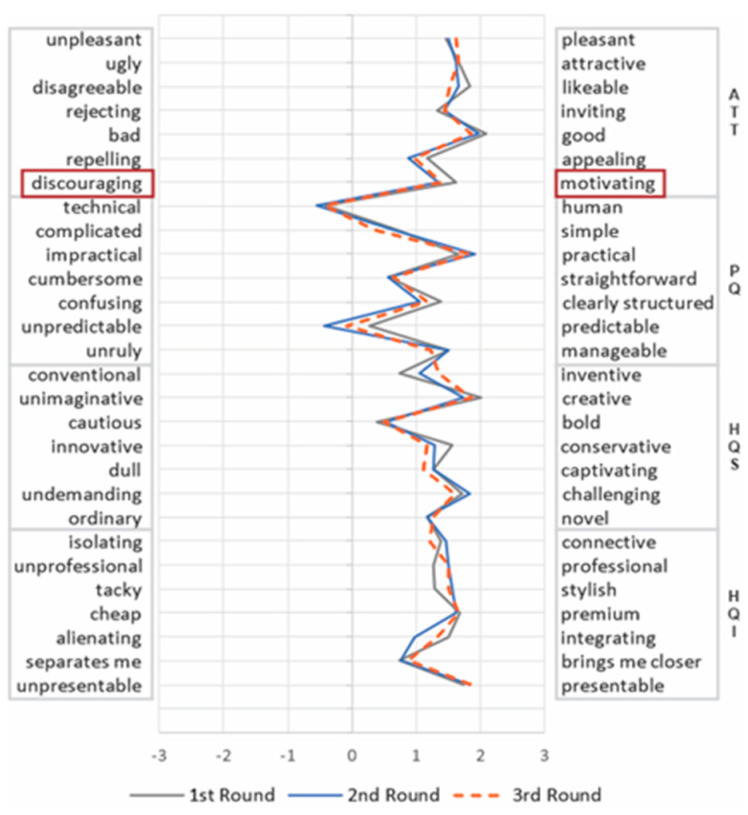
Variation of AttrakDiff evaluated items over time.

**Figure 5:**
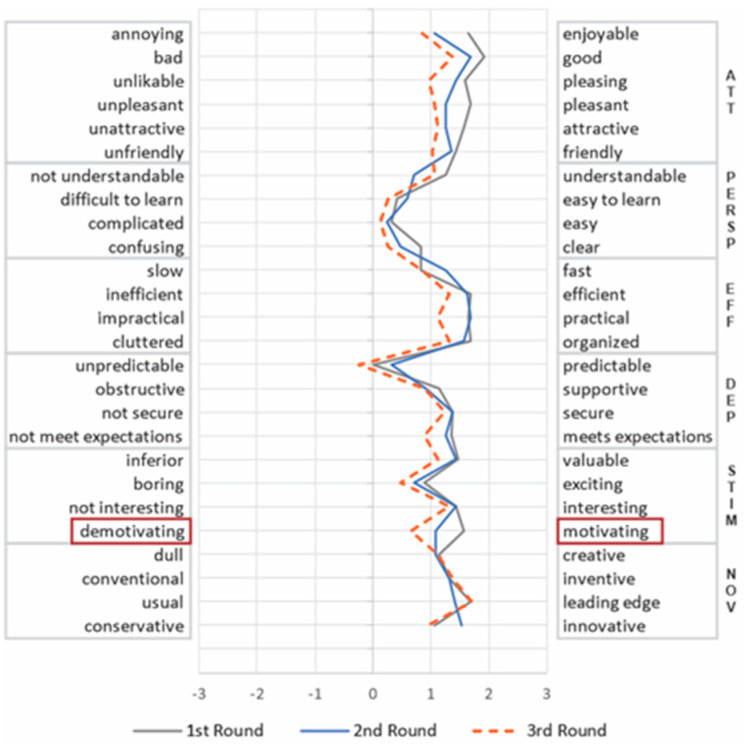
Variation of UEQ evaluated items over time.

We also analyzed the variation of the items related to each UX dimension. This analysis aimed to identify how the perception of the participants on each item changed over time. Figure 4 and Figure 5 present the mean of the items evaluated by UEQ and AttrakDiff, respectively, per round.

The results of UEQ (Figure 5) revealed that the distribution of the answers was consistent in each round, varying less than one point between the items of the same dimension (standard deviation—SD, between 0.18 and 0.35), except for dependability (SD = 0.59). This greater variation is caused mainly by the “Unpredictable/Predictable” adjective pair, which may indicate that these adjectives do not fit very well in the context of an online judge system, or are not well understood by the participants.

For AttrakDiff (Figure 4), the results indicated a relatively consistent distribution of the answers in ATT, HQI, and HQS dimensions in each round, varying less than 1 point between the items of the same dimension (SD between 0.3 and 0.47). On the other hand, the PQ dimension was more diffuse, varying the mean from 1 to 2 points (SD of 0.82). Such result indicates that the items are not consistently measuring variables such as “Technical/Human” and “Unpredictable/Predictable” pairs, which had the lowest scores in the scale and were the only ones negatively evaluated.

Observation 2: the low internal consistency and variations in some dimensions may be related to misinterpretations of some pairs of adjectives or their inadequacy to the context of the evaluation.

### 4.3. Sentence Completion Results

To better understand the results provided by UEQ and AttrakDiff methods, we also applied sentence completion [33]. In this method, the participants had to complete the following sentences: “What I liked the most in CodeBench is…”, and “The worst thing in CodeBench is…”. We then analyzed the participants’ responses using summative content analysis [43] to categorize them according to the concepts identified in each quotation.

#### 4.3.1. UEQ Group

Regarding the worst thing in CodeBench, the most complained issues were the lack of clarity and course-related issues, reported by five participants. Regarding the lack of clarity, the participants said that the worst thing in the system is the confusion and difficulty to understand. However, it is unclear whether these issues were related to the system itself or the course, except for participant P72, who stated: “[the worst thing in CodeBench is] the badly formulated questions.” This quote highlights the difficulty of users in differentiating the content of the course from the system itself. This difficulty becomes clearer when analyzing course-related issues. Participant P24, for example, reported that the “[coding] structures are much complex,” while P40 said that the worst thing is “the assignments.” These reports may explain the low score obtained in perspicuity dimension, which evaluates the ease of use and understandability.

In the third round, the results from sentence completion showed a substantial change. Bugs and performance became more prominent, being reported by six and five participants, respectively. Participant P28, for example, reported that, “during the correction, sometimes it shows that the answer is wrong, even when it is correct.” Participant P75 also stated, “sometimes it has some problems when you submit the code, it says that [the code] is wrong and, soon after, without changing anything and submitting again, it says that [the code] is right.” Regarding performance issues, participants P55 and P61, for example, reported that the system freezes during the exam. Such reports may explain the reduction in the efficiency dimension.

Regarding what they liked the most in the CodeBench system, gamification was the most cited aspect in the first round, with six participants. Innovation and learning support came second with five participants each. The positive perception of their experience regarding attractiveness and stimulation may be associated with gamification, while the positive perception of efficiency may be related to learning support. Participant P47, for example, stated, “[what I like the most is] the gamification and all the story behind the game [that] motivates me, even more, to keep performing the activities.” Participant P72 reported that s/he likes “the speed and efficiency in explaining our mistakes.”

There was also a slight change in the third round. While gamification remained in the first place, with five participants, innovation, on the other hand, was only stated by one participant. In this round, the interface was the second most mentioned aspect, with four participants, while learning support came third, with three participants. Such a result contrasts with the quantitative results, which indicated a slight increase in novelty dimension from the second round. A possibility is that, as participants progress in the game, new features and maps are unlocked, making them realize the innovative nature of the system. On the other hand, the decrease in efficiency may be related to the lower number of participants who commented about the system’s learning support aspects compared to the first round. Finally, the decrease of the attractiveness dimension in the third round may also reflect the reduction in the other dimensions, as it records users’ general impressions [44].

#### 4.3.2. AttrakDiff Group

For the worst thing in CodeBench, most of the complaints were related to bugs, reported by nine participants, followed by the lack of clarity and performance issues, written by three participants. Among the bugs reported, six were related to gamification, one related to errors on the system, and two only mentioned the existence of bugs. Participant P132 stated, “[the worst thing is] the bugs in the RPG game when performing the activities proposed.” For the lack of clarity, participants noted that the system “lacks detailed information”—P138, and is “little explanatory”—P127. Finally, for the performance issues, the participants reported that the system “takes time to load some information”—P121, and that “sometimes it freezes”—P156. The lowest score obtained in the pragmatic quality dimension may be associated with these issues, given that it evaluates the perceived ability to support the effective and efficient achievement of tasks [34].

In the third round, bugs remain the worst thing for the AttrakDiff group, reported by nine participants (five being the same that reported bugs in the first round). However, this time, only one report was related to gamification. Similar to the results from the UEQ group, some participants reported the bug during code correction (four participants), a problem not mentioned in the first round. Participant P111, for example, stated, “[the worst thing is] when I write the code correctly and it says it is wrong.” There were also reports related to the system loop (one participant) and unspecified bugs (three participants). The performance of the system remained as the second most complained issue, reported by three participants. As the complaints related to bugs changed to more specific ones that impair the participants to accomplish their tasks, we would expect at least a slight decrease in the PQ dimension since the first round, as occurred with the UEQ group. However, it remained stable over time.

Regarding what they liked the most in the system, the practicality was the most cited aspect in the first round, with six participants. Other mentioned aspects include interface and programming practice, with four participants, and gamification, reported by three participants. Overall, the participants said they like to program, and the system is practical, with a friendly and easy to use interface. Participant P166, for example, stated, “[what I liked the most in the system is] the easiness to practice programming.” Participant P137, in turn, reported that, “the design is modern, likable, and easy to use.” These positive reports related to pragmatic aspects go against the previous findings on the worst thing in the system, which supported the low pragmatic values.

In the third round, the results had some minor variations. The facility remained one of the main reasons mentioned by five participants, together with the interface, also reported by five participants. However, there was no mention of gamification neither to the practice of programming in this round. These results may be related to the slight drop in hedonic quality stimulation and hedonic quality identity dimensions. The participants may have lost some of their stimuli when using the system and the lack of interest in programming, leading them not to have the same identification with the system as before.

### 4.4. Interviews with the Stakeholders

We divided the interview into four steps. First, we introduced the concept of UX, the goal of the research, and the UX evaluation methods we employed. Second, we presented the graphs with the results from one of the quantitative methods (Figure 2—UEQ or Figure 3—AttrakDiff). We described the concept of each dimension evaluated by the method and their respective pairs of adjectives. Third, together with the graph, we presented some positive and negative quotations obtained from sentence completion. We asked the stakeholder if s/he had any insight or envisioned any change in the system based on these results. We repeated the same procedures from the second and third steps with the other method. Finally, in the fourth step, we aimed to investigate the stakeholder’s perception of the results. We asked whether the graphs provided the same UX perspective and which was best to support identifying or proposing improvements in the system. To avoid primacy bias [45], we alternated the ordering of the results for each participant.

During the interview, the participants mentioned aspects that could be improved, and discussed what they could interpret from the results shared with them. The participants’ quotations helped them to interpret the results better and propose more specific improvements. However, in general, they could derive conclusions and hypotheses about what could have led to the results presented without showing them the quotations.

Developer D1 was excited and satisfied when presented with the AttrakDiff results, as the overall evaluations were positive and remained stable over time. Although pragmatic quality (PQ) was low and the other dimensions decreased a little in the third round, he still perceived the results, in general, as positive. When we presented the results from UEQ, however, he had a different reaction: He stated, “I have a feeling that the feedback from the first graph (AttrakDiff) was more positive than this graph you are presenting (UEQ). This raises some concerns.” In fact, he was more concerned about the system after seeing the results from UEQ, trying to comprehend better the reasons for the decrease in the UX and propose more improvements. It also happened with the other two developers (D2 and D3). They discussed more about the possible reasons for the decrease in the UX and future improvements when seeing the UEQ graph, stating that it is coherent with the quotations and their perceptions about the use of the system. Developer D1, for example, mentioned that they would try to make the system more attractive. They will be adding new gamification elements and bringing the system closer to the students through a more appealing interface that better integrates the IDE with the game. Currently, they are separate things. These results indicate that the methods conveyed different UX perspectives, impacting developers’ interpretation and decision process.

Observation 3: the results conveyed different perspectives of UX to the stakeholders, leading to different reactions and interpretations of the results.

When asked which method better supported them to propose improvements in the system, they were unanimous in pointing out UEQ. We identified that the stakeholders found the UEQ’s dimensions easier to understand. Developer D1, for example, stated, “The second graph [UEQ] was more intuitive. […] I’m not from the UX, software engineering field, I’m a programmer […] it brought a terminology that is much more intuitive for me, as a developer.” Developer D2 also affirmed, “I think that the first [UEQ] is more detailed and auto-explicative […] it uses more normal words.” By contrast, they found AttrakDiff dimensions confusing. Developer D1, for example, stated, “in the first graph [AttrakDiff] was used a nomenclature that I was in doubt about its meaning [referring to the hedonic dimension].” They even had difficulty understanding and remembering the name of the dimensions: “(sic) hendonic, I do not know what is (sic) hendonic, so I needed you to explain to me”—D2.

Another reason for preferring UEQ is its structure, which divides the dimensions into smaller sets and provides detailed information. Developer D2 stated, “[UEQ was better] because it is like it has more categories, so it is not so messed up”. Developer D1 also reported that the greater number of subdimensions helped to identify what aspects of the system should be improved: “As it is more divided, the dimensions became clearer for me to identify where we are doing wrong, where we can improve, where is good, in this sense.” By contrast, the broader dimensions and their confusing nomenclatures and concepts affected their choice for AttrakDiff as stated by developer D3: “[AttrakDiff] is a little confusing because it has the two dimensions related to hedonism. One is Stimulation and the other I forgot. I think that it confuses. It groups more items and you had to explain me [the concept behind] the last one [Hedonic Identity]”.

Observation 4: the lack of clarity can affect the choice of a UX evaluation method. It also makes it difficult to interpret the results and formulate improvements based on them.

## 5. Discussion

### 5.1. Contrasting Results

The results revealed that the methods provided different perspectives of UX over time. While AttrakDiff indicated that students’ experience with the platform remained stable in the long run, UEQ indicated a deteriorating perspective. This finding highlights the importance of carrying out longitudinal studies when comparing different evaluation methods. If we considered only the first round from both methods (see Figure 2 and Figure 3), we would notice the following pattern: (i) attractiveness was the dimension with highest ratings; (ii) hedonic quality placed second; and (iii) pragmatic quality obtained the lowest ratings. From this analysis, we would conclude that both methods are providing similar results. However, the results varied significantly when evaluated longitudinally.

In general, the results from UEQ were consistent with the reports from sentence completion. In the first round, the participants’ main complaints were related to the lack of clarity of the questions and other content-related issues. These results are consistent with the quantitative results, where perspicuity was the dimension with the lowest ratings. When users’ main complaints shifted from the lack of clarity to bugs related to correction problems and performance issues in the third round, it was also reflected in the deterioration of efficiency and attractiveness dimensions. To understand the results better and allow triangulation, we analyzed the system usage from the log files. These log files contain the timestamp of each user interaction when programming through the IDE, such as mouse clicks, element focus, keypress, etc. First, we estimated each student’s average daily system usage by summing the timestamps and dividing by the number of days the student used the system in each round. Then, we calculated the average of each group per round. The analysis revealed an increase in system usage from the first (UEQ: 29 min:23 sec/AttrakDiff: 34:20) to the second round (UEQ: 43:53/AttrakDiff: 49:53), and a slight variation in the third round (UEQ: 44:08/AttrakDiff: 49:20). As the participants used the system more frequently and the programming became more complex, issues that initially did not occur or were not perceptible became more apparent, explaining the deterioration of these dimensions. It is also possible that the participants did not clearly distinguish between the difficulty they were having with the course and the system itself, for example, their difficulty in identifying and solving errors in their codes. Participants from both groups reported this issue. Participant P85 (UEQ) stated: “[the worst thing is] when the programming does not print, you do not know where the error is,” while participant P156 (AttrakDiff) stated: “[the worst thing is] the lack of clarity on where the errors are located.” This result highlights the challenge in evaluating the UX of educational platforms. Students might make their evaluations based on aspects that are not related to the platform itself, such as their difficulty with the subject.

The results from AttrakDiff, on the other hand, were quite different. While in the UEQ group, the UX deteriorated over time in all dimensions, except for novelty, the AttrakDiff group remained relatively stable. In general, it may be explained by the different focus of each method. While AttrakDiff lays more emphasis on hedonic-related dimensions (attractiveness, hedonic quality identity, and hedonic quality stimulation) than on pragmatic ones (pragmatic quality), UEQ has a more balanced structure, addressing both dimensions equally (pragmatic dimensions: dependability, perspicuity, efficiency; hedonic dimensions: attractiveness, novelty, stimulation).

It is noteworthy that this contrasting result also occurred in the Attractiveness dimension, even the definition being nearly identical in both methods. While the Attractiveness from UEQ group deteriorated in every round, the same dimension for the AttrakDiff group remained stable over time. To better understand the contrasting results, we analyzed the items from each method in the light of Hassenzahl’s theoretical model [32,46], which both are based on. According to this model, Attractiveness is the consequence of both pragmatic and hedonic dimensions. It is related to emotional reactions, such as good, sympathetic, pleasant, attractive, motivating, desirable, and inviting [46]. As there is no definition for these emotional reactions in these publications [32,46], we mapped each pair of adjectives with these emotional reactions according to the definitions and synonyms from the Oxford dictionary) (https://www.oxfordlearnersdictionaries.com/, accessed on 22 January 2020) (see Table 5). While the AttrakDiff attractiveness dimension covers five emotional reactions (pleasant, attractive, inviting, good, and motivating), UEQ, in turn, focuses on three (pleasant, good, and attractive). Moreover, motivation in UEQ is not part of the attractiveness dimension as in AttrakDiff, but of stimulation (see the highlighted items in Figure 4 and Figure 5). This different interpretation of what aspects each UX dimension should evaluate may be related to the lack of consensus about the definition of UX [47,48]. These diverse perspectives of UX also implied different behavior from the stakeholders. While a stable perspective kept the stakeholders satisfied, a deteriorating view raised more concerns, leading them to seek to identify the causes for the decrease of the UX over time and propose more improvements.

### 5.2. UX Dimensions

While there were reports from Sentence Completion that support the low scores in the Pragmatic dimension of AttrakDiff during the first round, there were also many positive reports related to other pragmatic aspects, producing conflicting results. In fact, the first round was the one that had the highest variation between the items from this dimension in AttrakDiff. At the same time that the system was perceived as practical (M = 1.6) and clearly structured (M = 1.4), it was also perceived as not so straightforward (M = 0.6), nor so predictable (M = 0.3). It may indicate that the Pragmatic dimension evaluates different aspects of the system. Such a phenomenon also occurred in UEQ. The system was perceived as practical (M = 1.6) and organized (M = 1.7) in the first round, at the same time that it was perceived as not so easy (M = 0.3), nor so predictable (M = 0.0). However, UEQ groups these items into different dimensions (perspicuity, dependability, and efficiency), making it possible to detect these nuances in the pragmatic aspects and avoid higher variations within the dimensions. The interviews with the stakeholders corroborated this finding. All agreed that the dimensions from UEQ were clearer, and the outcomes provided more detailed information about the UX conveyed by the system.

The interviews also revealed that the developers had difficulty understanding the concept behind some dimensions from AttrakDiff. Consequently, practitioners may have difficulty knowing what is being evaluated and interpret the results almost instinctively [47], leading to wrong decisions due to misinterpretations. This issue is critical, as practitioners consider UX evaluation metrics essential to justify decisions, validate design goals, and convey a sense of reliability [49].

### 5.3. Reliability of the Instruments

Both methods had at least one dimension below the typical acceptable level of consistency: Dependability in UEQ, and pragmatic quality in AttrakDiff. For UEQ, lower internal consistency values in the dependability dimension were previously reported by [29] when evaluating a Learning Management System (LMS). The authors stated that this could be because this property does not play an essential role for the UX in this type of system or due to problems with interpreting the items in the scale. Although CodeBench system is not an LMS, it is also designed for educational purposes, explaining the similarity of the results. Moreover, our findings revealed that this inconsistency might be related to the “Predictable/Unpredictable” pair, as the alpha of the dependability dimension increases when removing it from the scale. It indicates a possible divergence in the interpretation of this pair by the participants. Regarding AttrakDiff, we also found that removing “Human–Technical” and “Predictable–Unpredictable” pairs increases the internal consistency of the Pragmatic Quality dimension. Moreover, the “Predictable/Unpredictable” pair is the same pair whose removal increases the consistency of the dependability dimension from UEQ. Such finding reinforces the possibility of misinterpretation of these adjectives or their inadequacy to evaluate this product type.

### 5.4. Implications

Below we list some implications for both practitioners and researchers.

#### 5.4.1. Implications for Practitioners

The results highlighted the importance of longitudinal evaluations for the stakeholders to understand how well the software product performs, as a single assessment cannot provide an evolving perspective of the experience. Practitioners should perform long-term evaluations to understand better the UX conveyed by the software product and plan future releases accordingly.

In this context, interpreting the result is essential to identify possible improvements. From users’ point of view, the lack of consistency or the possibility of misinterpreting the methods’ items may lead to biased results, as they may not know what is being evaluated and how to evaluate it. From practitioners’ point of view, if it is not clear what a method is evaluating, they may find it challenging to interpret the results and make incorrect decisions. We found that using terminology that is familiar to the practitioner facilitates the interpretation of the results. Thus, before selecting a method, practitioners should verify which UX dimensions they evaluate and whether they apply for the evaluated product.

Finally, the outcomes of the methods we evaluated raise the question of which result to rely upon, as each provided a different view of the experience over time (deteriorating vs. stable), even when based on the same theoretical model. This issue is critical, as developers can make different design decisions based on the results. While the stable perspective kept the stakeholders satisfied, the deteriorating view raised some concerns that instigated them to investigate the causes of UX’s decrease. The triangulation with the qualitative data indicated that UEQ could capture the nuances of the variation of UX better than AttrakDiff, leading to more consistent results due to the fine-grained dimensions. The stakeholders also perceived the terminology used to identify each dimension in UEQ as easier to understand what is being evaluated. Although scale-based evaluation methods are a common way to quickly and easily assess the UX [1,4,6], there is a need to obtain qualitative data to interpret the results better and support the decision-making process. Practitioners should, whenever possible, combine quantitative and qualitative methods to get more data and identify the causes behind negative and positive evaluations.

#### 5.4.2. Implications for Researchers

The results highlighted the importance of conducting longitudinal studies when comparing the results from different UX evaluation methods. In the first round, the results were quite similar but evolved differently over time, providing contrasting perspectives of UX. We encourage researchers to conduct longitudinal studies when comparing UX evaluation methods and better understand how UX evolves over time. Our study, along with others, shows that ambiguous or inadequate items may lead to inaccurate results. Researchers may develop a method that is not being well understood and, consequently, leads to biased results. Studies can be conducted to investigate users’ comprehension of the items evaluated by different methods and identify the aspects of the product/interaction they consider when evaluating them. By doing so, researchers can investigate whether these items are adequate for evaluating a given type of product or whether they are being understood according to their definition and purpose.

The lack of a shared definition of UX is critical, leading to an endless number of dimensions evaluating the same phenomenon [1]. Due to this, there is no consensus about which aspects each dimension should measure, which can affect the development of new methods. As we pointed out in Section 5.1, “motivation” is part of the Attractiveness dimension in AttrakDiff, while in UEQ it is part of the Stimulation dimension. As new methods are usually proposed based on consolidated methods to evaluate new types of products and contexts, such as IoT environments [50], it is important that we, as a community, be aware of these implications and devise solutions before they go out of hands. Researchers can, for example, employ machine learning techniques to analyze massive data sources, such as user reviews from app stores (like Play Store and App Store). By doing so, they can obtain the top words from each app category and identify which UX dimension they are related to, making it possible, for instance, to get insights about what dimensions should be considered when evaluating a given type of software product.

The way that UX dimensions are structured may influence the level of details that the method can capture. Dividing broad dimensions into smaller ones seems to capture the nuances of UX over time in more detail, providing more information to practitioners. Researchers should consider using more fine-grained dimensions instead of broader ones.

Finally, dimensions with more comprehensive definitions and nomenclature may help practitioners interpret the results better. This issue is critical, as it can influence the adoption of the method by practitioners from the industry. According to the survey results presented by Law et al. [49], the lack of training for defining meaningful metrics and analyzing and interpreting quantitative results might discourage practitioners from employing this type of method. Practitioners may be hesitant to adopt a method that is confusing and is unclear about what it measures. In this sense, researchers should avoid using nomenclatures and definitions that are not so trivial for practitioners when developing UX evaluation methods.

## 6. Threats to Validity

The main limitation of this study is the generalizability of the results since we conducted it in a specific system. Further research is needed to evaluate whether the same results can be obtained in other courses and/or systems.

Another limitation can arise due to the self-selection bias of the participants as we only analyzed the data from consenting participants. However, we used randomized sampling and group assignment to reduce the effect of the bias on our findings.

Finally, the comparison of two scale-based methods might limit the generalization of the results. However, given that scale-based questionnaires are the most used method to evaluate different UX dimensions [4], our results may provide insightful contributions for researchers and practitioners.

## 7. Conclusions and Future Work

Previously, several surveys with practitioners from the industry have been conducted to investigate their understanding of UX’s concept and its importance in the development process [2,47,49,51]. Along with other results, these surveys highlighted that quantitative data could be essential to support and justify decisions. Thus, it is important to investigate whether different quantitative methods can provide consistent information that helps developers justify their decisions in the development process.

This paper aimed to investigate whether two similar quantitative evaluation methods provide the same UX perspective over time and how the results affect developers’ comprehension and decisions for improving a product. Our results revealed that the outcomes could vary considerably according to the method employed, leading to different design decisions. A stable perspective of UX made developers satisfied with the results, leading them into a more comfortable zone. By contrast, a deteriorating view raised concerns about what is affecting their experience. As a result, practitioners may postpone product improvements, thinking that the experience the system is conveying is satisfactory when it is not, leading users to look for alternatives that meet their needs. In this sense, practitioners from the industry should be aware of possible variations between different UX evaluation methods. Employing multiple methods in a qualitative–quantitative approach may provide better insights and avoid misinterpretations of the results.

For researchers, our findings highlight that there is still a long road ahead. It is important that the HCI community focus on better defining and scoping the concept of UX and its role in the development process. Ideally, it would be possible to develop more comprehensive and consistent UX evaluation methods, allowing practitioners to interpret the results better. Researchers might also focus on methods that can be adapted according to the context, type of product, or evaluation focus, which may help practitioners getting more precise results. Another possibility is investigating the conceptual differences behind the dimensions evaluated by the existing methods to develop a guide that helps practitioners select the most appropriate ones according to their needs. Doing so would reduce the problems with misinterpretations, allowing practitioners to focus on important aspects to be evaluated according to the specificities of the product they are developing.

## Figures and Tables

**Table 1:** Correlated dimensions between UEQ and AttrakDiff.

UEQ	AttrakDiff
Attractiveness	Attractiveness
Perspicuity, Efficiency, Dependability	Pragmatic Quality
Stimulation, Novelty	Hedonic Quality Stimulation
	Hedonic Quality Identity

**Table 2:** Distribution of the participants along the study.

**UEQ Group**					
	**Mathematics**	**Mechanical Engineering**	**Materials Engineering**	**Physics**	**Total**
Initial	24	29	25	26	104
Longitudinal	14	17	20	7	58
Careless	6	7	9	2	24
Analyzed	8	10	11	5	34
**AttrakDiff Group**					
	**Mathematics**	**Mechanical Engineering**	**Materials Engineering**	**Physics**	**Total**
Initial	23	20	20	28	91
Longitudinal	14	11	19	12	56
Careless	6	3	5	8	22
Analyzed	8	8	14	4	34

**Table 3:** Cronbach’s alpha coefficient for UEQ.

	ATT	PERSP	EFF	DEP	STIM	NOV
1st	0.899	0.830	0.795	0.464	0.877	0.737
2nd	0.923	0.843	0.864	0.743	0.792	0.742
3rd	0.847	0.873	0.824	0.653	0.810	0.663
Avg.	0.890	0.849	0.828	0.620	0.826	0.714

**Table 4:** Cronbach’s alpha coefficient for AttrakDiff.

	ATT	PQ	HQS	HQI
1st	0.777	0.506	0.629	0.716
2nd	0.934	0.653	0.753	0.785
3rd	0.949	0.771	0.796	0.874
Avg.	0.887	0.643	0.726	0.792

**Table 5:** Cronbach’s alpha coefficient for AttrakDiff.

UEQ		AttrakDiff	
Adjective Pairs	Emotional Reaction	Adjective Pairs	Emotional Reaction
annoying/enjoyable	Pleasant	unpleasant/pleasant	Pleasant
bad/good	Good	ugly/attractive	Attractive
unlikable/pleasing	Pleasant	disagreeable/likeable	Pleasant
unpleasant/pleasant	Pleasant	rejecting/inviting	Inviting
unattractive/attractive	Attractive	bad/good	Good
unfriendly/friendly	Pleasant	repelling/appealing	Attractive
		discouraging/motivating	Motivating

## Data Availability

The data presented in this study are available in supplementary material here.

## Supplementary Materials

The following are available online at https://www.mdpi.com/article/10.3390/s21103480/s1.
