# Supplementary material for: Are UX Evaluation Methods Providing the Same Big Picture?
Source: Sensors (Basel). 2021 May 17;21(10):3480. doi: 10.3390/s21103480 (PMC8156257; doi:10.3390/s21103480)
Supplement: Supplementary file 1 [file sensors-21-03480-s001.zip › sensors-1176676-supplementary.pdf]

sensors

MDPI

1. Introduction

This supplementary material presents all the raw data we obtained during the longitudinal UX evaluation presented in the paper entitled "*Are UX Evaluation Methods Providing the Same Big Picture?*". We divided this material into sections according to the steps we followed to analyze the data we obtained. In Section 2, we present all the raw data collected during this longitudinal study. In Section 3, we present the data we used to analyze and identify careless respondents among the participants who participated in all 3 rounds of the study. Section 4 shows the final dataset after the removal of careless respondents. In Section 5, we present the calculation of the mean for each UX dimension per method. Finally, Section 6 presents the data related to Sentence Completion method.

2. Raw Data

In this section we present all the raw data obtained during the longitudinal UX evaluation per method. Legends for each color representing the dimensions are provided on the footer. The participants were from four courses as follows: Materials Engineering (Mat Eng), Mathematics (Math), Physics (Phys), and Mechanical Engineering (Mec Eng).

UEQ Raw Data (All Participants)																												
ID	CLASS	ROUND	1	2	3	4	5	6	7	8	9	10	11	12	13	14	15	16	17	18	19	20	21	22	23	24	25	26
P1	Mat Eng	1	1	0	0	-2	0	0	2	-1	1	0	0	0	0	-1	-1	0	2	-2	0	2	-3	1	1	-2	-1	3
P1	Mat Eng	2	-2	-2	0	-3	1	-1	2	0	0	0	0	-1	-3	0	0	0	0	-3	0	1	-2	1	0	-2	-1	0
P2	Math	1	-2	-2	2	2	2	-2	-2	-2	2	2	-2	2	-2	-2	-2	-2	2	2	2	-2	2	-2	2	2	2	-2
P3	Phys	1	3	3	3	3	3	3	3	-1	2	3	3	3	3	3	3	3	3	3	3	3	3	3	3	3	3	3
P3	Phys	2	3	3	3	2	3	2	3	0	3	3	3	3	3	3	3	3	3	3	3	3	2	3	3	3	3	3
P3	Phys	3	2	2	2	2	2	2	2	-1	1	2	2	2	0	1	2	2	2	1	2	2	2	2	2	2	2	2
P4	Phys	1	0	3	3	0	2	1	2	-2	0	2	2	2	-2	2	2	2	2	1	2	2	2	2	2	2	2	-2
P4	Phys	2	2	0	2	1	1	1	2	0	-1	1	0	1	0	2	1	0	0	1	1	0	-1	2	-1	2	1	2
P5	Phys	1	2	-2	0	-3	0	-1	-1	0	0	-1	-3	-2	-3	0	2	0	0	-2	0	0	-2	0	0	-1	-1	2
P5	Phys	2	1	0	-2	-3	1	1	0	-2	0	0	-1	0	-3	3	1	0	0	-3	0	1	-2	0	1	0	1	3
P5	Phys	3	0	-2	0	-3	-1	-2	0	-3	0	1	-3	-3	-3	1	2	0	0	-3	0	0	-2	0	0	-1	0	0
P6	Math	1	2	-1	0	-1	1	1	1	1	2	1	1	2	1	1	1	1	2	2	2	1	2	1	2	2	2	1
P6	Math	2	0	0	0	0	0	0	0	0	0	0	0	0	0	0	0	0	0	0	0	0	0	0	0	0	0	0
P6	Math	3	-2	-2	1	-3	0	-1	-2	-3	-1	0	-2	0	-3	-2	0	-2	-1	-3	-1	0	-2	0	0	0	-1	0
P7	Math	1	1	2	3	2	2	1	0	0	1	1	2	2	1	1	2	1	0	1	0	2	2	1	2	2	3	3

UEQ Raw Data (All Participants)																												
ID	CLASS	ROUND	1	2	3	4	5	6	7	8	9	10	11	12	13	14	15	16	17	18	19	20	21	22	23	24	25	26
P7	Math	2	2	2	1	2	3	2	2	0	0	0	2	0	1	1	3	1	1	1	0	0	2	2	2	2	2	0
P8	Math	1	2	3	3	3	2	1	2	1	3	-2	3	3	3	3	3	2	3	2	3	2	3	3	3	2	2	2
P8	Math	2	0	0	0	0	0	0	0	0	0	0	0	0	0	0	0	0	0	0	0	0	0	0	0	0	0	3
P8	Math	3	1	1	2	2	-3	1	1	-1	2	1	2	3	2	3	3	3	3	1	2	2	2	2	3	1	2	2
P9	Math	1	0	1	-1	0	2	1	1	1	-3	-3	1	3	-2	0	0	2	2	2	3	3	0	3	3	3	0	0
P9	Math	2	0	0	0	0	0	0	0	0	0	0	0	0	0	0	0	0	0	0	0	0	0	0	0	0	0	0
P9	Math	3	0	0	0	0	0	0	0	0	0	0	0	0	0	0	0	0	0	0	0	0	0	0	0	0	0	0
P10	Math	1	2	2	2	0	3	2	2	1	1	0	-2	3	-2	1	1	2	1	3	2	3	1	2	2	2	2	0
P10	Math	2	2	1	2	1	3	1	3	2	2	2	-2	2	1	2	1	1	2	3	2	3	2	2	2	2	2	0
P10	Math	3	1	1	0	0	2	0	1	0	1	0	-2	2	-2	1	1	1	-1	1	0	1	0	0	2	0	0	0
P11	Math	1	0	0	0	0	1	0	2	0	-1	0	0	1	-3	0	0	0	2	0	0	0	1	3	-1	1	1	-1
P12	Mec Eng	1	0	-1	1	-2	1	2	0	-2	0	0	2	1	-1	1	2	2	-1	0	-1	2	-2	-1	-1	-1	-1	1
P12	Mec Eng	2	2	1	1	2	2	0	2	0	2	2	2	2	0	0	2	1	2	1	0	1	0	1	0	1	2	-1
P12	Mec Eng	3	0	0	1	1	1	0	1	-1	1	2	1	0	0	0	1	1	0	0	1	1	0	1	1	1	1	1
P13	Mat Eng	1	3	0	3	0	0	1	1	-2	3	3	0	3	0	0	2	0	0	0	2	2	2	0	0	0	0	0
P13	Mat Eng	2	0	0	0	0	0	0	0	0	0	0	0	0	0	0	0	0	0	0	0	0	0	0	0	0	0	0
P14	Phys	1	3	3	0	3	3	1	3	-1	3	3	3	3	3	3	3	3	2	3	3	3	3	3	3	3	3	3
P15	Math	1	3	2	-2	0	0	1	2	2	-1	-1	1	2	-1	1	2	2	1	0	-1	1	1	1	1	2	2	-2
P15	Math	2	1	1	1	0	1	0	1	0	1	-1	1	1	1	0	1	1	1	0	1	1	0	1	1	1	1	1
P15	Math	3	3	3	1	0	-1	-1	1	-1	-1	-1	1	1	1	1	1	1	-1	0	0	1	-1	1	0	0	-1	1
P16	Math	1	0	0	0	0	0	0	0	0	0	0	0	0	0	0	0	0	0	0	0	0	0	0	0	0	0	0
P16	Math	2	3	3	-3	-3	-3	3	3	3	-3	-3	3	-3	3	3	3	3	-3	-3	-3	3	-3	3	-3	-3	-3	3
P16	Math	3	3	3	-3	-3	-3	3	3	3	-3	-3	3	-3	3	3	3	3	-3	-3	-3	3	-3	3	-3	-3	-3	3
P17	Mat Eng	1	-1	-1	2	0	0	-1	0	0	2	2	-2	1	0	1	2	0	2	1	0	1	-1	2	2	2	1	2
P17	Mat Eng	2	-1	-1	2	0	1	-1	0	0	2	2	0	0	1	0	2	0	1	0	1	1	-2	2	2	2	0	2
P17	Mat Eng	3	-1	0	-1	0	1	-1	1	1	2	1	1	1	-1	0	1	0	1	-1	0	1	-1	1	1	1	0	1
P18	Mat Eng	1	2	2	0	2	2	0	2	0	2	3	2	2	1	2	2	2	2	2	2	2	0	1	2	1	2	2
P18	Mat Eng	2	0	0	0	0	0	0	0	0	0	0	0	0	0	0	0	0	0	0	0	0	0	0	0	0	0	0

UEQ Raw Data (All Participants)

ID	CLASS	ROUND	1	2	3	4	5	6	7	8	9	10	11	12	13	14	15	16	17	18	19	20	21	22	23	24	25	26
P18	Mat Eng	3	-3	-3	2	1	2	-1	-1	0	0	0	0	0	0	0	0	0	0	0	0	0	0	0	0	0	0	0
P19	Mat Eng	1	3	1	-3	0	3	3	3	0	3	3	3	3	-1	3	3	3	3	3	3	3	2	3	3	3	3	3
P19	Mat Eng	2	3	2	3	0	3	2	3	0	2	3	3	3	-1	3	3	1	3	3	2	2	0	3	3	3	3	3
P19	Mat Eng	3	-2	-2	3	-3	3	-2	2	-3	0	3	3	0	-3	0	3	-3	3	0	0	3	-3	3	3	3	0	3
P20	Mat Eng	1	1	1	-1	-1	-1	1	2	0	0	-1	1	-1	0	1	1	1	2	2	1	1	1	1	2	1	1	1
P20	Mat Eng	2	0	0	0	0	0	0	0	0	0	0	1	-1	1	1	0	0	0	0	0	0	1	0	0	0	1	0
P20	Mat Eng	3	0	0	-1	-1	2	-1	1	1	0	1	0	-1	1	0	-1	-1	1	1	1	-1	1	-1	1	0	0	1
P21	Mat Eng	1	1	3	3	2	2	1	2	2	-2	3	-2	3	2	2	2	2	3	3	3	3	3	3	3	3	3	3
P21	Mat Eng	2	2	3	2	2	2	2	3	2	3	3	3	3	3	3	3	3	3	3	3	3	3	3	3	3	3	3
P22	Mat Eng	1	-3	-2	3	2	2	-2	-2	-2	2	2	-1	1	-2	-2	-2	-3	2	2	2	-2	3	-2	3	1	2	-3
P22	Mat Eng	2	2	0	-2	0	3	2	1	0	0	0	0	-1	-1	2	2	2	3	1	0	0	3	0	0	3	3	-3
P22	Mat Eng	3	-1	-2	-3	1	-2	-1	-1	3	-1	2	-3	2	-1	0	2	2	3	-1	3	2	-1	-1	3	-1	-1	1
P23	Mat Eng	1	1	1	1	2	2	1	1	-2	2	1	2	1	2	1	1	1	2	1	2	2	2	1	2	1	2	1
P23	Mat Eng	2	1	2	-2	-1	0	2	1	0	-1	-1	2	0	1	2	0	1	-1	-1	0	0	-1	1	0	-1	0	2
P23	Mat Eng	3	2	2	-2	-2	-2	1	0	0	-1	0	0	0	1	0	1	1	0	0	-1	0	0	1	-1	0	-1	1
P24	Mat Eng	1	2	2	3	1	3	2	2	2	2	3	2	3	-1	2	2	2	2	2	1	3	0	3	2	2	2	2
P24	Mat Eng	2	0	-1	1	-2	1	-1	0	-2	2	1	-2	0	-3	0	1	-1	0	-1	-1	0	-2	-1	0	-2	-3	2
P24	Mat Eng	3	0	1	3	-2	3	-1	2	-2	3	2	1	3	-1	2	3	2	3	2	-1	2	-2	3	1	2	2	-3
P25	Mat Eng	1	1	1	1	2	2	0	1	2	1	1	1	1	2	1	1	1	1	2	2	2	1	1	1	2	1	0
P25	Mat Eng	2	-1	0	-1	1	0	0	1	0	-1	0	-1	1	1	1	1	0	0	2	1	2	1	1	1	1	1	1
P25	Mat Eng	3	-1	0	1	1	1	1	1	1	0	1	1	1	0	1	0	0	0	1	1	1	1	1	1	1	1	1
P26	Mat Eng	1	0	0	0	0	0	0	0	0	0	0	0	0	0	0	0	0	0	0	0	0	0	0	0	0	0	0
P27	Mat Eng	1	0	1	1	1	1	2	2	-3	3	1	1	2	0	0	2	0	0	1	0	2	0	1	2	0	-3	0
P27	Mat Eng	2	0	0	0	0	0	0	0	0	0	0	0	0	0	0	0	0	0	0	0	0	0	0	0	0	0	0
P28	Mat Eng	1	2	2	1	1	2	1	2	-2	-1	3	3	3	0	2	3	2	2	2	2	3	-1	2	1	2	2	3
P28	Mat Eng	2	-1	-1	-1	1	3	-1	1	0	1	1	0	1	0	1	1	2	1	-1	1	1	-1	-1	1	0	1	2
P28	Mat Eng	3	1	1	-1	1	1	-1	1	1	1	3	2	3	1	1	3	2	1	1	2	1	1	1	2	1	1	3
P29	Mat Eng	1	2	1	1	0	1	0	2	1	2	2	2	2	1	1	1	1	1	2	1	2	0	1	2	1	2	-2

UEQ Raw Data (All Participants)																												
ID	CLASS	ROUND	1	2	3	4	5	6	7	8	9	10	11	12	13	14	15	16	17	18	19	20	21	22	23	24	25	26
P29	Mat Eng	2	0	0	2	0	0	0	0	0	1	0	0	1	0	0	0	0	0	1	0	0	0	0	0	0	0	0
P30	Mat Eng	1	3	3	0	-3	0	1	1	0	-1	-1	1	3	-1	3	3	3	3	3	3	3	0	3	3	3	0	-3
P30	Mat Eng	2	0	-2	-3	-2	-2	1	3	3	3	3	3	3	3	3	1	3	3	3	0	3	-2	3	3	-1	3	3
P30	Mat Eng	3	1	3	-1	-3	-1	1	2	1	2	0	0	-1	2	0	3	0	3	-1	2	1	-3	-2	1	0	-1	3
P31	Mat Eng	1	3	3	-3	-3	-3	3	3	3	-3	-3	3	-3	3	3	3	3	-3	-3	-3	3	-1	3	3	0	-3	3
P31	Mat Eng	2	2	2	-2	-2	-2	2	2	2	-2	-2	2	-2	2	2	2	2	-2	-2	-2	2	-2	2	-2	-2	-2	2
P32	Mat Eng	1	2	2	0	1	2	1	1	0	2	0	0	3	1	2	0	2	3	0	2	3	2	3	3	3	3	0
P32	Mat Eng	2	2	2	2	1	2	2	3	1	2	2	2	2	2	0	3	1	3	2	3	3	2	3	3	2	3	0
P33	Mat Eng	1	0	0	0	0	0	0	0	0	0	0	0	0	0	0	0	0	0	0	0	0	0	0	0	0	0	0
P33	Mat Eng	2	-1	-1	1	1	1	-1	-1	-1	1	1	-1	1	-1	-1	-1	-1	1	1	1	-1	1	-1	1	1	1	-1
P33	Mat Eng	3	0	0	0	0	0	0	0	-2	2	2	-2	-3	-3	-3	-3	-3	0	0	1	1	0	0	2	-1	-3	0
P34	Mat Eng	1	-1	1	0	-1	3	-1	0	0	1	2	2	2	0	1	1	1	2	1	1	2	0	1	2	1	1	3
P35	Mat Eng	1	1	2	-2	-1	-2	1	2	0	-2	-1	0	3	-1	2	0	2	0	2	2	2	1	2	2	2	2	2
P35	Mat Eng	2	-1	0	-2	-2	0	-2	-1	-3	0	-2	0	1	-2	0	0	1	1	1	0	1	-1	1	-1	1	1	1
P36	Phys	1	2	2	2	1	2	0	2	0	0	2	2	2	2	2	2	2	2	2	2	2	-2	2	-2	2	2	2
P37	Phys	1	1	1	1	0	1	1	1	0	-1	2	1	2	1	1	2	1	-1	1	1	1	1	2	2	1	2	2
P38	Phys	1	2	2	2	2	1	2	2	0	0	2	1	3	1	2	2	2	1	1	2	2	0	2	1	2	0	-1
P39	Phys	1	3	2	3	1	1	0	2	0	0	2	3	3	2	2	1	0	1	2	3	2	1	2	2	3	3	3
P40	Phys	1	2	2	-2	-2	1	0	2	0	2	2	2	2	1	2	1	2	1	2	2	2	2	2	2	2	2	2
P40	Phys	2	3	3	3	3	3	0	3	1	3	3	3	3	3	3	3	3	3	3	3	3	3	3	3	3	3	3
P40	Phys	3	2	2	2	2	2	0	2	1	2	2	3	3	2	2	2	2	2	2	2	2	2	2	2	2	2	2
P41	Phys	1	1	-1	2	1	0	0	0	0	-2	1	0	0	0	0	0	0	0	0	0	0	0	0	0	0	0	0
P41	Phys	2	1	1	1	1	0	0	1	0	-1	1	0	1	1	1	0	0	0	-1	0	1	1	1	1	0	0	0
P42	Phys	1	2	1	-2	0	-1	3	0	-1	1	0	1	1	1	1	-2	1	-1	1	-1	-1	-1	-1	-2	-3	2	2
P43	Phys	1	2	2	3	3	3	1	2	-2	2	3	3	2	3	3	2	2	3	3	2	3	3	3	3	2	3	3
P43	Phys	2	-3	2	1	-2	3	2	-3	2	1	-1	-2	-2	-3	3	-2	0	2	-2	3	2	1	-2	2	-2	-1	-1
P44	Phys	1	1	1	0	0	0	-1	0	2	-2	0	0	0	1	1	0	0	1	-1	1	0	-1	2	0	0	1	0
P44	Phys	2	-3	-3	0	0	0	0	-2	-2	-2	0	-2	-2	-2	-2	-1	-2	-1	-3	0	-1	-1	-1	-1	0	-3	0

UEQ Raw Data (All Participants)																												
**ID**	**CLASS**	**ROUND**	**1**	**2**	**3**	**4**	**5**	**6**	**7**	**8**	**9**	**10**	**11**	**12**	**13**	**14**	**15**	**16**	**17**	**18**	**19**	**20**	**21**	**22**	**23**	**24**	**25**	**26**
P44	Phys	3	-1	0	1	1	0	0	0	-2	0	0	-1	-1	0	-1	0	-1	-2	-3	0	0	-2	0	-1	1	1	0
P45	Phys	1	2	2	2	1	2	0	1	0	1	1	1	1	2	2	1	1	0	1	0	2	2	2	1	1	3	-3
P45	Phys	2	0	0	0	0	0	0	0	0	0	0	0	0	0	0	0	0	0	0	0	0	0	0	0	0	0	0
P45	Phys	3	1	1	1	2	2	0	1	0	0	1	0	1	1	1	0	1	1	2	1	2	2	2	2	2	2	-2
P46	Phys	1	2	1	3	2	0	0	1	2	1	2	2	2	0	1	1	1	1	2	2	2	2	2	2	0	2	-2
P47	Phys	1	2	2	2	1	2	2	2	1	1	2	2	2	1	2	2	2	2	2	2	2	1	2	2	2	2	2
P47	Phys	2	2	2	2	2	1	1	2	0	-1	2	2	2	1	2	2	1	2	3	2	2	2	2	2	2	2	1
P47	Phys	3	2	1	1	1	2	1	1	2	2	2	1	2	0	2	2	2	2	0	1	1	1	2	2	2	1	1
P48	Phys	1	1	1	1	-1	3	2	2	-2	2	-2	-2	2	-2	2	-3	2	2	2	3	2	-2	2	3	3	-2	-2
P49	Phys	1	2	-2	2	2	2	0	0	2	2	-1	3	3	-1	2	0	1	2	1	1	2	1	1	2	3	2	0
P49	Phys	2	1	1	1	2	2	0	0	0	0	2	0	2	-1	0	-1	2	1	0	0	2	1	1	2	2	2	0
P50	Phys	1	3	1	3	-2	2	2	3	-2	0	2	2	3	0	2	1	2	1	3	1	1	2	3	1	2	3	3
P51	Phys	1	0	0	1	0	0	1	2	1	2	2	-2	2	3	-1	0	2	3	3	3	-3	3	2	2	2	2	-2
P52	Phys	1	1	1	-1	1	1	-1	-1	-1	1	-3	-3	3	-3	-3	-3	-3	3	3	3	-3	3	-3	3	3	3	-3
P52	Phys	2	-1	-1	3	3	3	-1	-1	-1	1	3	-3	3	-1	-1	-1	-1	3	3	3	-1	3	-1	3	3	3	-3
P53	Math	1	1	2	3	0	1	3	1	0	2	2	2	2	1	2	3	3	2	2	2	2	3	2	-2	2	2	3
P54	Math	1	3	2	3	2	1	1	2	0	1	2	2	3	1	2	3	2	2	3	2	2	2	3	2	2	1	1
P54	Math	2	2	2	2	2	1	1	2	1	1	2	2	3	1	2	2	2	1	2	2	3	1	2	2	1	2	3
P54	Math	3	2	3	2	1	2	0	2	0	2	3	1	2	0	2	2	2	3	2	1	2	1	2	3	2	3	2
P55	Math	1	1	0	0	1	1	0	0	0	0	0	0	1	0	1	1	1	1	1	1	1	0	0	1	0	0	0
P55	Math	2	0	0	0	0	1	0	0	-1	0	0	0	0	0	0	0	0	0	1	0	0	0	0	1	0	0	0
P55	Math	3	1	1	2	0	1	2	1	-1	-1	2	0	2	-1	1	1	1	1	1	0	1	1	0	1	1	1	1
P56	Math	1	2	1	3	1	2	2	1	-1	2	3	3	3	0	2	3	3	3	3	2	3	1	2	3	2	2	3
P56	Math	2	1	0	3	1	2	2	2	1	2	3	1	2	-3	3	3	3	3	2	3	3	1	3	2	1	2	3
P56	Math	3	2	2	3	2	3	3	3	0	2	3	3	3	2	2	3	3	3	3	1	3	3	3	3	2	3	3
P57	Math	1	1	1	2	0	0	1	2	0	0	0	0	0	0	2	2	2	1	0	0	0	0	0	2	1	0	0
P57	Math	2	3	2	-2	1	2	2	3	2	-1	1	2	2	1	2	2	2	2	3	2	2	2	2	2	2	2	2
P57	Math	3	3	2	2	2	2	1	3	1	2	0	1	2	1	2	2	2	1	2	2	2	1	1	2	1	2	-1

UEQ Raw Data (All Participants)																												
ID	CLASS	ROUND	1	2	3	4	5	6	7	8	9	10	11	12	13	14	15	16	17	18	19	20	21	22	23	24	25	26
P58	Math	1	-3	-3	3	3	3	3	3	-3	-3	3	0	3	1	3	3	3	1	2	2	2	2	2	2	2	2	2
P59	Math	1	3	1	2	1	3	1	1	0	0	2	2	3	-1	1	1	1	2	2	3	2	2	1	2	2	2	2
P59	Math	2	2	1	1	1	1	1	1	0	0	0	0	1	0	2	2	2	2	1	1	1	0	1	2	1	1	2
P60	Math	1	3	3	1	-3	0	1	1	-1	0	1	0	1	-1	1	2	1	1	1	1	1	1	1	2	2	2	-2
P60	Math	2	3	-2	2	-3	2	2	2	-3	2	2	-2	2	-3	3	3	2	2	3	2	2	0	3	3	3	3	3
P60	Math	3	1	-1	1	-3	0	1	1	-1	0	1	-3	2	-3	1	2	1	3	0	-2	-1	-3	-3	0	1	2	0
P61	Math	1	0	2	2	1	2	2	1	1	1	2	2	2	2	2	1	1	2	2	1	2	2	2	3	2	2	-2
P61	Math	2	2	2	2	1	2	1	1	2	2	2	1	2	-1	1	0	1	0	-1	-3	1	1	1	1	0	1	-1
P61	Math	3	0	2	2	1	1	0	1	1	1	1	1	1	2	2	2	2	1	1	1	1	1	1	1	1	1	1
P62	Math	1	3	2	3	1	2	0	2	-2	0	2	1	3	-1	3	2	2	0	3	1	0	-1	3	3	2	1	1
P63	Math	1	3	3	3	2	3	3	3	3	2	3	2	3	2	3	3	3	3	3	3	3	3	3	3	3	3	-3
P63	Math	2	0	0	0	0	0	0	0	0	0	0	0	0	0	0	0	0	0	0	0	0	0	0	0	0	0	0
P63	Math	3	2	2	2	3	3	3	3	-2	-2	0	0	1	-1	0	1	2	2	0	0	0	0	0	0	0	0	0
P64	Mec Eng	1	3	2	2	2	3	2	3	1	0	2	2	3	2	2	2	3	1	3	2	1	2	2	2	3	3	2
P64	Mec Eng	2	1	2	1	1	2	0	1	0	2	2	3	2	2	1	2	3	3	2	2	2	2	3	3	2	2	2
P64	Mec Eng	3	0	1	1	0	2	0	1	-1	1	2	3	2	1	1	2	2	2	1	1	1	1	1	2	2	1	1
P65	Mec Eng	1	2	2	1	1	2	1	2	0	-1	1	0	1	0	1	1	2	1	0	1	2	1	0	1	2	2	1
P65	Mec Eng	2	1	0	0	1	1	1	0	0	1	1	1	1	1	1	-1	0	1	1	1	1	1	1	1	1	1	1
P65	Mec Eng	3	1	0	-1	-1	-1	1	1	1	-1	-1	1	-1	1	1	1	1	-1	-1	-1	1	-1	1	-1	-1	-1	1
P66	Mec Eng	1	1	2	2	1	1	1	1	0	2	2	2	2	1	2	1	1	3	1	1	2	1	2	2	1	1	2
P66	Mec Eng	2	1	1	1	0	1	0	0	0	1	1	0	1	0	1	1	1	2	1	1	1	1	1	1	1	1	1
P67	Mec Eng	1	1	1	1	0	2	0	1	1	0	1	1	2	0	2	1	2	0	1	1	2	0	2	-2	1	1	2
P67	Mec Eng	2	1	2	2	2	2	0	2	1	0	2	2	2	2	1	2	2	0	1	0	1	1	2	2	0	0	2
P67	Mec Eng	3	0	0	0	0	0	0	0	0	0	0	0	0	0	0	0	0	0	0	0	0	0	0	0	0	0	0
P68	Mec Eng	1	1	1	0	1	0	0	1	0	0	1	1	2	1	1	-1	1	-1	0	1	1	1	1	2	2	2	1
P68	Mec Eng	2	-2	-2	2	1	2	-1	-1	0	1	0	-1	2	-3	-1	-2	-1	0	-1	-1	0	0	1	2	1	1	0
P68	Mec Eng	3	0	0	1	2	1	0	1	0	0	1	-1	0	1	1	0	1	-1	-1	0	1	0	1	1	0	0	-1
P69	Mec Eng	1	-3	-3	-3	0	3	-3	-2	3	3	0	-3	2	-3	-3	2	-2	3	-3	-2	-2	-3	-3	-2	-2	-3	0

UEQ Raw Data (All Participants)																												
ID	CLASS	ROUND	1	2	3	4	5	6	7	8	9	10	11	12	13	14	15	16	17	18	19	20	21	22	23	24	25	26
P69	Mec Eng	2	-2	3	-2	-2	3	-2	-1	3	3	3	-2	-2	-3	-2	-2	-2	3	-1	2	3	3	-3	3	-2	-2	-3
P69	Mec Eng	3	-1	-1	1	1	1	-1	-1	-1	1	1	0	0	0	0	0	0	0	0	0	0	0	0	0	0	0	0
P70	Mec Eng	1	3	2	2	2	3	2	2	-2	2	2	-2	3	2	3	3	3	0	2	2	3	2	3	0	3	2	3
P70	Mec Eng	2	3	3	3	-3	1	3	3	0	3	3	3	3	-1	3	3	2	1	2	3	3	3	3	1	1	1	2
P70	Mec Eng	3	0	0	0	0	0	0	0	0	0	0	0	0	0	0	0	0	0	0	0	0	0	0	0	0	0	0
P71	Mec Eng	1	0	0	0	0	0	0	0	0	0	0	0	0	0	0	0	0	0	0	0	0	0	0	0	0	0	0
P72	Mec Eng	1	1	1	2	0	0	-1	-2	-1	1	1	1	2	1	1	1	1	-1	1	1	1	1	1	-1	-1	1	1
P72	Mec Eng	2	1	-1	1	1	1	1	1	1	1	1	1	2	2	1	2	2	1	1	2	2	-1	2	1	1	1	2
P72	Mec Eng	3	-1	0	-1	-1	3	1	0	0	0	1	-2	1	-1	0	1	-1	0	0	0	1	-2	-1	1	1	-1	1
P73	Mec Eng	1	0	0	0	0	0	0	0	0	1	1	-1	1	-1	1	0	0	1	0	0	1	-1	0	0	0	0	1
P73	Mec Eng	2	-1	-1	0	-2	0	-1	-1	0	0	0	-1	0	-2	0	0	-1	1	-2	0	0	-3	-1	0	0	-1	1
P73	Mec Eng	3	-1	-1	0	-1	0	-1	-1	0	0	0	-1	1	-2	1	0	-1	0	-1	0	0	-1	0	0	0	0	0
P74	Mec Eng	1	3	3	2	2	3	2	3	0	0	3	2	3	1	2	2	2	3	3	3	3	2	2	3	3	2	2
P74	Mec Eng	2	2	2	1	2	2	1	2	-1	1	2	1	2	1	2	2	2	2	1	2	2	2	2	2	2	2	2
P74	Mec Eng	3	1	1	1	1	2	2	2	0	0	3	2	3	1	1	3	2	2	2	2	2	2	2	2	2	2	3
P75	Mec Eng	1	2	2	1	1	1	1	1	-1	1	1	1	2	1	1	1	1	1	2	1	1	1	1	1	1	1	-1
P75	Mec Eng	2	1	2	2	2	2	1	2	1	2	2	1	2	1	1	0	2	1	1	2	2	1	2	2	2	2	0
P75	Mec Eng	3	2	2	1	1	1	1	2	0	1	1	1	1	1	1	1	1	1	1	2	2	1	2	2	1	1	1
P76	Mec Eng	1	0	1	1	-1	0	1	1	0	1	0	0	1	-1	0	1	1	-1	0	0	1	-1	0	1	0	1	0
P76	Mec Eng	2	1	1	1	0	1	0	1	-1	1	0	1	1	1	1	1	1	0	0	0	1	0	1	1	2	2	1
P77	Mec Eng	1	2	2	2	3	3	0	2	-3	2	3	3	3	2	3	3	3	3	3	3	2	3	3	3	3	3	3
P77	Mec Eng	2	2	3	2	2	3	2	3	2	2	-1	3	3	2	3	3	3	2	3	3	2	3	3	2	3	3	2
P77	Mec Eng	3	0	3	2	2	3	1	2	-2	2	2	3	2	0	3	3	3	3	1	2	3	3	3	2	2	3	3
P78	Mec Eng	1	2	2	2	1	3	2	3	1	1	3	1	3	0	2	3	2	2	2	2	2	2	2	3	3	3	3
P78	Mec Eng	2	3	3	3	2	3	2	3	0	2	3	3	3	0	3	3	3	3	2	3	3	2	3	2	2	2	3
P78	Mec Eng	3	3	3	3	3	3	3	3	0	3	3	3	2	0	0	3	3	2	3	3	2	3	3	2	2	2	-2
P79	Mec Eng	1	2	2	2	3	1	2	3	1	2	-2	3	3	1	2	2	3	3	3	2	2	2	2	1	3	3	0
P79	Mec Eng	2	3	3	-3	-3	-3	2	1	2	0	-3	2	1	2	0	1	2	-1	1	0	2	-1	2	-1	-2	-2	2

UEQ Raw Data (All Participants)																												
ID	CLASS	ROUND	1	2	3	4	5	6	7	8	9	10	11	12	13	14	15	16	17	18	19	20	21	22	23	24	25	26
P79	Mec Eng	3	3	3	-3	-3	-3	3	3	3	-3	-3	3	-3	3	3	3	3	-3	-3	-3	3	-3	3	-3	-3	-3	3
P80	Mec Eng	1	1	1	1	1	2	0	2	0	1	2	-2	2	-1	-1	1	1	0	-1	0	1	1	1	-1	-2	0	0
P80	Mec Eng	2	0	-1	2	0	0	0	0	0	1	1	0	1	1	0	0	0	0	0	0	1	-1	1	1	0	1	-1
P80	Mec Eng	3	1	2	1	0	1	1	1	0	3	2	2	3	1	2	3	2	2	2	3	2	1	3	3	2	3	-2
P81	Mec Eng	1	3	3	2	2	1	1	2	0	-1	0	2	2	1	1	0	2	-2	1	2	0	2	1	2	2	2	0
P81	Mec Eng	2	2	2	1	3	1	0	2	1	1	1	2	2	2	2	1	1	1	2	2	1	1	0	1	1	1	1
P81	Mec Eng	3	0	0	0	0	0	0	0	0	0	0	0	0	0	0	0	0	0	0	0	0	0	0	0	0	0	0
P82	Mec Eng	1	3	3	3	2	3	2	3	0	-1	3	2	2	1	1	3	3	2	3	0	2	2	2	2	2	2	2
P82	Mec Eng	2	-2	0	0	2	2	1	1	0	-2	2	2	2	1	1	1	1	2	2	2	0	1	1	2	2	2	1
P82	Mec Eng	3	-2	-2	3	1	1	-1	-1	-1	1	1	-1	1	-1	-1	0	0	0	0	0	0	0	0	0	0	0	0
P83	Math	1	1	-1	0	3	3	3	0	2	2	-1	0	1	-2	-2	-3	-2	1	0	-1	2	-3	2	-1	0	1	-2
P84	Mec Eng	1	2	1	2	1	3	2	2	2	2	1	2	2	-1	2	2	1	2	2	2	2	1	2	2	2	2	2
P85	Mat Eng	1	3	0	3	0	2	2	3	0	2	2	2	3	0	3	3	2	2	3	2	2	0	2	2	2	1	3
P85	Mat Eng	2	1	0	3	-1	2	1	3	0	1	3	0	3	-1	3	3	1	3	3	2	2	-1	3	2	3	1	3
P85	Mat Eng	3	1	-1	2	-1	1	0	1	-1	1	3	1	2	-2	0	3	0	2	2	1	2	-1	0	2	1	0	2
P86	Math	1	2	0	1	0	3	-2	3	-1	1	2	2	1	1	3	2	2	1	2	3	3	0	1	3	3	2	1
P86	Math	2	1	0	-1	0	-1	0	-1	0	-1	0	0	-1	0	-1	0	1	-1	-1	0	0	0	1	0	0	0	0
P86	Math	3	0	0	0	0	0	0	0	0	0	0	0	0	0	0	0	0	0	0	0	0	0	0	0	0	0	0
P87	Mat Eng	1	3	3	3	3	3	3	3	3	3	-3	3	3	3	3	3	3	3	3	3	3	3	3	3	3	3	3
P87	Mat Eng	2	3	3	3	3	3	3	3	-3	3	3	3	3	3	3	2	3	3	3	3	3	3	3	3	3	3	3
P88	Phys	1	2	2	2	2	2	0	2	-1	2	2	2	2	-2	1	1	1	2	2	2	3	2	3	1	1	2	3
P89	Phys	1	0	0	0	0	0	0	0	0	0	0	0	0	0	0	0	0	0	0	0	0	0	0	0	0	0	0
P90	Math	1	2	-3	3	3	2	-1	3	3	3	3	0	3	-3	3	3	3	3	3	3	3	-3	3	3	3	3	-3
P90	Math	2	3	3	3	-3	3	3	3	3	3	3	3	3	3	3	3	3	3	3	3	3	3	3	3	3	3	3
P91	Mec Eng	1	1	1	-2	-2	2	0	0	0	3	2	2	2	2	2	2	2	0	2	0	0	0	0	0	0	0	0
P92	Mat Eng	1	1	1	2	1	2	0	0	0	2	1	3	2	2	2	1	3	1	2	3	2	2	3	3	0	2	1
P92	Mat Eng	2	0	1	1	2	1	0	1	1	-1	1	2	3	1	2	1	1	1	2	1	2	1	-2	2	1	-1	-1
P92	Mat Eng	3	0	0	0	0	0	0	0	0	0	0	0	0	0	0	0	0	0	0	0	0	0	0	0	0	0	0

UEQ Raw Data (All Participants)																												
ID	CLASS	ROUND	1	2	3	4	5	6	7	8	9	10	11	12	13	14	15	16	17	18	19	20	21	22	23	24	25	26
P93	Mec Eng	1	1	1	2	0	0	1	1	0	1	1	0	1	-1	1	1	1	0	1	0	1	0	1	1	1	1	0
P93	Mec Eng	2	2	0	0	1	0	1	1	0	0	-1	0	0	2	2	1	1	0	0	3	1	0	2	2	1	2	0
P93	Mec Eng	3	0	0	0	0	0	0	0	0	0	0	0	0	0	0	0	0	0	0	0	0	0	0	0	0	0	0
P94	Mec Eng	1	0	-1	-2	-3	1	-2	0	2	0	0	0	0	-1	0	0	0	0	0	0	0	-1	0	0	0	0	0
P94	Mec Eng	2	0	1	0	1	0	0	0	3	3	0	0	2	-1	0	0	0	2	-1	2	1	1	1	0	0	0	0
P94	Mec Eng	3	-1	-1	0	-1	-2	-2	-1	1	-2	-1	0	1	1	-2	-1	0	0	-2	0	0	0	0	0	0	0	0
P95	Phys	1	1	0	1	2	1	-1	-1	0	2	0	1	0	1	0	-2	1	2	-1	-2	0	1	-3	1	2	-1	-1
P95	Phys	2	1	1	1	1	3	0	2	2	2	3	1	2	0	1	1	1	0	1	2	3	0	-2	1	0	1	1
P95	Phys	3	2	2	-2	-2	-2	2	2	2	-2	-2	2	-2	2	2	2	2	-2	-2	-2	2	-2	2	-2	-2	-2	2
P96	Phys	1	0	0	0	2	2	0	1	0	2	1	0	2	0	2	0	0	1	2	1	1	0	0	-1	0	2	0
P96	Phys	2	0	0	0	0	0	0	0	0	0	0	0	0	0	0	0	0	0	0	0	0	0	0	0	0	0	0
P97	Phys	1	-1	2	-2	2	1	0	1	1	1	1	2	1	0	0	2	1	0	0	0	1	2	1	2	1	1	2
P98	Mat Eng	1	0	1	1	1	2	1	2	0	1	3	1	2	1	1	2	1	-1	1	1	2	2	1	1	1	1	3
P99	Mat Eng	1	2	2	3	2	2	2	3	0	2	3	3	3	2	3	3	3	3	3	2	3	3	3	3	2	2	3
P99	Mat Eng	2	2	2	3	2	0	1	2	0	2	2	3	3	3	2	2	2	2	2	3	3	3	3	3	3	3	2
P99	Mat Eng	3	3	3	3	3	0	3	3	0	-1	3	3	3	3	3	3	0	3	3	3	3	3	3	3	3	3	3
P100	Mat Eng	1	0	0	-2	0	0	-3	-1	0	-1	0	-1	1	-1	0	-1	1	2	2	0	0	-2	0	-2	-1	0	0
P100	Mat Eng	2	0	0	0	0	0	0	0	0	0	0	0	0	0	0	0	0	0	0	0	0	0	0	0	0	0	0
P100	Mat Eng	3	0	-3	3	0	-1	-2	-2	-2	-1	0	0	0	-3	0	1	-2	-2	-3	0	0	0	0	0	-1	-3	2
P101	Mec Eng	1	0	1	1	0	0	0	1	-1	1	-1	1	1	1	1	-1	1	1	1	1	1	1	1	1	1	1	1
P101	Mec Eng	2	1	1	1	0	1	1	1	2	1	0	1	1	0	1	1	1	1	1	1	1	1	1	-1	1	1	1
P101	Mec Eng	3	1	1	1	0	0	1	1	0	1	-1	1	1	0	1	-1	1	0	1	1	1	1	1	-1	-1	1	1
P102	Mat Eng	1	1	0	0	0	0	1	0	1	1	-1	1	2	1	1	2	1	2	2	0	2	0	1	2	3	3	-2
P102	Mat Eng	2	0	0	0	0	0	0	0	0	0	0	0	0	0	0	0	0	0	0	0	0	0	0	0	0	0	0
P102	Mat Eng	3	-3	-3	-3	-3	-3	-3	-3	-3	-3	-3	-3	-3	-3	-3	-3	-3	-3	-3	-3	-3	-3	-3	-3	-3	-3	-3
P103	Math	1	0	0	0	0	0	0	0	0	0	0	0	0	0	0	0	0	0	0	0	0	0	0	0	0	0	0
P104	Mat Eng	1	0	0	2	2	2	0	2	0	2	0	2	2	0	2	0	2	0	2	0	0	0	3	3	3	2	2
P104	Mat Eng	2	1	1	0	1	1	0	1	1	2	0	1	1	0	1	1	1	0	1	1	1	0	2	2	2	1	1

UEQ Raw Data (All Participants)																												
ID	CLASS	ROUND	1	2	3	4	5	6	7	8	9	10	11	12	13	14	15	16	17	18	19	20	21	22	23	24	25	26
P104	Mat Eng	3	1	1	1	1	1	0	1	1	1	0	1	1	0	1	1	1	1	0	0	0	0	0	0	0	0	0

AttrakDiff RAW Data (All Participants)																														
ID	CLASS	ROUND	1	2	3	4	5	6	7	8	9	10	11	12	13	14	15	16	17	18	19	20	21	22	23	24	25	26	27	28
P105	Phys	1	0	1	1	1	0	1	1	1	1	1	1	-2	0	0	0	1	2	2	2	1	0	0	1	2	1	1	0	0
P105	Phys	2	0	0	0	0	1	2	0	2	2	-2	1	0	-2	-2	2	-1	-2	-2	1	-2	-2	1	1	-1	-2	1	2	-1
P105	Phys	3	-1	1	2	2	2	1	1	1	1	-2	1	1	0	0	0	1	1	1	2	1	0	0	0	1	0	1	0	0
P106	Phys	1	-1	1	1	-1	0	0	1	1	1	0	1	1	1	2	0	2	0	0	2	-1	1	0	0	0	0	0	0	0
P107	Mat Eng	1	-3	3	-1	0	0	1	3	-2	1	-1	3	1	0	2	-2	2	2	0	0	-2	0	0	0	2	2	2	-2	2
P107	Mat Eng	3	3	-3	3	3	3	3	-3	3	3	-3	3	3	-3	-3	3	-3	-3	-3	3	-3	-3	3	3	-3	-3	3	3	-3
P108	Math	1	2	-2	2	2	2	-2	-2	-2	-3	-1	1	1	-1	-1	1	-1	-1	-1	1	-1	-1	1	1	-1	-1	1	1	-1
P109	Math	1	-3	3	0	2	-3	0	3	3	3	0	2	0	3	3	3	3	0	1	3	3	0	0	3	3	3	0	0	2
P110	Math	1	0	-1	0	0	-2	-1	-1	-1	-1	-1	1	-1	0	0	-1	1	0	-1	-2	-2	-1	0	0	1	2	-1	0	0
P111	Math	1	-1	1	3	2	2	1	2	-2	-1	0	0	0	2	2	0	2	2	3	3	1	1	0	2	2	2	2	0	2
P111	Math	2	-1	0	2	1	0	1	3	2	2	0	2	-1	2	2	-1	2	2	2	2	1	2	0	2	2	2	2	1	2
P111	Math	3	-1	0	2	2	0	1	2	2	2	1	1	-1	2	2	0	2	2	1	2	2	2	0	2	2	2	2	0	2
P112	Math	1	3	2	3	0	3	3	0	3	3	0	0	0	0	3	3	1	0	0	2	0	0	0	0	0	0	1	0	0
P112	Math	2	0	0	0	0	0	0	0	0	0	0	0	0	0	0	0	0	0	0	0	0	0	0	0	0	0	0	0	0
P113	Phys	1	-1	1	1	0	0	2	1	2	2	1	2	0	2	2	2	1	1	1	2	2	1	2	2	1	1	2	2	2
P113	Phys	2	0	0	0	0	0	0	0	0	0	0	0	0	0	0	0	0	0	0	0	0	0	0	0	0	0	0	0	0
P114	Phys	1	0	-2	-2	-2	-2	-2	-1	-1	-1	-1	0	0	-2	0	-2	-2	-2	-2	-3	3	-2	0	0	0	-2	-3	-3	-1
P115	Phys	1	0	0	0	0	0	0	0	0	0	0	0	0	0	0	0	0	0	0	0	0	0	0	0	0	0	0	0	0
P115	Phys	2	2	2	2	2	2	-1	1	2	2	2	1	2	2	2	3	2	2	2	2	1	0	-1	1	0	2	2	1	1
P116	Phys	1	0	0	0	0	0	0	0	0	0	-1	1	1	-1	-1	1	-1	-1	-1	1	-1	-1	1	1	-1	-1	1	1	-1
P116	Phys	2	0	0	0	0	1	1	0	0	0	0	0	1	2	0	0	0	0	0	0	0	1	0	1	0	0	0	0	0
P116	Phys	3	-3	3	3	-1	0	2	1	1	1	2	2	1	1	1	1	0	0	2	2	1	2	1	1	-1	0	0	0	0
P117	Phys	1	0	0	-1	0	0	0	2	-2	0	3	1	1	2	0	0	2	2	2	1	2	0	0	1	0	0	0	0	0
P117	Phys	2	0	0	0	0	0	0	0	0	0	0	0	0	0	0	0	0	0	0	0	0	0	0	0	0	0	0	0	0
P117	Phys	3	1	0	2	1	3	-1	2	1	3	3	2	3	3	2	0	2	2	1	3	2	2	0	2	2	0	2	1	2
P118	Phys	1	0	0	0	0	0	0	0	0	0	0	0	0	0	0	0	0	0	0	0	0	0	0	0	0	0	0	0	0
P118	Phys	2	-3	0	-3	0	-3	1	-3	-3	-3	0	-1	-3	-1	0	0	0	-2	-1	-3	-3	-3	0	0	-3	0	-2	0	0
P118	Mec Eng	2	1	-1	-1	-1	0	0	0	0	0	0	0	0	0	0	0	0	0	0	0	0	0	0	0	0	0	0	0	0

AttrakDiff RAW Data (All Participants)

ID	CLASS	ROUND	1	2	3	4	5	6	7	8	9	10	11	12	13	14	15	16	17	18	19	20	21	22	23	24	25	26	27	28
P119	Mat Eng	1	-1	1	2	2	2	3	3	3	3	2	3	1	2	3	-1	3	3	3	1	1	0	0	1	1	3	0	-1	0
P120	Mat Eng	1	0	2	2	0	1	0	2	2	1	0	1	1	1	0	2	1	2	1	1	1	1	0	0	1	1	0	1	0
P120	Mat Eng	2	0	1	1	1	1	1	1	0	0	0	1	1	1	1	0	1	1	2	1	1	1	0	1	1	1	1	1	0
P121	Mat Eng	1	-1	2	2	2	1	1	2	2	3	2	1	1	2	2	3	2	2	2	3	2	1	1	2	1	2	2	1	2
P121	Mat Eng	2	-1	0	1	1	1	0	1	1	1	1	1	1	1	1	1	2	1	1	2	0	0	1	1	1	2	0	0	1
P121	Mat Eng	3	-1	1	2	2	1	1	2	1	1	1	1	1	2	2	2	2	2	2	2	2	1	1	1	2	2	2	1	1
P122	Mat Eng	1	-2	3	3	-3	0	0	3	3	3	2	2	0	2	0	0	2	0	2	3	0	0	0	0	3	1	0	0	0
P122	Mat Eng	2	0	1	0	1	0	0	0	1	1	0	1	0	1	0	0	1	1	1	1	1	-1	0	0	1	1	0	0	1
P122	Mat Eng	3	0	1	1	-1	0	0	0	1	1	0	0	1	0	0	0	1	0	0	1	0	0	0	0	0	0	0	0	0
P123	Mat Eng	1	0	2	3	1	1	1	3	3	2	2	3	1	2	2	2	2	2	1	3	3	0	0	1	1	0	1	0	3
P123	Mat Eng	2	0	0	0	0	0	0	0	0	0	0	0	0	0	0	0	0	0	0	0	0	0	0	0	0	0	0	0	0
P124	Mat Eng	1	-1	1	2	2	2	2	0	2	2	-2	0	1	1	3	1	0	2	2	2	2	2	-1	2	2	2	2	2	2
P124	Mat Eng	2	-1	1	0	1	-1	1	1	0	-1	-1	1	-1	1	-1	-3	1	1	1	1	-2	1	-1	-1	1	1	0	1	0
P124	Mat Eng	3	3	1	0	-1	-1	0	1	0	0	0	3	-1	0	1	0	2	-1	-1	-1	0	0	-3	-1	1	1	1	1	1
P125	Mat Eng	1	0	0	0	0	0	0	0	0	0	0	0	0	0	0	0	0	0	0	0	0	0	0	0	0	0	0	0	0
P125	Mat Eng	2	0	0	0	0	0	0	0	0	0	0	0	0	0	0	0	0	0	-1	-1	-1	1	1	-1	-1	1	1	0	0
P125	Mat Eng	3	0	0	0	0	0	0	0	0	0	0	0	0	0	0	0	0	0	0	0	0	0	0	0	0	0	0	0	0
P126	Mat Eng	1	1	1	0	-1	0	1	1	2	2	0	0	2	1	2	1	2	0	2	2	1	2	1	0	1	3	2	1	2
P126	Mat Eng	2	-2	-1	0	1	-1	2	-1	-1	-1	2	1	2	0	1	-1	1	-1	1	-1	1	-2	1	1	0	2	1	1	1
P126	Mat Eng	3	-1	0	1	0	-1	1	0	0	0	2	1	-1	2	1	-2	2	-1	2	1	0	-1	-1	-1	-1	3	-1	0	0
P127	Mat Eng	1	-1	0	1	0	0	1	1	1	2	0	0	0	1	3	1	3	0	2	3	0	0	3	1	0	1	1	2	0
P127	Mat Eng	2	-2	2	1	2	0	1	3	3	3	1	2	0	3	2	1	2	0	3	3	1	0	0	3	1	1	0	1	2
P127	Mat Eng	3	0	1	1	0	0	0	2	2	1	0	1	0	1	1	1	1	0	1	2	0	0	0	1	1	1	1	1	1
P128	Mat Eng	1	-1	1	-3	2	0	1	2	2	0	-1	2	2	0	1	0	1	1	1	-3	-3	-3	0	2	-2	3	-3	0	2
P128	Mat Eng	2	-2	2	-1	0	-2	-2	0	-1	0	0	0	0	0	1	-2	-1	0	0	0	0	0	1	-1	-1	-1	1	0	0
P129	Mat Eng	1	0	-2	2	3	3	2	2	2	0	0	3	-2	1	1	-2	1	2	1	2	-1	2	2	3	0	3	0	3	0
P129	Mat Eng	2	-2	-2	-1	0	0	1	0	-1	-1	2	0	-2	-2	0	-1	-2	-2	2	-2	0	-1	-3	-2	0	2	-1	1	-2
P129	Mat Eng	3	3	-3	3	3	3	3	-3	3	3	-3	3	3	-3	-3	3	-3	-3	-3	3	-3	-3	3	3	-3	3	-3	3	-3

AttrakDiff RAW Data (All Participants)																														
ID	CLASS	ROUND	1	2	3	4	5	6	7	8	9	10	11	12	13	14	15	16	17	18	19	20	21	22	23	24	25	26	27	28
P130	Mat Eng	1	0	2	3	0	0	3	0	3	0	2	0	0	3	3	1	2	0	-1	2	2	0	0	1	0	2	0	0	2
P130	Mat Eng	2	0	0	0	0	0	0	0	0	0	0	0	0	0	0	0	0	0	0	0	0	0	0	0	0	0	0	0	0
P130	Mat Eng	3	-1	2	3	2	3	3	3	3	3	3	0	0	3	3	0	3	0	2	3	3	0	0	2	0	2	3	3	2
P131	Phys	1	-3	-2	0	-3	-1	3	2	1	1	-2	2	0	3	1	3	2	3	3	3	0	0	2	2	0	3	1	-1	3
P131	Phys	2	-3	0	1	2	0	3	3	1	1	0	3	2	3	0	0	2	2	3	0	0	-1	2	2	0	3	0	1	1
P132	Phys	1	0	3	3	2	2	2	3	3	2	2	3	2	3	2	2	2	2	3	3	2	3	3	3	0	2	2	3	2
P132	Phys	2	3	2	3	-3	3	3	3	3	3	3	3	3	3	3	3	3	3	3	3	3	3	3	-3	3	3	3	-3	3
P132	Phys	3	3	3	3	3	2	3	3	3	3	3	3	3	3	3	3	3	3	3	3	3	3	3	3	2	2	2	2	2
P133	Phys	1	0	1	1	-1	-1	1	-1	0	0	0	1	0	0	0	0	0	0	0	1	-2	-1	1	2	0	-1	2	0	1
P133	Phys	2	-2	-1	0	-1	-1	1	0	0	1	-1	0	-1	0	-2	0	0	0	2	1	-1	-1	1	1	1	2	1	0	0
P133	Phys	3	0	0	0	0	0	0	0	0	0	0	0	0	0	0	0	0	0	0	0	0	0	0	0	0	0	0	0	0
P134	Phys	1	-1	-1	1	1	0	1	1	1	1	-1	0	0	1	1	-1	1	2	1	1	1	1	-1	1	1	2	1	1	1
P134	Phys	2	-1	1	1	1	-1	1	1	1	1	1	0	-1	1	1	0	1	1	1	1	1	1	1	1	2	1	1	1	1
P134	Phys	3	3	-3	3	3	3	3	-3	3	3	-3	3	3	-2	-1	0	1	2	3	-1	0	-2	3	1	-1	-1	1	1	-1
P135	Phys	1	-1	0	2	3	2	0	1	2	2	-1	3	0	1	3	0	3	1	2	3	1	0	0	1	2	2	1	0	3
P135	Phys	2	0	0	0	0	0	0	0	0	0	0	0	0	0	0	0	0	0	0	0	0	0	0	0	0	0	0	0	0
P135	Phys	3	-2	-2	1	2	-2	-1	1	1	1	-2	0	1	-2	-1	-2	0	-1	-1	0	0	0	-3	-3	-1	-3	1	-2	0
P136	Phys	1	0	1	-2	-2	-2	-2	0	-1	-2	-2	3	3	3	3	3	1	-3	0	2	0	2	-1	1	2	3	3	3	-2
P136	Phys	2	-3	-2	0	3	0	3	3	3	-1	-3	1	-3	0	1	-2	-1	-2	0	-1	-3	-1	-3	-2	-3	3	-3	3	3
P137	Phys	1	-1	1	2	2	2	2	2	2	2	0	2	-1	2	3	0	2	2	2	2	2	2	2	2	2	3	3	3	2
P137	Phys	2	-2	2	2	2	3	3	3	3	3	3	3	-2	3	3	2	3	3	3	2	3	3	2	2	3	3	3	3	3
P137	Phys	3	-2	2	2	3	-2	2	3	3	3	1	3	-2	3	3	2	3	3	3	3	2	2	2	2	2	2	2	2	2
P138	Phys	1	0	2	1	1	1	1	2	-1	2	1	2	2	2	1	1	1	1	1	1	1	1	2	2	1	-1	1	-2	1
P138	Phys	2	1	1	1	0	1	1	1	1	1	0	1	1	1	1	1	1	1	0	1	0	1	0	1	-1	-1	0	1	1
P138	Phys	3	1	1	1	1	0	1	2	2	1	1	1	1	1	1	2	2	2	2	1	3	1	1	1	2	2	2	2	2
P139	Phys	1	0	0	0	0	0	0	0	0	0	0	0	0	0	0	0	0	0	0	0	0	0	0	0	0	0	0	0	0
P140	Phys	1	-1	1	3	3	3	3	-2	2	2	-2	2	2	-2	-2	2	-2	-2	-2	2	-2	-2	2	0	0	-1	0	1	0
P140	Phys	2	-1	1	1	-1	2	2	1	2	2	0	0	0	2	2	0	2	2	0	3	3	1	1	1	2	1	2	1	1

AttrakDiff RAW Data (All Participants)																														
ID	CLASS	ROUND	1	2	3	4	5	6	7	8	9	10	11	12	13	14	15	16	17	18	19	20	21	22	23	24	25	26	27	28
P140	Phys	3	-2	-2	2	2	2	2	1	2	2	2	0	0	2	2	0	1	2	2	2	2	0	0	1	1	1	1	1	1
P141	Phys	1	0	0	0	0	-1	0	1	0	0	-1	0	-1	0	1	0	0	0	1	1	-1	0	0	1	-1	0	0	0	0
P141	Phys	2	-3	1	-1	-1	-3	1	1	1	-1	-2	1	1	1	-1	-1	1	1	1	1	-1	-1	-1	1	-2	1	1	1	1
P142	Phys	1	0	0	2	-1	0	2	2	2	2	2	-1	0	2	2	0	2	1	1	3	2	1	-1	3	0	1	2	2	0
P142	Phys	2	2	0	1	1	0	1	2	2	2	0	1	1	2	1	1	2	1	1	3	1	0	0	1	0	1	-1	1	1
P143	Phys	1	2	-2	2	1	1	1	-1	1	1	0	0	1	-1	-1	1	-1	-3	0	-3	-3	-3	-3	-3	-3	3	-3	-3	1
P143	Phys	2	3	-2	1	0	-1	-2	3	-2	-1	0	1	2	-3	-2	1	0	1	2	-3	2	1	0	1	-2	-3	2	1	0
P143	Phys	3	3	-3	3	3	3	3	-3	3	3	-3	3	3	-3	-3	3	-3	-3	-3	3	-3	-3	3	3	-3	3	3	3	-3
P144	Phys	1	-1	0	0	0	1	1	-1	1	1	1	0	0	-1	0	-1	1	0	0	1	0	0	-1	-1	0	0	0	0	0
P144	Phys	2	-1	0	-1	1	0	0	-1	-1	1	1	0	1	0	0	0	0	0	0	1	1	0	0	0	-1	0	0	0	1
P145	Math	1	-1	2	-3	1	0	0	1	2	2	0	1	2	0	0	1	1	1	2	2	1	2	-1	2	1	1	1	1	1
P145	Math	2	-2	1	2	0	-1	1	1	3	1	2	0	0	1	0	0	2	1	2	2	3	1	0	1	1	2	1	1	2
P145	Math	3	0	0	0	0	0	0	0	0	0	0	0	0	0	0	0	0	0	0	0	0	0	0	0	0	0	0	0	0
P146	Math	1	2	-2	2	2	2	2	-2	2	2	-2	2	2	-2	-2	2	-2	-2	-2	2	-2	-2	2	2	-2	-2	2	2	-2
P147	Math	1	0	3	2	2	1	2	2	1	1	-1	1	0	2	2	-2	1	1	3	2	2	2	0	1	-1	2	1	0	2
P147	Math	2	0	2	2	2	1	1	-1	2	1	2	0	-1	1	-1	-2	1	1	2	2	0	1	1	2	2	3	1	0	3
P147	Math	3	0	2	2	2	1	2	1	3	3	0	1	-2	1	2	3	1	2	2	3	2	2	-2	1	2	0	2	1	1
P148	Math	1	0	3	3	3	1	0	3	2	2	1	3	0	3	3	3	3	3	3	3	3	3	1	3	3	3	3	2	2
P148	Math	2	1	2	3	3	1	2	3	3	3	0	1	0	2	0	0	3	3	3	3	3	3	3	3	3	3	3	0	2
P148	Math	3	0	2	2	2	0	3	3	3	2	-1	2	-1	3	3	3	3	3	3	3	2	3	3	-3	3	3	3	2	3
P149	Math	1	1	1	2	2	0	0	0	3	1	0	0	0	0	0	0	0	0	0	1	0	0	0	0	1	1	1	0	0
P149	Math	2	2	-2	2	2	2	2	1	2	1	0	0	0	0	0	0	0	0	-3	3	-3	-3	2	2	0	0	1	0	0
P149	Math	3	1	3	3	3	-3	0	0	3	1	0	0	0	3	2	1	2	0	1	1	0	0	2	2	0	3	1	2	0
P150	Math	1	-1	1	2	2	3	2	3	2	2	2	2	0	2	3	0	2	2	3	3	3	1	-2	0	3	3	0	2	2
P150	Math	2	0	0	0	0	0	0	0	0	0	0	0	0	0	0	0	0	0	0	0	0	0	0	0	0	0	0	0	0
P151	Math	1	1	1	1	0	1	0	1	2	2	2	0	0	1	0	0	1	1	1	1	2	0	0	1	1	1	1	1	1
P151	Math	2	1	1	2	1	2	2	1	2	2	1	1	0	1	0	0	2	2	2	2	1	2	1	3	1	2	2	1	1
P151	Math	3	1	0	2	2	3	0	1	2	2	2	0	0	2	0	0	1	2	2	2	2	1	0	1	1	1	1	1	1

AttrakDiff RAW Data (All Participants)																														
ID	CLASS	ROUND	1	2	3	4	5	6	7	8	9	10	11	12	13	14	15	16	17	18	19	20	21	22	23	24	25	26	27	28
P152	Math	1	0	0	2	2	1	0	0	1	1	0	0	0	1	0	2	1	1	1	2	1	0	0	2	2	0	0	1	0
P152	Math	2	0	2	2	0	1	1	2	2	1	0	0	0	0	0	2	0	0	2	2	2	0	0	2	2	2	2	0	0
P152	Math	3	0	2	3	0	3	3	3	3	3	2	2	0	2	2	0	2	2	3	3	3	2	0	3	2	2	2	2	2
P153	Math	1	-2	1	3	0	-1	3	2	0	2	-2	3	3	3	0	0	2	2	3	3	-1	0	1	3	2	2	0	2	0
P153	Math	2	0	0	0	0	0	0	0	0	0	0	0	0	0	0	0	0	0	0	0	0	0	0	0	0	0	0	0	0
P153	Math	3	-3	3	3	-3	3	2	3	3	3	-1	0	0	0	0	0	0	0	0	0	0	0	0	0	0	0	-1	2	3
P154	Math	1	0	2	2	1	0	2	3	3	3	3	0	0	1	0	0	2	1	1	1	2	1	0	0	2	1	2	0	1
P154	Math	2	0	-1	1	1	1	1	-1	1	1	1	-1	0	0	-1	0	2	1	0	2	0	1	0	-1	0	1	1	0	0
P154	Math	3	0	0	0	0	0	0	0	0	0	0	0	0	0	0	0	0	0	0	0	0	0	0	0	0	0	0	0	0
P155	Math	1	3	-3	3	3	3	3	3	3	3	-3	3	0	3	-2	2	-2	0	-1	0	-1	0	0	0	-1	0	1	0	0
P155	Math	2	-1	3	3	0	0	3	3	3	3	3	3	-2	3	1	1	3	3	3	3	1	3	0	3	1	3	3	3	3
P155	Math	3	0	2	3	0	1	3	3	3	3	3	2	-1	3	2	0	1	2	2	3	1	2	0	3	2	3	2	3	2
P156	Math	1	-2	2	-2	-2	2	2	2	2	2	2	0	2	2	2	0	2	2	3	3	1	2	0	3	3	3	2	0	2
P156	Math	2	-3	3	3	3	0	3	3	3	3	-1	3	-3	3	2	3	3	3	3	3	3	2	2	3	2	3	3	1	3
P156	Math	3	0	2	3	3	2	3	3	3	3	-1	2	0	3	3	3	3	3	3	3	2	2	3	3	2	3	1	2	3
P157	Math	1	-1	1	-3	-3	-2	1	0	0	1	-2	-1	-1	1	2	1	1	1	1	1	0	0	1	1	-2	2	0	-1	3
P157	Math	2	-3	1	-2	0	-3	3	-3	3	-3	-3	1	-3	1	3	1	2	-3	-1	0	-2	-3	1	-2	-3	2	-1	-3	0
P157	Math	3	-3	0	-2	0	-3	3	-3	3	-2	0	0	0	0	0	0	-1	-2	-1	0	-3	-1	1	1	-3	3	1	0	0
P158	Mec Eng	1	2	1	2	-1	3	-1	0	2	2	1	1	1	2	2	-2	3	2	1	2	2	1	2	2	1	-1	1	1	1
P158	Mec Eng	2	0	0	0	0	0	0	0	0	0	0	0	0	0	0	0	0	0	0	0	0	0	0	0	0	0	0	0	0
P158	Mec Eng	3	0	0	0	0	0	0	0	0	0	0	0	0	0	0	0	0	0	0	0	0	0	0	0	0	0	0	0	0
P159	Mec Eng	1	0	1	-3	-2	0	-2	3	3	2	0	2	2	2	1	1	3	2	2	3	2	1	-1	2	1	2	-1	2	2
P159	Mec Eng	2	-1	0	1	2	1	2	2	-2	1	-1	2	0	2	1	1	3	2	1	2	1	2	0	1	1	3	0	1	1
P159	Mec Eng	3	-1	0	1	2	0	1	2	1	0	0	2	0	2	0	1	3	0	1	1	0	0	0	1	0	1	-1	2	0
P160	Mec Eng	1	-3	2	3	0	2	2	2	2	3	3	1	-1	2	2	1	3	1	2	2	3	1	0	2	2	3	2	1	2
P160	Mec Eng	2	-2	2	1	-1	0	1	1	2	2	2	1	0	1	3	2	2	3	1	2	1	0	0	0	0	1	1	0	1
P160	Mec Eng	3	-2	1	2	-2	-1	1	2	1	2	0	0	0	1	1	2	3	2	1	2	0	0	1	2	0	2	2	1	2
P161	Mec Eng	1	0	2	3	3	1	2	1	3	2	2	2	-2	2	3	2	3	2	3	3	3	2	2	2	3	3	3	3	2

AttrakDiff RAW Data (All Participants)																														
ID	CLASS	ROUND	1	2	3	4	5	6	7	8	9	10	11	12	13	14	15	16	17	18	19	20	21	22	23	24	25	26	27	28
P161	Mec Eng	2	2	2	3	3	3	2	2	3	3	1	3	1	2	3	2	2	3	3	3	2	2	2	2	2	2	3	3	2
P161	Mec Eng	3	2	2	3	3	2	2	2	3	3	2	2	3	2	2	3	2	3	3	3	3	3	2	3	3	2	2	2	2
P162	Mec Eng	1	-1	1	2	-2	0	3	3	2	1	2	3	-1	3	2	-2	2	1	0	2	2	1	-2	2	2	3	3	3	2
P162	Mec Eng	2	-1	3	3	2	1	2	2	2	2	2	2	-3	3	3	2	3	2	2	3	1	1	1	2	3	3	3	3	3
P162	Mec Eng	3	-2	2	2	3	2	3	3	2	2	2	2	-3	3	3	2	3	3	3	2	2	0	1	2	2	3	2	3	2
P163	Mec Eng	1	-1	1	2	3	1	2	2	2	3	2	2	2	2	3	2	2	2	3	3	2	3	2	3	1	1	2	2	2
P163	Mec Eng	2	-1	2	2	2	2	1	2	2	2	0	2	0	3	2	3	2	2	2	2	3	2	2	3	2	2	2	3	2
P163	Mec Eng	3	-1	1	3	3	0	1	2	2	2	2	2	0	3	2	1	2	2	3	2	2	1	2	3	2	2	2	2	2
P164	Mec Eng	1	2	2	2	2	-1	1	2	2	3	0	2	-1	2	1	-1	3	2	3	3	0	2	0	-3	3	1	3	2	2
P164	Mec Eng	2	-1	1	2	-2	-2	1	3	2	2	0	2	-1	1	0	-2	0	2	1	2	-2	1	2	-1	1	2	-1	2	2
P164	Mec Eng	3	-1	-1	2	1	1	1	2	2	2	1	0	1	1	1	0	2	2	2	2	0	1	1	2	1	1	2	2	1
P165	Mec Eng	1	-1	-1	1	0	0	3	0	1	1	0	1	0	2	0	-1	0	0	1	2	0	0	0	3	0	2	0	-1	0
P165	Mec Eng	2	0	0	0	0	0	3	-3	1	0	-1	0	1	0	1	1	0	0	0	1	1	1	-1	-1	1	1	0	-1	2
P165	Mec Eng	3	-3	3	-3	-3	-3	-3	3	-3	-3	3	-3	-3	-3	-3	-3	-3	-3	-3	-3	-3	-3	3	-3	-3	-3	-3	-3	-3
P166	Mec Eng	1	0	1	-1	1	1	0	1	1	1	0	0	-1	1	0	0	0	1	2	1	1	1	0	1	2	1	1	0	0
P166	Mec Eng	2	0	1	2	2	1	1	1	2	2	1	1	-3	2	1	0	1	1	3	2	2	2	0	1	-2	3	1	2	1
P166	Mec Eng	3	0	0	1	1	1	1	-1	3	2	-1	0	0	1	1	0	1	0	1	0	1	1	0	1	1	3	1	0	0
P167	Mec Eng	1	-2	3	3	2	2	2	3	3	3	2	2	1	2	2	2	3	3	3	3	3	1	-1	2	2	2	2	3	2
P167	Mec Eng	2	-2	3	3	3	1	2	2	3	3	2	2	0	2	2	1	3	3	3	3	3	2	-2	2	2	2	2	2	2
P167	Mec Eng	3	-2	2	3	2	1	2	2	2	2	2	3	1	2	3	2	3	2	3	3	2	2	1	2	2	2	2	2	2
P168	Mec Eng	1	0	2	0	0	1	0	-1	-1	2	0	-1	0	0	0	2	1	1	2	1	-1	0	0	1	-1	2	2	-1	1
P168	Mec Eng	2	0	0	1	2	0	2	1	0	1	-1	0	-1	1	0	0	2	2	3	1	0	1	1	1	1	2	1	2	2
P168	Mec Eng	3	0	1	1	0	0	2	2	0	2	1	3	0	2	2	-2	2	2	2	2	0	0	1	0	2	1	2	2	1
P169	Mec Eng	1	0	0	3	0	-2	-1	0	1	1	-2	1	0	0	0	-3	0	0	0	0	0	0	0	0	0	0	0	0	0
P169	Mec Eng	2	2	2	2	0	2	-3	3	3	3	-3	3	-2	2	-2	0	2	2	2	2	1	2	-2	3	2	3	3	3	2
P169	Mec Eng	3	3	3	3	2	3	0	3	3	3	-1	3	0	3	0	3	3	1	3	3	3	0	-2	-2	2	2	3	2	2
P170	Mec Eng	1	0	3	3	3	1	3	3	3	3	1	3	-1	3	3	3	3	3	3	3	3	3	-3	3	3	3	3	3	3
P170	Mec Eng	2	2	2	2	2	0	2	2	3	2	0	2	0	2	2	0	3	2	2	2	2	0	-1	2	2	2	2	2	2

AttrakDiff RAW Data (All Participants)																														
ID	CLASS	ROUND	1	2	3	4	5	6	7	8	9	10	11	12	13	14	15	16	17	18	19	20	21	22	23	24	25	26	27	28
P170	Mec Eng	3	-3	3	3	3	-1	3	3	3	3	2	3	-2	3	3	3	3	3	3	3	3	3	0	3	3	3	3	3	3
P171	Mec Eng	1	0	2	1	1	0	2	3	-3	2	1	3	-3	0	0	1	2	3	3	2	3	0	3	2	3	3	0	3	3
P171	Mec Eng	2	0	0	1	1	0	1	1	1	0	0	0	0	1	0	0	2	2	3	1	1	0	0	1	1	1	0	1	1
P171	Mec Eng	3	0	0	0	0	0	0	0	0	0	0	0	0	0	0	0	0	0	0	0	0	0	0	0	0	0	0	0	0
P172	Mec Eng	1	-1	2	2	2	0	-2	1	2	2	0	1	-2	3	0	0	1	2	3	2	0	2	0	2	0	1	0	2	1
P172	Mec Eng	2	0	1	2	2	0	0	0	1	-2	0	0	-2	0	2	0	1	1	3	3	0	0	0	0	1	1	1	3	0
P172	Mec Eng	3	-1	0	0	0	0	0	0	0	0	0	0	0	0	0	0	0	0	0	0	0	0	0	0	0	0	0	0	0
P173	Mec Eng	1	-1	2	2	0	2	1	2	2	3	2	1	2	1	2	0	1	1	2	2	2	1	0	1	1	-1	2	2	1
P173	Mec Eng	2	-2	2	2	1	0	1	2	2	2	2	1	0	1	1	1	1	1	1	2	2	1	0	1	1	-1	1	1	-1
P173	Mec Eng	3	0	0	-1	0	0	-1	0	0	-1	-1	0	0	-2	0	0	-1	-1	1	-1	0	-1	1	1	1	-1	-1	0	-1
P174	Mec Eng	1	0	1	2	1	-2	2	0	2	2	0	2	1	2	0	1	3	2	2	3	2	0	2	2	0	2	2	2	1
P174	Mec Eng	2	-2	2	0	0	1	2	2	2	2	2	3	1	2	-2	2	2	2	1	2	1	0	0	1	2	0	2	0	2
P174	Mec Eng	3	0	0	0	0	0	1	2	2	1	1	2	0	2	0	1	2	2	1	2	2	1	0	0	1	0	0	-2	2
P175	Mec Eng	1	-3	0	0	1	-1	3	3	1	-3	0	1	-1	1	1	0	2	-2	3	0	-1	0	-1	3	1	3	2	1	2
P175	Mec Eng	2	-3	2	0	0	0	3	3	3	0	3	1	-3	3	-1	3	3	0	3	3	3	0	-3	2	3	3	1	2	1
P175	Mec Eng	3	-2	3	2	3	0	2	3	3	0	3	2	0	3	0	0	3	3	3	3	3	3	0	3	1	3	2	0	1
P176	Mec Eng	1	1	3	2	2	1	2	2	2	2	2	2	0	2	2	1	2	2	2	2	3	2	0	2	1	2	2	0	2
P176	Mec Eng	2	0	0	0	0	0	0	0	0	0	0	0	0	0	0	0	0	0	0	0	0	0	0	0	0	0	0	0	0
P177	Math	1	1	2	1	1	1	2	2	2	2	-1	3	0	2	2	2	2	2	2	1	1	1	2	2	2	2	1	2	0
P177	Math	2	-3	2	-3	-3	1	-3	3	-3	-3	3	-1	1	-2	-2	2	-2	-2	-2	2	-2	-1	2	2	-2	-1	2	2	-2
P177	Math	3	3	-3	3	3	3	3	-3	3	3	-1	1	1	0	3	2	0	0	-2	1	-2	-2	1	2	-2	-2	2	2	-2
P178	Math	1	0	1	0	3	0	3	1	0	0	0	2	1	3	0	0	0	0	2	0	1	0	0	0	-1	3	1	-1	1
P178	Math	2	-2	0	0	3	-2	3	3	2	1	0	3	2	3	2	0	1	2	3	2	3	0	3	3	2	3	3	3	3
P178	Math	3	0	0	0	0	0	0	0	0	0	0	0	0	0	0	0	0	0	0	0	0	0	0	0	0	0	0	0	0
P179	Math	1	0	1	1	0	2	1	1	1	2	0	2	0	-1	0	0	0	1	2	1	-2	0	0	1	0	1	0	0	0
P179	Math	2	0	0	-1	0	-1	0	1	2	2	2	2	0	0	0	0	2	2	2	2	-1	0	0	0	0	2	-2	0	0
P180	Phys	1	-3	0	-1	0	-3	3	2	-2	-2	0	0	0	2	0	0	0	-1	1	0	-1	-1	0	1	-1	2	0	2	0
P180	Phys	2	-1	1	1	0	-2	1	2	1	2	0	2	0	1	0	2	3	3	3	2	3	1	0	1	2	2	2	2	1

AttrakDiff RAW Data (All Participants)																														
ID	CLASS	ROUND	1	2	3	4	5	6	7	8	9	10	11	12	13	14	15	16	17	18	19	20	21	22	23	24	25	26	27	28
P181	Mat Eng	1	-1	-2	-1	2	-2	1	-1	1	-1	-1	0	-1	2	0	-2	2	-2	1	1	-2	0	1	1	-2	2	-2	-1	1
P182	Mat Eng	1	-3	1	1	-1	0	3	-3	3	3	3	-3	-1	1	2	0	2	0	3	3	2	-2	-2	-2	0	0	1	3	2
P182	Mat Eng	2	0	0	0	0	0	0	0	0	0	0	0	0	0	0	0	0	0	0	0	0	0	0	0	0	0	0	0	0
P183	Mat Eng	1	-1	1	2	-2	2	2	2	2	2	2	2	2	2	2	2	2	1	2	2	2	2	2	2	1	2	2	2	2
P183	Mat Eng	2	-2	-1	1	1	2	2	1	1	1	1	1	1	1	1	1	1	1	1	2	-1	1	1	1	1	1	2	1	1
P183	Mat Eng	3	-1	-1	-1	1	-2	1	-2	-1	-2	-2	-1	-1	-1	0	-1	-1	-1	1	-1	-2	0	0	0	-2	0	-1	1	0
P184	Phys	1	0	2	2	3	3	3	3	3	3	-1	1	0	3	3	0	3	3	3	3	3	3	3	3	3	3	3	2	2
P185	Phys	1	-2	1	2	0	0	0	2	-2	-1	0	2	0	1	0	0	0	0	3	2	1	0	0	2	0	1	1	1	1
P186	Phys	1	0	2	-1	1	-1	0	1	-2	1	-2	1	-2	1	2	0	1	1	2	0	-1	0	0	1	0	1	1	-1	1
P187	Math	1	0	0	2	1	-2	2	3	-1	0	-1	1	0	2	0	0	2	1	1	2	-2	0	0	0	-1	2	0	0	0
P188	Math	1	-1	0	2	2	2	2	2	3	3	3	2	-2	3	3	3	3	3	3	3	3	2	-2	3	2	2	2	2	2
P188	Math	2	-2	2	2	3	2	2	2	2	2	2	2	-1	2	2	1	2	2	2	3	3	1	0	2	1	1	1	2	2
P189	Mat Eng	1	-1	1	1	0	1	1	0	1	1	0	1	1	0	1	1	1	0	1	1	1	0	0	1	0	1	0	-1	1
P189	Mat Eng	2	-1	2	0	1	0	1	1	1	1	0	1	1	0	1	1	0	0	0	1	0	0	-1	-1	1	1	0	1	0
P189	Mat Eng	3	-1	1	0	1	0	1	1	1	1	1	1	1	0	-1	1	1	0	1	1	-1	0	-1	1	-1	1	1	0	2
P190	Phys	1	1	1	3	0	-2	3	3	1	2	0	2	-3	1	1	0	3	1	0	1	0	1	2	-2	1	1	-1	-1	1
P190	Phys	2	-3	-3	0	-3	-3	3	3	0	3	0	3	0	3	0	0	3	3	3	3	-3	0	0	3	0	3	3	3	3
P191	Phys	1	-1	1	2	-2	-1	2	2	2	0	2	2	-2	2	2	2	2	3	3	2	1	2	2	1	1	2	3	1	1
P191	Phys	2	0	0	0	0	0	0	0	0	0	0	0	0	0	0	0	0	0	0	0	0	0	0	0	0	0	0	0	0
P191	Phys	3	0	0	0	0	0	0	0	0	0	0	0	0	0	0	0	0	0	0	0	0	0	0	0	0	0	0	0	0
P192	Mat Eng	1	-3	2	3	3	3	2	2	2	2	1	2	-1	1	2	-2	2	2	3	3	2	2	2	2	2	1	2	1	2
P193	Mec Eng	1	-2	2	2	2	-1	3	2	3	3	1	2	-3	2	-1	2	2	1	2	1	1	0	2	2	2	3	3	3	-2
P193	Mec Eng	2	-3	1	3	3	-2	1	0	2	3	3	2	-2	3	3	3	3	3	3	3	3	3	-3	3	3	2	3	3	3
P193	Mec Eng	3	0	0	0	0	0	0	0	0	0	0	0	-2	2	2	-2	2	3	3	-3	3	3	-3	-3	2	1	-1	-1	1
P194	Mat Eng	1	0	0	0	0	0	0	0	0	0	0	0	0	0	0	0	0	0	0	0	0	0	0	0	0	0	0	0	0
P195	Mat Eng	1	2	2	2	2	-1	2	2	3	3	0	3	0	3	3	-1	3	3	3	3	3	3	0	3	3	3	3	3	3
P195	Mat Eng	2	3	3	3	3	1	0	3	3	3	-1	1	0	3	3	-1	3	3	3	3	3	0	3	3	3	3	0	3	3
P195	Mat Eng	3	-3	3	3	3	-2	3	3	0	3	-2	3	-1	3	2	-3	3	3	3	3	0	0	2	1	-1	-1	2	3	-2

3. Longitudinal Data

In this section we present only the data from the participants who participated in all 3 rounds of the study. We also present, for each participant, the longest sequence of invariant numbers ("Long String" column). Participants whose Long String was equal or higher than the cutoff value of 7 were analyzed and classified as careless respondents or not ("Careless?" column), according to the criteria defined on the paper. Additionally, we included a new line with the order of each pair of adjectives (the red and green blocks above the item's number), which helped us to identify whether the participant was careless or not in his/her responses. As both UEQ and AttrakDiff have some reversed pairs of adjectives (positive on the left and negative on the right), we analyzed whether the invariant answers occurred in the reversals. Participants with such invariant answers were excluded. We highlighted in bold the items which the responses did not remain consistent when the items were reversed. Details of the analysis process are presented in the paper.

UEQ Data (Only those who participated in all 3 rounds)

ID	CLASS	ROUND	1	2	3	4	5	6	7	8	9	10	11	12	13	14	15	16	17	18	19	20	21	22	23	24	25	26	Long String	Careless?
P3	Phys	1	3	3	3	3	3	3	3	-1	2	3	3	3	3	3	3	3	3	3	3	3	3	3	3	3	3	3	**4**	
P3	Phys	2	3	3	3	2	3	2	3	0	3	3	3	3	3	3	3	3	3	3	3	3	2	3	3	3	3	3	**4**	
P3	Phys	3	2	2	2	2	2	2	2	-1	1	2	2	2	0	1	2	2	2	1	2	2	2	2	2	2	2	2	**3**	
P5	Phys	1	2	-2	0	-3	0	-1	-1	0	0	-1	-3	-2	-3	0	2	0	0	-2	0	0	-2	0	0	-1	-1	2	**2**	
P5	Phys	2	1	0	-2	-3	1	1	0	-2	0	0	-1	0	-3	3	1	0	0	-3	0	1	-2	0	1	0	1	3	**2**	
P5	Phys	3	0	-2	0	-3	-1	-2	0	-3	0	1	-3	-3	-3	1	2	0	0	-3	0	0	-2	0	0	-1	0	0	**2**	
P6	Math	1	2	-1	0	-1	1	1	1	1	2	1	1	2	1	1	1	1	2	2	2	1	2	1	2	2	2	1	**4**	
P6	Math	2	0	0	0	0	0	0	0	0	0	0	0	0	0	0	0	0	0	0	0	0	0	0	0	0	0	0	**26**	Yes
P6	Math	3	-2	-2	1	-3	0	-1	-2	-3	-1	0	-2	0	-3	-2	0	-2	-1	-3	-1	0	-2	0	0	0	-1	0	**3**	
P8	Math	1	2	3	3	3	2	1	2	1	3	-2	3	3	3	3	3	2	3	2	3	2	3	3	3	2	2	2	**3**	
P8	Math	2	0	0	0	0	0	0	0	0	0	0	0	0	0	0	0	0	0	0	0	0	0	0	0	0	0	3	**25**	Yes
P8	Math	3	1	1	2	2	-3	1	1	-1	2	1	2	3	2	3	3	3	3	1	2	2	2	2	3	1	2	2	**3**	
P9	Math	1	0	1	-1	0	2	1	1	1	-3	-3	1	3	-2	0	0	2	2	2	3	3	0	3	3	3	0	0	**3**	
P9	Math	2	0	0	0	0	0	0	0	0	0	0	0	0	0	0	0	0	0	0	0	0	0	0	0	0	0	0	**26**	Yes
P9	Math	3	0	0	0	0	0	0	0	0	0	0	0	0	0	0	0	0	0	0	0	0	0	0	0	0	0	0	**26**	Yes
P10	Math	1	2	2	2	0	3	2	2	1	1	0	-2	3	-2	1	1	2	1	3	2	3	1	2	2	2	2	0	**3**	
P10	Math	2	2	1	2	1	3	1	3	2	2	2	-2	2	1	2	1	1	2	3	2	3	2	2	2	2	2	0	**4**	
P10	Math	3	1	1	0	0	2	0	1	0	1	0	-2	2	-2	1	1	1	-1	1	0	1	0	0	2	0	0	0	**4**	
P12	Mec Eng	1	0	-1	1	-2	1	2	0	-2	0	0	2	1	-1	1	2	2	-1	0	-1	2	-2	-1	-1	-1	-1	1	**4**	
P12	Mec Eng	2	2	1	1	2	2	0	2	0	2	2	2	2	0	0	2	1	2	1	0	1	0	1	0	1	2	-1	**2**	

UEQ Data (Only those who participated in all 3 rounds)																														
ID	CLASS	ROUND	1	2	3	4	5	6	7	8	9	10	11	12	13	14	15	16	17	18	19	20	21	22	23	24	25	26	Long String	Careless?
P12	Mec Eng	3	0	0	1	1	1	0	1	-1	1	2	1	0	0	0	1	1	0	0	1	1	0	1	1	1	1	1	3	
P15	Math	1	3	2	-2	0	0	1	2	2	-1	-1	1	2	-1	1	2	2	1	0	-1	1	1	1	1	2	2	-2	3	
P15	Math	2	1	1	1	0	1	0	1	0	1	-1	1	1	1	0	1	1	1	0	1	1	0	1	1	1	1	1	3	
P15	Math	3	3	3	1	0	-1	-1	1	-1	-1	-1	1	1	1	1	1	1	-1	0	0	1	-1	1	0	0	-1	1	5	
P16	Math	1	0	0	0	0	0	0	0	0	0	0	0	0	0	0	0	0	0	0	0	0	0	0	0	0	0	0	26	Yes
P16	Math	2	3	3	-3	-3	-3	3	3	3	-3	-3	3	-3	3	3	3	3	-3	-3	-3	3	-3	3	-3	-3	-3	3	26	Yes
P16	Math	3	3	3	-3	-3	-3	3	3	3	-3	-3	3	-3	3	3	3	3	-3	-3	-3	3	-3	3	-3	-3	-3	3	26	Yes
P17	Mat Eng	1	-1	-1	2	0	0	-1	0	0	2	2	-2	1	0	1	2	0	2	1	0	1	-1	2	2	2	1	2	3	
P17	Mat Eng	2	-1	-1	2	0	1	-1	0	0	2	2	0	0	1	0	2	0	1	0	1	1	-2	2	2	2	0	2	2	
P17	Mat Eng	3	-1	0	-1	0	1	-1	1	1	2	1	1	1	-1	0	1	0	1	-1	0	1	-1	1	1	1	0	1	3	
P18	Mat Eng	1	2	2	0	2	2	0	2	0	2	3	2	2	1	2	2	2	2	2	2	2	0	1	2	1	2	2	3	
P18	Mat Eng	2	0	0	0	0	0	0	0	0	0	0	0	0	0	0	0	0	0	0	0	0	0	0	0	0	0	0	26	Yes
P18	Mat Eng	3	-3	-3	2	1	2	-1	-1	0	0	0	0	0	0	0	0	0	0	0	0	0	0	0	0	0	0	0	19	Yes
P19	Mat Eng	1	3	1	-3	0	3	3	3	0	3	3	3	3	-1	3	3	3	3	3	3	3	2	3	3	3	3	3	3	
P19	Mat Eng	2	3	2	3	0	3	2	3	0	2	3	3	3	-1	3	3	1	3	3	2	2	0	3	3	3	3	3	3	
P19	Mat Eng	3	-2	-2	3	-3	3	-2	2	-3	0	3	3	0	-3	0	3	-3	3	0	0	3	-3	3	3	3	0	3	3	
P20	Mat Eng	1	1	1	-1	-1	-1	1	2	0	0	-1	1	-1	0	1	1	1	2	2	1	1	1	1	2	1	1	1	6	
P20	Mat Eng	2	0	0	0	0	0	0	0	0	0	0	1	-1	1	1	0	0	0	0	0	0	1	0	0	0	1	0	10	Yes
P20	Mat Eng	3	0	0	-1	-1	2	-1	1	1	0	1	0	-1	1	0	-1	-1	1	1	1	-1	1	-1	1	0	0	1	9	Yes
P22	Mat Eng	1	-3	-2	3	2	2	-2	-2	-2	2	2	-1	1	-2	-2	-2	-3	2	2	2	-2	3	-2	3	1	2	-3	7	Yes
P22	Mat Eng	2	2	0	-2	0	3	2	1	0	0	0	0	-1	-1	2	2	2	3	1	0	0	3	0	0	3	3	-3	4	
P22	Mat Eng	3	-1	-2	-3	1	-2	-1	-1	3	-1	2	-3	2	-1	0	2	2	3	-1	3	2	-1	-1	3	-1	-1	1	3	
P23	Mat Eng	1	1	1	1	2	2	1	1	-2	2	1	2	1	2	1	1	1	2	1	2	2	2	1	2	1	2	1	3	
P23	Mat Eng	2	1	2	-2	-1	0	2	1	0	-1	-1	2	0	1	2	0	1	-1	-1	0	0	-1	1	0	-1	0	2	3	
P23	Mat Eng	3	2	2	-2	-2	-2	1	0	0	-1	0	0	0	1	0	1	1	0	0	-1	0	0	1	-1	0	-1	1	5	
P24	Mat Eng	1	2	2	3	1	3	2	2	2	2	3	2	3	-1	2	2	2	2	2	1	3	0	3	2	2	2	2	3	
P24	Mat Eng	2	0	-1	1	-2	1	-1	0	-2	2	1	-2	0	-3	0	1	-1	0	-1	-1	0	-2	-1	0	-2	-3	2	2	
P24	Mat Eng	3	0	1	3	-2	3	-1	2	-2	3	2	1	3	-1	2	3	2	3	2	-1	2	-2	3	1	2	2	-3	2	

UEQ Data (Only those who participated in all 3 rounds)																														
ID	CLASS	ROUND	1	2	3	4	5	6	7	8	9	10	11	12	13	14	15	16	17	18	19	20	21	22	23	24	25	26	Long String	Careless?
P25	Mat Eng	1	1	1	1	2	2	0	1	2	1	1	1	1	2	1	1	1	1	2	2	2	1	1	1	2	1	0	**3**	
P25	Mat Eng	2	-1	0	-1	1	0	0	1	0	-1	0	-1	1	1	1	1	0	0	2	1	2	1	1	1	1	1	1	**3**	
P25	Mat Eng	3	-1	0	1	1	1	1	1	1	0	1	1	1	0	1	0	0	0	1	1	1	1	1	1	1	1	1	**3**	
P28	Mat Eng	1	2	2	1	1	2	1	2	-2	-1	3	3	3	0	2	3	2	2	2	2	3	-1	2	1	2	2	3	**3**	
P28	Mat Eng	2	-1	-1	-1	1	3	-1	1	0	1	1	0	1	0	1	1	2	1	-1	1	1	-1	-1	1	0	1	2	**2**	
P28	Mat Eng	3	1	1	-1	1	1	-1	1	1	1	3	2	3	1	1	3	2	1	1	2	1	1	1	2	1	1	3	**3**	
P30	Mat Eng	1	3	3	0	-3	0	1	1	0	-1	-1	1	3	-1	3	3	3	3	3	3	3	0	3	3	3	0	-3	**3**	
P30	Mat Eng	2	0	-2	-3	-2	-2	1	3	3	3	3	3	3	3	3	1	3	3	3	0	3	-2	3	3	-1	3	3	**2**	
P30	Mat Eng	3	1	3	-1	-3	-1	1	2	1	2	0	0	-1	2	0	3	0	3	-1	2	1	-3	-2	1	0	-1	3	**2**	
P33	Mat Eng	1	0	0	0	0	0	0	0	0	0	0	0	0	0	0	0	0	0	0	0	0	0	0	0	0	0	0	**26**	Yes
P33	Mat Eng	2	-1	-1	1	1	1	-1	-1	-1	1	1	-1	1	-1	-1	-1	-1	1	1	1	-1	1	-1	1	1	1	-1	**26**	Yes
P33	Mat Eng	3	0	0	0	0	0	0	0	**-2**	**2**	**2**	**-2**	-3	-3	-3	-3	-3	0	0	1	1	0	0	2	-1	-3	0	**7**	Yes
P40	Phys	1	2	2	-2	-2	1	0	2	0	2	2	2	2	1	2	1	2	1	2	2	2	2	2	2	2	2	2	**4**	
P40	Phys	2	3	3	3	3	3	0	3	1	3	3	3	3	3	3	3	3	3	3	3	3	3	3	3	3	3	3	**4**	
P40	Phys	3	2	2	2	2	2	0	2	1	2	2	3	3	2	2	2	2	2	2	2	2	2	2	2	2	2	2	**4**	
P44	Phys	1	1	1	0	0	0	-1	0	2	-2	0	0	0	1	1	0	0	1	-1	1	0	-1	2	0	0	1	0	**3**	
P44	Phys	2	-3	-3	0	0	0	0	-2	-2	-2	0	-2	-2	-2	-2	-1	-2	-1	-3	0	-1	-1	-1	-1	0	-3	0	**4**	
P44	Phys	3	-1	0	1	1	0	0	0	-2	0	0	-1	-1	0	-1	0	-1	-2	-3	0	0	-2	0	-1	1	1	0	**3**	
P45	Phys	1	2	2	2	1	2	0	1	0	1	1	1	1	2	2	1	1	0	1	0	2	2	2	1	1	3	-3	**2**	
P45	Phys	2	0	0	0	0	0	0	0	0	0	0	0	0	0	0	0	0	0	0	0	0	0	0	0	0	0	0	**26**	Yes
P45	Phys	3	1	1	1	2	2	0	1	0	0	1	0	1	1	1	0	1	1	2	1	2	2	2	2	2	2	-2	**4**	
P47	Phys	1	2	2	2	1	2	2	2	1	1	2	2	2	1	2	2	2	2	2	2	2	1	2	2	2	2	2	**3**	
P47	Phys	2	2	2	2	2	1	1	2	0	-1	2	2	2	1	2	2	1	2	3	2	2	2	2	2	2	2	1	**3**	
P47	Phys	3	2	1	1	1	2	1	1	2	2	2	1	2	0	2	2	2	2	0	1	1	1	2	2	2	1	1	**3**	
P54	Math	1	3	2	3	2	1	1	2	0	1	2	2	3	1	2	3	2	2	3	2	2	2	3	2	2	1	1	**2**	
P54	Math	2	2	2	2	2	1	1	2	1	1	2	2	3	1	2	2	2	1	2	2	3	1	2	2	1	2	3	**3**	
P54	Math	3	2	3	2	1	2	0	2	0	2	3	1	2	0	2	2	2	3	2	1	2	1	2	3	2	3	2	**3**	
P55	Math	1	1	0	0	1	1	0	0	0	0	0	0	1	0	1	1	1	1	1	1	1	0	0	1	0	0	0	**6**	

UEQ Data (Only those who participated in all 3 rounds)

ID	CLASS	ROUND	1	2	3	4	5	6	7	8	9	10	11	12	13	14	15	16	17	18	19	20	21	22	23	24	25	26	Long String	Careless?
P55	Math	2	0	0	0	0	1	0	0	-1	0	0	0	0	0	0	0	0	0	1	0	0	0	0	1	0	0	0	**9**	No
P55	Math	3	1	1	2	0	1	2	1	-1	-1	2	0	2	-1	1	1	1	1	1	0	1	1	0	1	1	1	1	**3**	
P56	Math	1	2	1	3	1	2	2	1	-1	2	3	3	3	0	2	3	3	3	3	2	3	1	2	3	2	2	3	**2**	
P56	Math	2	1	0	3	1	2	2	2	1	2	3	1	2	-3	3	3	3	3	2	3	3	1	3	2	1	2	3	**3**	
P56	Math	3	2	2	3	2	3	3	3	0	2	3	3	3	2	2	3	3	3	3	1	3	3	3	3	2	3	3	**2**	
P57	Math	1	1	1	2	0	0	1	2	0	0	0	0	0	0	2	2	2	1	0	0	0	0	0	2	1	0	0	**6**	
P57	Math	2	3	2	-2	1	2	2	3	2	-1	1	2	2	1	2	2	2	2	3	2	2	2	2	2	2	2	2	**3**	
P57	Math	3	3	2	2	2	2	1	3	1	2	0	1	2	1	2	2	2	1	2	2	2	1	1	2	1	2	-1	**3**	
P60	Math	1	3	3	1	-3	0	1	1	-1	0	1	0	1	-1	1	2	1	1	1	1	1	1	1	2	2	2	-2	**4**	
P60	Math	2	3	-2	2	-3	2	2	2	-3	2	2	-2	2	-3	3	3	2	2	3	2	2	0	3	3	3	3	3	**4**	
P60	Math	3	1	-1	1	-3	0	1	1	-1	0	1	-3	2	-3	1	2	1	3	0	-2	-1	-3	-3	0	1	2	0	**2**	
P61	Math	1	0	2	2	1	2	2	1	1	1	2	2	2	2	2	1	1	2	2	1	2	2	2	3	2	2	-2	**3**	
P61	Math	2	2	2	2	1	2	1	1	2	2	2	1	2	-1	1	0	1	0	-1	-3	1	1	1	1	0	1	-1	**2**	
P61	Math	3	0	2	2	1	1	0	1	1	1	1	1	1	2	2	2	2	1	1	1	1	1	1	1	1	1	1	**4**	
P63	Math	1	3	3	3	2	3	3	3	3	2	3	2	3	2	3	3	3	3	3	3	3	3	3	3	3	3	-3	**4**	
P63	Math	2	0	0	0	0	0	0	0	0	0	0	0	0	0	0	0	0	0	0	0	0	0	0	0	0	0	0	**26**	Yes
P63	Math	3	2	2	2	3	3	3	3	-2	-2	0	0	1	-1	0	1	2	2	0	0	0	0	0	0	0	0	0	**9**	No
P64	Mec Eng	1	3	2	2	2	3	2	3	1	0	2	2	3	2	2	2	3	1	3	2	1	2	2	2	3	3	2	**3**	
P64	Mec Eng	2	1	2	1	1	2	0	1	0	2	2	3	2	2	1	2	3	3	2	2	2	2	3	3	2	2	2	**2**	
P64	Mec Eng	3	0	1	1	0	2	0	1	-1	1	2	3	2	1	1	2	2	2	1	1	1	1	1	2	2	1	1	**2**	
P65	Mec Eng	1	2	2	1	1	2	1	2	0	-1	1	0	1	0	1	1	2	1	0	1	2	1	0	1	2	2	1	**2**	
P65	Mec Eng	2	1	0	0	1	1	1	0	0	1	1	1	1	1	1	-1	0	1	1	1	1	1	1	1	1	1	1	**3**	
P65	Mec Eng	3	1	0	-1	-1	-1	1	1	1	-1	-1	1	-1	1	1	1	1	-1	-1	-1	1	-1	1	-1	-1	-1	1	**24**	Yes
P67	Mec Eng	1	1	1	1	0	2	0	1	1	0	1	1	2	0	2	1	2	0	1	1	2	0	2	-2	1	1	2	**2**	
P67	Mec Eng	2	1	2	2	2	2	0	2	1	0	2	2	2	2	1	2	2	0	1	0	1	1	2	2	0	0	2	**3**	
P67	Mec Eng	3	0	0	0	0	0	0	0	0	0	0	0	0	0	0	0	0	0	0	0	0	0	0	0	0	0	0	**26**	Yes
P68	Mec Eng	1	1	1	0	1	0	0	1	0	0	1	1	2	1	1	-1	1	-1	0	1	1	1	1	2	2	2	1	**3**	
P68	Mec Eng	2	-2	-2	2	1	2	-1	-1	0	1	0	-1	2	-3	-1	-2	-1	0	-1	-1	0	0	1	2	1	1	0	**3**	

UEQ Data (Only those who participated in all 3 rounds)

ID	CLASS	ROUND	1	2	3	4	5	6	7	8	9	10	11	12	13	14	15	16	17	18	19	20	21	22	23	24	25	26	Long String	Careless?
P68	Mec Eng	3	0	0	1	2	1	0	1	0	0	1	-1	0	1	1	0	1	-1	-1	0	1	0	1	1	0	0	-1	**3**	
P69	Mec Eng	1	-3	-3	-3	0	3	-3	-2	3	3	0	-3	2	-3	-3	2	-2	3	-3	-2	-2	-3	-3	-2	-2	-3	0	**2**	
P69	Mec Eng	2	-2	3	-2	-2	3	-2	-1	3	3	3	-2	-2	-3	-2	-2	-2	3	-1	2	3	3	-3	3	-2	-2	-3	**3**	
P69	Mec Eng	3	-1	-1	1	1	1	-1	-1	-1	1	1	0	0	0	0	0	0	0	0	0	0	0	0	0	0	0	0	**16**	Yes
P70	Mec Eng	1	3	2	2	2	3	2	2	-2	2	2	-2	3	2	3	3	3	0	2	2	3	2	3	0	3	2	3	**4**	
P70	Mec Eng	2	3	3	3	-3	1	3	3	0	3	3	3	3	-1	3	3	2	1	2	3	3	3	3	1	1	1	2	**3**	
P70	Mec Eng	3	0	0	0	0	0	0	0	0	0	0	0	0	0	0	0	0	0	0	0	0	0	0	0	0	0	0	**26**	Yes
P72	Mec Eng	1	1	1	2	0	0	-1	-2	-1	1	1	1	2	1	1	1	1	-1	1	1	1	1	1	-1	-1	1	1	**5**	
P72	Mec Eng	2	1	-1	1	1	1	1	1	1	1	1	1	2	2	1	2	2	1	1	2	2	-1	2	1	1	1	2	**4**	
P72	Mec Eng	3	-1	0	-1	-1	3	1	0	0	0	1	-2	1	-1	0	1	-1	0	0	0	1	-2	-1	1	1	-1	1	**3**	
P73	Mec Eng	1	0	0	0	0	0	0	0	0	**1**	**1**	**-1**	**1**	**-1**	1	0	0	1	0	0	**1**	**-1**	0	0	0	0	1	**8**	Yes
P73	Mec Eng	2	-1	-1	0	-2	0	-1	-1	0	0	0	-1	0	-2	0	0	-1	1	-2	0	0	-3	-1	0	0	-1	1	**3**	
P73	Mec Eng	3	-1	-1	0	-1	0	-1	-1	0	0	0	-1	1	-2	1	0	-1	0	-1	0	0	-1	0	0	0	0	0	**5**	
P74	Mec Eng	1	3	3	2	2	3	2	3	0	0	3	2	3	1	2	2	2	3	3	3	3	2	2	3	3	2	2	**3**	
P74	Mec Eng	2	2	2	1	2	2	1	2	-1	1	2	1	2	1	2	2	2	2	1	2	2	2	2	2	2	2	2	**3**	
P74	Mec Eng	3	1	1	1	1	2	2	2	0	0	3	2	3	1	1	3	2	2	2	2	2	2	2	2	2	2	3	**3**	
P75	Mec Eng	1	2	2	1	1	1	1	1	-1	1	1	1	2	1	1	1	1	1	2	1	1	1	1	1	1	1	-1	**4**	
P75	Mec Eng	2	1	2	2	2	2	1	2	1	2	2	1	2	1	1	0	2	1	1	2	2	1	2	2	2	2	0	**3**	
P75	Mec Eng	3	2	2	1	1	1	1	2	0	1	1	1	1	1	1	1	1	1	1	2	2	1	2	2	1	1	1	**4**	
P77	Mec Eng	1	2	2	2	3	3	0	2	-3	2	3	3	3	2	3	3	3	3	3	3	2	3	3	3	3	3	3	**3**	
P77	Mec Eng	2	2	3	2	2	3	2	3	2	2	-1	3	3	2	3	3	3	2	3	3	2	3	3	2	3	3	2	**3**	
P77	Mec Eng	3	0	3	2	2	3	1	2	-2	2	2	3	2	0	3	3	3	3	1	2	3	3	3	2	2	3	3	**3**	
P78	Mec Eng	1	2	2	2	1	3	2	3	1	1	3	1	3	0	2	3	2	2	2	2	2	2	2	3	3	3	3	**3**	
P78	Mec Eng	2	3	3	3	2	3	2	3	0	2	3	3	3	0	3	3	3	3	2	3	3	2	3	2	2	2	3	**3**	
P78	Mec Eng	3	3	3	3	3	3	3	3	0	3	3	3	2	0	0	3	3	2	3	3	2	3	3	2	2	2	-2	**4**	
P79	Mec Eng	1	2	2	2	3	1	2	3	1	2	-2	3	3	1	2	2	3	3	3	2	2	2	2	1	3	3	0	**2**	
P79	Mec Eng	2	3	3	-3	-3	-3	2	1	2	0	-3	2	1	2	0	1	2	-1	1	0	2	-1	2	-1	-2	-2	2	**5**	
P79	Mec Eng	3	3	3	-3	-3	-3	3	3	3	-3	-3	3	-3	3	3	3	3	-3	-3	-3	3	-3	3	-3	-3	-3	3	**26**	Yes

UEQ Data (Only those who participated in all 3 rounds)																														
**ID**	**CLASS**	**ROUND**	**1**	**2**	**3**	**4**	**5**	**6**	**7**	**8**	**9**	**10**	**11**	**12**	**13**	**14**	**15**	**16**	**17**	**18**	**19**	**20**	**21**	**22**	**23**	**24**	**25**	**26**	**Long String**	**Careless?**
P80	Mec Eng	1	1	1	1	1	2	0	2	0	1	2	-2	2	-1	-1	1	1	0	-1	0	1	1	1	-1	-2	0	0	**3**	
P80	Mec Eng	2	0	-1	2	0	0	0	0	0	1	1	0	1	1	0	0	0	0	0	0	1	-1	1	1	0	1	-1	**6**	
P80	Mec Eng	3	1	2	1	0	1	1	1	0	3	2	2	3	1	2	3	2	2	2	3	2	1	3	3	2	3	-2	**2**	
P81	Mec Eng	1	3	3	2	2	1	1	2	0	-1	0	2	2	1	1	0	2	-2	1	2	0	2	1	2	2	2	0	**3**	
P81	Mec Eng	2	2	2	1	3	1	0	2	1	1	1	2	2	2	2	1	1	1	2	2	1	1	0	1	1	1	1	**3**	
P81	Mec Eng	3	0	0	0	0	0	0	0	0	0	0	0	0	0	0	0	0	0	0	0	0	0	0	0	0	0	0	**26**	Yes
P82	Mec Eng	1	3	3	3	2	3	2	3	0	-1	3	2	2	1	1	3	3	2	3	0	2	2	2	2	2	2	2	**3**	
P82	Mec Eng	2	-2	0	0	2	2	1	1	0	-2	2	2	2	1	1	1	1	2	2	2	0	1	1	2	2	2	1	**4**	
P82	Mec Eng	3	-2	-2	3	**1**	**1**	**-1**	**-1**	**-1**	**1**	**1**	**-1**	**1**	**-1**	**-1**	0	0	0	0	0	0	0	0	0	0	0	0	**12**	Yes
P85	Mat Eng	1	3	0	3	0	2	2	3	0	2	2	2	3	0	3	3	2	2	3	2	2	0	2	2	2	1	3	**2**	
P85	Mat Eng	2	1	0	3	-1	2	1	3	0	1	3	0	3	-1	3	3	1	3	3	2	2	-1	3	2	3	1	3	**2**	
P85	Mat Eng	3	1	-1	2	-1	1	0	1	-1	1	3	1	2	-2	0	3	0	2	2	1	2	-1	0	2	1	0	2	**2**	
P86	Math	1	2	0	1	0	3	-2	3	-1	1	2	2	1	1	3	2	2	1	2	3	3	0	1	3	3	2	1	**2**	
P86	Math	2	1	0	-1	0	-1	0	-1	0	-1	0	0	-1	0	-1	0	1	-1	-1	0	0	0	1	0	0	0	0	**4**	
P86	Math	3	0	0	0	0	0	0	0	0	0	0	0	0	0	0	0	0	0	0	0	0	0	0	0	0	0	0	**26**	Yes
P92	Mat Eng	1	1	1	2	1	2	0	0	0	2	1	3	2	2	2	1	3	1	2	3	2	2	3	3	0	2	1	**3**	
P92	Mat Eng	2	0	1	1	2	1	0	1	1	-1	1	2	3	1	2	1	1	1	2	1	2	1	-2	2	1	-1	-1	**3**	
P92	Mat Eng	3	0	0	0	0	0	0	0	0	0	0	0	0	0	0	0	0	0	0	0	0	0	0	0	0	0	0	**26**	Yes
P93	Mec Eng	1	1	1	2	0	0	1	1	0	1	1	0	1	-1	1	1	1	0	1	0	1	0	1	1	1	1	0	**3**	
P93	Mec Eng	2	2	0	0	1	0	1	1	0	0	-1	0	0	2	2	1	1	0	0	3	1	0	2	2	1	2	0	**2**	
P93	Mec Eng	3	0	0	0	0	0	0	0	0	0	0	0	0	0	0	0	0	0	0	0	0	0	0	0	0	0	0	**26**	Yes
P94	Mec Eng	1	0	-1	-2	-3	1	-2	0	2	0	0	0	0	-1	0	0	0	0	0	0	0	-1	0	0	0	0	0	**7**	No
P94	Mec Eng	2	0	1	0	1	0	0	0	3	3	0	0	2	-1	0	0	0	2	-1	2	1	1	1	0	0	0	0	**4**	
P94	Mec Eng	3	-1	-1	0	-1	-2	-2	-1	1	-2	-1	0	1	1	-2	-1	0	0	-2	0	0	0	0	0	0	0	0	**8**	No
P95	Phys	1	1	0	1	2	1	-1	-1	0	2	0	1	0	1	0	-2	1	2	-1	-2	0	1	-3	1	2	-1	-1	**3**	
P95	Phys	2	1	1	1	1	3	0	2	2	2	3	1	2	0	1	1	1	0	1	2	3	0	-2	1	0	1	1	**3**	
P95	Phys	3	2	2	-2	-2	-2	2	2	2	-2	-2	2	-2	2	2	2	2	-2	-2	-2	2	-2	2	-2	-2	-2	2	**26**	Yes
P99	Mat Eng	1	2	2	3	2	2	2	3	0	2	3	3	3	2	3	3	3	3	3	2	3	3	3	3	2	2	3	**3**	

UEQ Data (Only those who participated in all 3 rounds)

ID	CLASS	ROUND	1	2	3	4	5	6	7	8	9	10	11	12	13	14	15	16	17	18	19	20	21	22	23	24	25	26	Long String	Careless?
P99	Mat Eng	2	2	2	3	2	0	1	2	0	2	2	3	3	3	2	2	2	2	2	3	3	3	3	3	3	3	2	**3**	
P99	Mat Eng	3	3	3	3	3	0	3	3	0	-1	3	3	3	3	3	3	0	3	3	3	3	3	3	3	3	3	3	**3**	
P100	Mat Eng	1	0	0	-2	0	0	-3	-1	0	-1	0	-1	1	-1	0	-1	1	2	2	0	0	-2	0	-2	-1	0	0	**3**	
P100	Mat Eng	2	0	0	0	0	0	0	0	0	0	0	0	0	0	0	0	0	0	0	0	0	0	0	0	0	0	0	**26**	Yes
P100	Mat Eng	3	0	-3	3	0	-1	-2	-2	-2	-1	0	0	0	-3	0	1	-2	-2	-3	0	0	0	0	0	-1	-3	2	**5**	
P101	Mec Eng	1	0	1	1	0	0	0	1	-1	1	-1	1	1	1	1	-1	1	1	1	1	1	1	1	1	1	1	1	**3**	
P101	Mec Eng	2	1	1	1	0	1	1	1	2	1	0	1	1	0	1	1	1	1	1	1	1	1	1	-1	1	1	1	**3**	
P101	Mec Eng	3	1	1	1	0	0	1	1	0	1	-1	1	1	0	1	-1	1	0	1	1	1	1	1	-1	-1	1	1	**3**	
P102	Mat Eng	1	1	0	0	0	0	1	0	1	1	-1	1	2	1	1	2	1	2	2	0	2	0	1	2	3	3	-2	**4**	
P102	Mat Eng	2	0	0	0	0	0	0	0	0	0	0	0	0	0	0	0	0	0	0	0	0	0	0	0	0	0	0	**26**	Yes
P102	Mat Eng	3	-3	-3	-3	-3	-3	-3	-3	-3	-3	-3	-3	-3	-3	-3	-3	-3	-3	-3	-3	-3	-3	-3	-3	-3	-3	-3	**4**	
P104	Mat Eng	1	0	0	2	2	2	0	2	0	2	0	2	2	0	2	0	2	0	2	0	0	0	3	3	3	2	2	**3**	
P104	Mat Eng	2	1	1	0	1	1	0	1	1	2	0	1	1	0	1	1	1	0	1	1	1	0	2	2	2	1	1	**3**	
P104	Mat Eng	3	1	1	1	1	1	0	1	1	1	0	1	1	0	1	1	1	1	0	0	0	0	0	0	0	0	0	**9**	No

AttrakDiff Data (Only those who participated in all 3 rounds)																																
**ID**	**CLASS**	**ROUND**	**1**	**2**	**3**	**4**	**5**	**6**	**7**	**8**	**9**	**10**	**11**	**12**	**13**	**14**	**15**	**16**	**17**	**18**	**19**	**20**	**21**	**22**	**23**	**24**	**25**	**26**	**27**	**28**	**Long String**	**Careless?**
P105	Phys	1	0	1	1	1	0	1	1	1	1	1	1	-2	0	0	0	1	2	2	2	1	0	0	1	2	1	1	0	0	**3**	
P105	Phys	2	0	0	0	0	1	2	0	2	2	-2	1	0	-2	-2	2	-1	-2	-2	1	-2	-2	1	1	-1	-2	1	2	-1	**4**	
P105	Phys	3	-1	1	2	2	2	1	1	1	1	-2	1	1	0	0	0	1	1	1	2	1	0	0	0	1	0	1	0	0	**3**	
P111	Math	1	-1	1	3	2	2	1	2	-2	-1	0	0	0	2	2	0	2	2	3	3	1	1	0	2	2	2	2	0	2	**3**	
P111	Math	2	-1	0	2	1	0	1	3	2	2	0	2	-1	2	2	-1	2	2	2	2	1	2	0	2	2	2	2	1	2	**3**	
P111	Math	3	-1	0	2	2	0	1	2	2	2	1	1	-1	2	2	0	2	2	1	2	2	2	0	2	2	2	2	0	2	**2**	
P116	Phys	1	0	0	0	0	0	0	0	0	0	-1	1	1	-1	-1	1	-1	-1	-1	1	-1	-1	1	1	-1	-1	1	1	-1	**19**	Yes
P116	Phys	2	0	0	0	0	1	1	0	0	0	0	0	1	2	0	0	0	0	0	0	0	1	0	1	0	0	0	0	0	**7**	No
P116	Phys	3	-3	3	3	-1	0	2	1	1	1	2	2	1	1	1	1	0	0	2	2	1	2	1	1	-1	0	0	0	0	**4**	
P117	Phys	1	0	0	-1	0	0	0	2	-2	0	3	1	1	2	0	0	2	2	2	1	2	0	0	1	0	0	0	0	0	**5**	
P117	Phys	2	0	0	0	0	0	0	0	0	0	0	0	0	0	0	0	0	0	0	0	0	0	0	0	0	0	0	0	0	**28**	Yes
P117	Phys	3	1	0	2	1	3	-1	2	1	3	3	2	3	3	2	0	2	2	1	3	2	2	0	2	2	0	2	1	2	**2**	
P121	Mat Eng	1	-1	2	2	2	1	1	2	2	3	2	1	1	2	2	3	2	2	2	3	2	1	1	2	1	2	2	1	2	**3**	
P121	Mat Eng	2	-1	0	1	1	1	0	1	1	1	1	1	1	1	1	1	2	1	1	2	0	0	1	1	1	2	0	0	1	**3**	
P121	Mat Eng	3	-1	1	2	2	1	1	2	1	1	1	1	1	2	2	2	2	2	2	2	2	1	1	1	2	2	2	1	1	**3**	
P122	Mat Eng	1	-2	3	3	-3	0	0	3	3	3	2	2	0	2	0	0	2	0	2	3	0	0	0	0	3	1	0	0	0	**4**	
P122	Mat Eng	2	0	1	0	1	0	0	0	1	1	0	1	0	1	0	0	1	1	1	1	1	-1	0	0	1	1	0	0	1	**3**	
P122	Mat Eng	3	0	1	1	-1	0	0	0	1	1	0	0	1	0	0	0	1	0	0	1	0	0	0	0	0	0	0	0	0	**9**	No
P124	Mat Eng	1	-1	1	2	2	2	2	0	2	2	-2	0	1	1	3	1	0	2	2	2	2	2	-1	2	2	2	2	2	2	**4**	
P124	Mat Eng	2	-1	1	0	1	-1	1	1	0	-1	-1	1	-1	1	-1	-3	1	1	1	1	-2	1	-1	-1	1	1	0	1	0	**5**	
P124	Mat Eng	3	3	1	0	-1	-1	0	1	0	0	0	3	-1	0	1	0	2	-1	-1	-1	0	0	-3	-1	1	1	1	1	1	**3**	
P125	Mat Eng	1	0	0	0	0	0	0	0	0	0	0	0	0	0	0	0	0	0	0	0	0	0	0	0	0	0	0	0	0	**28**	Yes
P125	Mat Eng	2	0	0	0	0	0	0	0	0	0	0	0	0	0	0	0	0	0	-1	-1	-1	1	1	-1	-1	1	1	0	0	**17**	Yes
P125	Mat Eng	3	0	0	0	0	0	0	0	0	0	0	0	0	0	0	0	0	0	0	0	0	0	0	0	0	0	0	0	0	**28**	Yes
P126	Mat Eng	1	1	1	0	-1	0	1	1	2	2	0	0	2	1	2	1	2	0	2	2	1	2	1	0	1	3	2	1	2	**2**	
P126	Mat Eng	2	-2	-1	0	1	-1	2	-1	-1	-1	2	1	2	0	1	-1	1	-1	1	-1	1	-2	1	1	0	2	1	1	1	**3**	
P126	Mat Eng	3	-1	0	1	0	-1	1	0	0	0	2	1	-1	2	1	-2	2	-1	2	1	0	-1	-1	-1	-1	3	-1	0	0	**3**	

AttrakDiff Data (Only those who participated in all 3 rounds)																															
ID	CLASS	ROUND	1	2	3	4	5	6	7	8	9	10	11	12	13	14	15	16	17	18	19	20	21	22	23	24	25	26	27	28	Long String	Careless?
P127	Mat Eng	1	-1	0	1	0	0	1	1	1	2	0	0	0	1	3	1	3	0	2	3	0	0	3	1	0	1	1	2	0	3	
P127	Mat Eng	2	-2	2	1	2	0	1	3	3	3	1	2	0	3	2	1	2	0	3	3	1	0	0	3	1	1	0	1	2	2	
P127	Mat Eng	3	0	1	1	0	0	0	2	2	1	0	1	0	1	1	1	1	0	1	2	0	0	0	1	1	1	1	1	1	3	
P129	Mat Eng	1	0	-2	2	3	3	2	2	2	0	0	3	-2	1	1	-2	1	2	1	2	-1	2	2	3	0	3	0	3	0	2	
P129	Mat Eng	2	-2	-2	-1	0	0	1	0	-1	-1	2	0	-2	-2	0	-1	-2	-2	2	-2	0	-1	-3	-2	0	2	-1	1	-2	2	
P129	Mat Eng	3	3	-3	3	3	3	3	-3	3	3	-3	3	3	-3	-3	3	-3	-3	-3	3	-3	-3	3	3	-3	3	-3	3	-3	24	Yes
P130	Mat Eng	1	0	2	3	0	0	3	0	3	0	2	0	0	3	3	1	2	0	-1	2	2	0	0	1	0	2	0	0	2	2	
P130	Mat Eng	2	0	0	0	0	0	0	0	0	0	0	0	0	0	0	0	0	0	0	0	0	0	0	0	0	0	0	0	0	28	Yes
P130	Mat Eng	3	-1	2	3	2	3	3	3	3	3	3	0	0	3	3	0	3	0	2	3	3	0	0	2	0	2	3	3	2	2	
P132	Phys	1	0	3	3	2	2	2	3	3	2	2	3	2	3	2	2	2	2	3	3	2	3	3	3	0	2	2	3	2	3	
P132	Phys	2	3	2	3	-3	3	3	3	3	3	3	3	3	3	3	3	3	3	3	3	3	3	3	-3	3	3	3	-3	3	3	
P132	Phys	3	3	3	3	3	2	3	3	3	3	3	3	3	3	3	3	3	3	3	3	3	3	3	3	2	2	2	2	2	3	
P133	Phys	1	0	1	1	-1	-1	1	-1	0	0	0	1	0	0	0	0	0	0	0	1	-2	-1	1	2	0	-1	2	0	1	7	No
P133	Phys	2	-2	-1	0	-1	-1	1	0	0	1	-1	0	-1	0	-2	0	0	0	2	1	-1	-1	1	1	1	2	1	0	0	5	
P133	Phys	3	0	0	0	0	0	0	0	0	0	0	0	0	0	0	0	0	0	0	0	0	0	0	0	0	0	0	0	0	28	Yes
P134	Phys	1	-1	-1	1	1	0	1	1	1	1	-1	0	0	1	1	-1	1	2	1	1	1	1	-1	1	1	2	1	1	1	4	
P134	Phys	2	-1	1	1	1	-1	1	1	1	1	1	0	-1	1	1	0	1	1	1	1	1	1	1	1	2	1	1	1	1	3	
P134	Phys	3	3	-3	3	3	3	3	-3	3	3	-3	3	3	-2	-1	0	1	2	3	-1	0	-2	3	1	-1	-1	1	1	-1	12	Yes
P135	Phys	1	-1	0	2	3	2	0	1	2	2	-1	3	0	1	3	0	3	1	2	3	1	0	0	1	2	2	1	0	3	2	
P135	Phys	2	0	0	0	0	0	0	0	0	0	0	0	0	0	0	0	0	0	0	0	0	0	0	0	0	0	0	0	0	28	Yes
P135	Phys	3	-2	-2	1	2	-2	-1	1	1	1	-2	0	1	-2	-1	-2	0	-1	-1	0	0	0	-3	-3	-1	-3	1	-2	0	3	
P137	Phys	1	-1	1	2	2	2	2	2	2	2	0	2	-1	2	3	0	2	2	2	2	2	2	2	2	2	3	3	3	2	4	
P137	Phys	2	-2	2	2	2	3	3	3	3	3	3	3	-2	3	3	2	3	3	3	2	3	3	2	2	3	3	3	3	3	3	
P137	Phys	3	-2	2	2	3	-2	2	3	3	3	1	3	-2	3	3	2	3	3	3	3	2	2	2	2	2	2	2	2	2	3	
P138	Phys	1	0	2	1	1	1	1	2	-1	2	1	2	2	2	1	1	1	1	1	1	1	1	2	2	1	-1	1	-2	1	4	
P138	Phys	2	1	1	1	0	1	1	1	1	1	0	1	1	1	1	1	1	1	0	1	0	1	0	1	-1	-1	0	1	1	3	
P138	Phys	3	1	1	1	1	0	1	2	2	1	1	1	1	1	1	2	2	2	2	1	3	1	1	1	2	2	2	2	2	3	
P140	Phys	1	-1	1	3	3	3	3	-2	2	2	-2	2	2	-2	-2	2	-2	-2	-2	2	-2	-2	2	0	0	-1	0	1	0	16	Yes

AttrakDiff Data (Only those who participated in all 3 rounds)																																
ID	CLASS	ROUND	1	2	3	4	5	6	7	8	9	10	11	12	13	14	15	16	17	18	19	20	21	22	23	24	25	26	27	28	Long String	Careless?
P140	Phys	2	-1	1	1	-1	2	2	1	2	2	0	0	0	2	2	0	2	2	0	3	3	1	1	1	2	1	2	1	1	3	
P140	Phys	3	-2	-2	2	2	2	2	1	2	2	2	0	0	2	2	0	1	2	2	2	2	0	0	1	1	1	1	1	1	5	
P143	Phys	1	2	-2	2	1	1	1	-1	1	1	0	0	1	-1	-1	1	-1	-3	0	-3	-3	-3	-3	-3	-3	3	-3	-3	1	6	
P143	Phys	2	3	-2	1	0	-1	-2	3	-2	-1	0	1	2	-3	-2	1	0	1	2	-3	2	1	0	1	-2	-3	2	1	0	1	
P143	Phys	3	3	-3	3	3	3	3	-3	3	3	-3	3	3	-3	-3	3	-3	-3	-3	3	-3	-3	3	3	-3	3	3	3	-3	24	Yes
P145	Math	1	-1	2	-3	1	0	0	1	2	2	0	1	2	0	0	1	1	1	2	2	1	2	-1	2	1	1	1	1	1	2	
P145	Math	2	-2	1	2	0	-1	1	1	3	1	2	0	0	1	0	0	2	1	2	2	3	1	0	1	1	2	1	1	2	2	
P145	Math	3	0	0	0	0	0	0	0	0	0	0	0	0	0	0	0	0	0	0	0	0	0	0	0	0	0	0	0	0	28	Yes
P147	Math	1	0	3	2	2	1	2	2	1	1	-1	1	0	2	2	-2	1	1	3	2	2	2	0	1	-1	2	1	0	2	4	
P147	Math	2	0	2	2	2	1	1	-1	2	1	2	0	-1	1	-1	-2	1	1	2	2	0	1	1	2	2	3	1	0	3	3	
P147	Math	3	0	2	2	2	1	2	1	3	3	0	1	-2	1	2	3	1	2	2	3	2	2	-2	1	2	0	2	1	1	3	
P148	Math	1	0	3	3	3	1	0	3	2	2	1	3	0	3	3	3	3	3	3	3	3	3	1	3	3	3	3	2	2	3	
P148	Math	2	1	2	3	3	1	2	3	3	3	0	1	0	2	0	0	3	3	3	3	3	3	3	3	3	3	3	0	2	3	
P148	Math	3	0	2	2	2	0	3	3	3	2	-1	2	-1	3	3	3	3	3	3	3	2	3	3	-3	3	3	3	2	3	3	
P149	Math	1	1	1	2	2	0	0	0	3	1	0	0	0	0	0	0	0	0	0	1	0	0	0	0	1	1	1	0	0	9	No
P149	Math	2	**2**	**-2**	**2**	**2**	**2**	**2**	1	2	1	0	0	0	0	0	0	0	0	**-3**	**3**	**-3**	**-3**	2	2	0	0	1	0	0	8	Yes
P149	Math	3	1	3	3	3	-3	0	0	3	1	0	0	0	3	2	1	2	0	1	1	0	0	2	2	0	3	1	2	0	3	
P151	Math	1	1	1	1	0	1	0	1	2	2	2	0	0	1	0	0	1	1	1	1	2	0	0	1	1	1	1	1	1	3	
P151	Math	2	1	1	2	1	2	2	1	2	2	1	1	0	1	0	0	2	2	2	2	1	2	1	3	1	2	2	1	1	3	
P151	Math	3	1	0	2	2	3	0	1	2	2	2	0	0	2	0	0	1	2	2	2	2	1	0	1	1	1	1	1	1	2	
P152	Math	1	0	0	2	2	1	0	0	1	1	0	0	0	1	0	2	1	1	1	2	1	0	0	2	2	0	0	1	0	3	
P152	Math	2	0	2	2	0	1	1	2	2	1	0	0	0	0	0	2	0	0	2	2	2	0	0	2	2	2	2	0	0	5	
P152	Math	3	0	2	3	0	3	3	3	3	3	2	2	0	2	2	0	2	2	3	3	3	2	0	3	2	2	2	2	2	2	
P153	Math	1	-2	1	3	0	-1	3	2	0	2	-2	3	3	3	0	0	2	2	3	3	-1	0	1	3	2	2	0	2	0	2	
P153	Math	2	0	0	0	0	0	0	0	0	0	0	0	0	0	0	0	0	0	0	0	0	0	0	0	0	0	0	0	0	28	Yes
P153	Math	3	-3	3	3	-3	3	2	3	3	3	-1	0	0	0	0	0	0	0	0	0	0	0	0	0	0	0	-1	2	3	15	Yes
P154	Math	1	0	2	2	1	0	2	3	3	3	3	0	0	1	0	0	2	1	1	1	2	1	0	0	2	1	2	0	1	2	
P154	Math	2	0	**-1**	**1**	**1**	**1**	**1**	**-1**	**1**	**1**	1	-1	0	0	-1	0	2	1	0	2	0	1	0	-1	0	1	1	0	0	8	Yes

AttrakDiff Data (Only those who participated in all 3 rounds)

ID	CLASS	ROUND	1	2	3	4	5	6	7	8	9	10	11	12	13	14	15	16	17	18	19	20	21	22	23	24	25	26	27	28	Long String	Careless?
P154	Math	3	0	0	0	0	0	0	0	0	0	0	0	0	0	0	0	0	0	0	0	0	0	0	0	0	0	0	0	0	**28**	Yes
P155	Math	1	3	-3	3	3	3	3	3	3	3	-3	3	0	3	-2	2	-2	0	-1	0	-1	0	0	0	-1	0	1	0	0	**6**	
P155	Math	2	-1	3	3	0	0	3	3	3	3	3	3	-2	3	1	1	3	3	3	3	1	3	0	3	1	3	3	3	3	**3**	
P155	Math	3	0	2	3	0	1	3	3	3	3	3	2	-1	3	2	0	1	2	2	3	1	2	0	3	2	3	2	3	2	**2**	
P156	Math	1	-2	2	-2	-2	2	2	2	2	2	2	0	2	2	2	0	2	2	3	3	1	2	0	3	3	3	2	0	2	**4**	
P156	Math	2	-3	3	3	3	0	3	3	3	3	-1	3	-3	3	2	3	3	3	3	3	3	2	2	3	2	3	3	1	3	**3**	
P156	Math	3	0	2	3	3	2	3	3	3	3	-1	2	0	3	3	3	3	3	3	3	2	2	3	3	2	3	1	2	3	**3**	
P157	Math	1	-1	1	-3	-3	-2	1	0	0	1	-2	-1	-1	1	2	1	1	1	1	1	0	0	1	1	-2	2	0	-1	3	**3**	
P157	Math	2	-3	1	-2	0	-3	3	-3	3	-3	-3	1	-3	1	3	1	2	-3	-1	0	-2	-3	1	-2	-3	2	-1	-3	0	**3**	
P157	Math	3	-3	0	-2	0	-3	3	-3	3	-2	0	0	0	0	0	0	-1	-2	-1	0	-3	-1	1	1	-3	3	1	0	0	**6**	
P158	Mec Eng	1	2	1	2	-1	3	-1	0	2	2	1	1	1	2	2	-2	3	2	1	2	2	1	2	2	1	-1	1	1	1	**3**	
P158	Mec Eng	2	0	0	0	0	0	0	0	0	0	0	0	0	0	0	0	0	0	0	0	0	0	0	0	0	0	0	0	0	**28**	Yes
P158	Mec Eng	3	0	0	0	0	0	0	0	0	0	0	0	0	0	0	0	0	0	0	0	0	0	0	0	0	0	0	0	0	**28**	Yes
P159	Mec Eng	1	0	1	-3	-2	0	-2	3	3	2	0	2	2	2	1	1	3	2	2	3	2	1	-1	2	1	2	-1	2	2	**2**	
P159	Mec Eng	2	-1	0	1	2	1	2	2	-2	1	-1	2	0	2	1	1	3	2	1	2	1	2	0	1	1	3	0	1	1	**2**	
P159	Mec Eng	3	-1	0	1	2	0	1	2	1	0	0	2	0	2	0	1	3	0	1	1	0	0	0	1	0	1	-1	2	0	**3**	
P160	Mec Eng	1	-3	2	3	0	2	2	2	2	3	3	1	-1	2	2	1	3	1	2	2	3	1	0	2	2	3	2	1	2	**2**	
P160	Mec Eng	2	-2	2	1	-1	0	1	1	2	2	2	1	0	1	3	2	2	3	1	2	1	0	0	0	0	1	1	0	1	**4**	
P160	Mec Eng	3	-2	1	2	-2	-1	1	2	1	2	0	0	0	1	1	2	3	2	1	2	0	0	1	2	0	2	2	1	2	**3**	
P161	Mec Eng	1	0	2	3	3	1	2	1	3	2	2	2	-2	2	3	2	3	2	3	3	3	2	2	2	3	3	3	3	2	**2**	
P161	Mec Eng	2	2	2	3	3	3	2	2	3	3	1	3	1	2	3	2	2	3	3	3	2	2	2	2	2	2	3	3	2	**3**	
P161	Mec Eng	3	2	2	3	3	2	2	2	3	3	2	2	3	2	2	3	2	3	3	3	3	3	2	3	3	2	2	2	2	**2**	
P162	Mec Eng	1	-1	1	2	-2	0	3	3	2	1	2	3	-1	3	2	-2	2	1	0	2	2	1	-2	2	2	3	3	3	2	**3**	
P162	Mec Eng	2	-1	3	3	2	1	2	2	2	2	2	2	-3	3	3	2	3	2	2	3	1	1	1	2	3	3	3	3	3	**3**	
P162	Mec Eng	3	-2	2	2	3	2	3	3	2	2	2	2	-3	3	3	2	3	3	3	2	2	0	1	2	2	3	2	3	2	**3**	
P163	Mec Eng	1	-1	1	2	3	1	2	2	2	3	2	2	2	2	3	2	2	2	3	3	2	3	2	3	1	1	2	2	2	**2**	
P163	Mec Eng	2	-1	2	2	2	2	1	2	2	2	0	2	0	3	2	3	2	2	2	2	3	2	2	3	2	2	2	3	2	**3**	
P163	Mec Eng	3	-1	1	3	3	0	1	2	2	2	2	2	0	3	2	1	2	2	3	2	2	1	2	3	2	2	2	2	2	**2**	

AttrakDiff Data (Only those who participated in all 3 rounds)																																
ID	CLASS	ROUND	1	2	3	4	5	6	7	8	9	10	11	12	13	14	15	16	17	18	19	20	21	22	23	24	25	26	27	28	Long String	Careless?
P164	Mec Eng	1	2	2	2	2	-1	1	2	2	3	0	2	-1	2	1	-1	3	2	3	3	0	2	0	-3	3	1	3	2	2	**2**	
P164	Mec Eng	2	-1	1	2	-2	-2	1	3	2	2	0	2	-1	1	0	-2	0	2	1	2	-2	1	2	-1	1	2	-1	2	2	**2**	
P164	Mec Eng	3	-1	-1	2	1	1	1	2	2	2	1	0	1	1	1	0	2	2	2	2	0	1	1	2	1	1	2	2	1	**3**	
P165	Mec Eng	1	-1	-1	1	0	0	3	0	1	1	0	1	0	2	0	-1	0	0	1	2	0	0	0	3	0	2	0	-1	0	**3**	
P165	Mec Eng	2	0	0	0	0	0	3	-3	1	0	-1	0	1	0	1	1	0	0	0	1	1	1	-1	-1	1	1	0	-1	2	**6**	
P165	Mec Eng	3	**-3**	**3**	**-3**	**-3**	**-3**	**-3**	**3**	**-3**	**-3**	**3**	**-3**	**-3**	-3	-3	-3	-3	-3	-3	-3	**-3**	**-3**	**3**	-3	-3	-3	-3	-3	-3	**12**	Yes
P166	Mec Eng	1	0	1	-1	1	1	0	1	1	1	0	0	-1	1	0	0	0	1	2	1	1	1	0	1	2	1	1	0	0	**3**	
P166	Mec Eng	2	0	1	2	2	1	1	1	2	2	1	1	-3	2	1	0	1	1	3	2	2	2	0	1	-2	3	1	2	1	**2**	
P166	Mec Eng	3	0	0	1	1	1	1	-1	3	2	-1	0	0	1	1	0	1	0	1	0	1	1	0	1	1	3	1	0	0	**5**	
P167	Mec Eng	1	-2	3	3	2	2	2	3	3	3	2	2	1	2	2	2	3	3	3	3	3	1	-1	2	2	2	2	3	2	**3**	
P167	Mec Eng	2	-2	3	3	3	1	2	2	3	3	2	2	0	2	2	1	3	3	3	3	3	2	-2	2	2	2	2	2	2	**3**	
P167	Mec Eng	3	-2	2	3	2	1	2	2	2	2	2	3	1	2	3	2	3	2	3	3	2	2	1	2	2	2	2	2	2	**2**	
P168	Mec Eng	1	0	2	0	0	1	0	-1	-1	2	0	-1	0	0	0	2	1	1	2	1	-1	0	0	1	-1	2	2	-1	1	**3**	
P168	Mec Eng	2	0	0	1	2	0	2	1	0	1	-1	0	-1	1	0	0	2	2	3	1	0	1	1	1	1	2	1	2	2	**2**	
P168	Mec Eng	3	0	1	1	0	0	2	2	0	2	1	3	0	2	2	-2	2	2	2	2	0	0	1	0	2	1	2	2	1	**6**	
P169	Mec Eng	1	0	0	3	0	-2	-1	0	1	1	-2	1	0	0	0	-3	0	0	0	0	0	0	0	0	0	0	0	0	0	**13**	No
P169	Mec Eng	2	2	2	2	0	2	-3	3	3	3	-3	3	-2	2	-2	0	2	2	2	2	1	2	-2	3	2	3	3	3	2	**4**	
P169	Mec Eng	3	3	3	3	2	3	0	3	3	3	-1	3	0	3	0	3	3	1	3	3	3	0	-2	-2	2	2	3	2	2	**4**	
P170	Mec Eng	1	0	3	3	3	1	3	3	3	3	1	3	-1	3	3	3	3	3	3	3	3	3	-3	3	3	3	3	3	3	**3**	
P170	Mec Eng	2	2	2	2	2	0	2	2	3	2	0	2	0	2	2	0	3	2	2	2	2	0	-1	2	2	2	2	2	2	**2**	
P170	Mec Eng	3	-3	3	3	3	-1	3	3	3	3	2	3	-2	3	3	3	3	3	3	3	3	3	0	3	3	3	3	3	3	**3**	
P171	Mec Eng	1	0	2	1	1	0	2	3	-3	2	1	3	-3	0	0	1	2	3	3	2	3	0	3	2	3	3	0	3	3	**2**	
P171	Mec Eng	2	0	0	1	1	0	1	1	1	0	0	0	0	1	0	0	2	2	3	1	1	0	0	1	1	1	0	1	1	**4**	
P171	Mec Eng	3	0	0	0	0	0	0	0	0	0	0	0	0	0	0	0	0	0	0	0	0	0	0	0	0	0	0	0	0	**28**	Yes
P172	Mec Eng	1	-1	2	2	2	0	-2	1	2	2	0	1	-2	3	0	0	1	2	3	2	0	2	0	2	0	1	0	2	1	**2**	
P172	Mec Eng	2	0	1	2	2	0	0	0	1	-2	0	0	-2	0	2	0	1	1	3	3	0	0	0	0	1	1	1	3	0	**4**	
P172	Mec Eng	3	-1	0	0	0	0	0	0	0	0	0	0	0	0	0	0	0	0	0	0	0	0	0	0	0	0	0	0	0	**27**	Yes
P173	Mec Eng	1	-1	2	2	0	2	1	2	2	3	2	1	2	1	2	0	1	1	2	2	2	1	0	1	1	-1	2	2	1	**2**	

AttrakDiff Data (Only those who participated in all 3 rounds)																																	
ID	CLASS	ROUND	1	2	3	4	5	6	7	8	9	10	11	12	13	14	15	16	17	18	19	20	21	22	23	24	25	26	27	28	Long String	Careless?
P173	Mec Eng	2	-2	2	2	1	0	1	2	2	2	2	1	0	1	1	1	1	1	1	2	2	1	0	1	1	-1	1	1	-1	4	
P173	Mec Eng	3	0	0	-1	0	0	-1	0	0	-1	-1	0	0	-2	0	0	-1	-1	1	-1	0	-1	1	1	1	-1	-1	0	-1	3	
P174	Mec Eng	1	0	1	2	1	-2	2	0	2	2	0	2	1	2	0	1	3	2	2	3	2	0	2	2	0	2	2	2	1	2	
P174	Mec Eng	2	-2	2	0	0	1	2	2	2	2	2	3	1	2	-2	2	2	2	1	2	1	0	0	1	2	0	2	0	2	2	
P174	Mec Eng	3	0	0	0	0	0	1	2	2	1	1	2	0	2	0	1	2	2	1	2	2	1	0	0	1	0	0	-2	2	5	
P175	Mec Eng	1	-3	0	0	1	-1	3	3	1	-3	0	1	-1	1	1	0	2	-2	3	0	-1	0	-1	3	1	3	2	1	2	3	
P175	Mec Eng	2	-3	2	0	0	0	3	3	3	0	3	1	-3	3	-1	3	3	0	3	3	3	0	-3	2	3	3	1	2	1	3	
P175	Mec Eng	3	-2	3	2	3	0	2	3	3	0	3	2	0	3	0	0	3	3	3	3	3	3	0	3	1	3	2	0	1	3	
P177	Math	1	1	2	1	1	1	2	2	2	2	-1	3	0	2	2	2	2	2	2	1	1	1	2	2	2	2	1	2	0	3	
P177	Math	2	-3	2	-3	-3	1	-3	3	-3	-3	3	-1	1	-2	-2	2	-2	-2	-2	2	-2	-1	2	2	-2	-1	2	2	-2	8	Yes
P177	Math	3	3	-3	3	3	3	3	-3	3	3	-1	1	1	0	3	2	0	0	-2	1	-2	-2	1	2	-2	-2	2	2	-2	9	Yes
P178	Math	1	0	1	0	3	0	3	1	0	0	0	2	1	3	0	0	0	0	2	0	1	0	0	0	-1	3	1	-1	1	4	
P178	Math	2	-2	0	0	3	-2	3	3	2	1	0	3	2	3	2	0	1	2	3	2	3	0	3	3	2	3	3	3	3	2	
P178	Math	3	0	0	0	0	0	0	0	0	0	0	0	0	0	0	0	0	0	0	0	0	0	0	0	0	0	0	0	0	28	Yes
P183	Mat Eng	1	-1	1	2	-2	2	2	2	2	2	2	2	2	2	2	2	2	1	2	2	2	2	2	2	1	2	2	2	2	2	
P183	Mat Eng	2	-2	-1	1	1	2	2	1	1	1	1	1	1	1	1	1	1	1	1	2	-1	1	1	1	1	1	2	1	1	3	
P183	Mat Eng	3	-1	-1	-1	1	-2	1	-2	-1	-2	-2	-1	-1	-1	0	-1	-1	-1	1	-1	-2	0	0	0	-2	0	-1	1	0	3	
P189	Mat Eng	1	-1	1	1	0	1	1	0	1	1	0	1	1	0	1	1	1	0	1	1	1	0	0	1	0	1	0	-1	1	2	
P189	Mat Eng	2	-1	2	0	1	0	1	1	1	1	0	1	1	0	1	1	0	0	0	1	0	0	-1	-1	1	1	0	1	0	4	
P189	Mat Eng	3	-1	1	0	1	0	1	1	1	1	1	1	1	0	-1	1	1	0	1	1	-1	0	-1	1	-1	1	1	0	2	2	
P191	Phys	1	-1	1	2	-2	-1	2	2	2	0	2	2	-2	2	2	2	2	3	3	2	1	2	2	1	1	2	3	1	1	3	
P191	Phys	2	0	0	0	0	0	0	0	0	0	0	0	0	0	0	0	0	0	0	0	0	0	0	0	0	0	0	0	0	28	Yes
P191	Phys	3	0	0	0	0	0	0	0	0	0	0	0	0	0	0	0	0	0	0	0	0	0	0	0	0	0	0	0	0	28	Yes
P193	Mec Eng	1	-2	2	2	2	-1	3	2	3	3	1	2	-3	2	-1	2	2	1	2	1	1	0	2	2	2	3	3	3	-2	2	
P193	Mec Eng	2	-3	1	3	3	-2	1	0	2	3	3	2	-2	3	3	3	3	3	3	3	3	3	-3	3	3	2	3	3	3	3	
P193	Mec Eng	3	0	0	0	0	0	0	0	0	0	0	0	-2	2	2	-2	2	3	3	-3	3	3	-3	-3	2	1	-1	-1	1	11	Yes
P195	Mat Eng	1	2	2	2	2	-1	2	2	3	3	0	3	0	3	3	-1	3	3	3	3	3	3	0	3	3	3	3	3	3	3	
P195	Mat Eng	2	3	3	3	3	1	0	3	3	3	-1	1	0	3	3	-1	3	3	3	3	3	0	3	3	3	3	0	3	3	3	

AttrakDiff Data (Only those who participated in all 3 rounds)

ID	CLASS	ROUND	1	2	3	4	5	6	7	8	9	10	11	12	13	14	15	16	17	18	19	20	21	22	23	24	25	26	27	28	Long String	Careless?
P195	Mat Eng	3	-3	3	3	3	-2	3	3	0	3	-2	3	-1	3	2	-3	3	3	3	3	0	0	2	1	-1	-1	2	3	-2	4	

4. Final Dataset

In this section we present the final dataset per method, after removing careless respondents.

Final UEQ Data (After Removing Careless Respondents)																												
ID	CLASS	ROUND	1	2	3	4	5	6	7	8	9	10	11	12	13	14	15	16	17	18	19	20	21	22	23	24	25	26
P3	Phys	1	3	3	3	3	3	3	3	-1	2	3	3	3	3	3	3	3	3	3	3	3	3	3	3	3	3	3
P3	Phys	2	3	3	3	2	3	2	3	0	3	3	3	3	3	3	3	3	3	3	3	3	2	3	3	3	3	3
P3	Phys	3	2	2	2	2	2	2	2	-1	1	2	2	2	0	1	2	2	2	1	2	2	2	2	2	2	2	2
P5	Phys	1	2	-2	0	-3	0	-1	-1	0	0	-1	-3	-2	-3	0	2	0	0	-2	0	0	-2	0	0	-1	-1	2
P5	Phys	2	1	0	-2	-3	1	1	0	-2	0	0	-1	0	-3	3	1	0	0	-3	0	1	-2	0	1	0	1	3
P5	Phys	3	0	-2	0	-3	-1	-2	0	-3	0	1	-3	-3	-3	1	2	0	0	-3	0	0	-2	0	0	-1	0	0
P10	Math	1	2	2	2	0	3	2	2	1	1	0	-2	3	-2	1	1	2	1	3	2	3	1	2	2	2	2	0
P10	Math	2	2	1	2	1	3	1	3	2	2	2	-2	2	1	2	1	1	2	3	2	3	2	2	2	2	2	0
P10	Math	3	1	1	0	0	2	0	1	0	1	0	-2	2	-2	1	1	1	-1	1	0	1	0	0	2	0	0	0
P12	Mec Eng	1	0	-1	1	-2	1	2	0	-2	0	0	2	1	-1	1	2	2	-1	0	-1	2	-2	-1	-1	-1	-1	1
P12	Mec Eng	2	2	1	1	2	2	0	2	0	2	2	2	2	0	0	2	1	2	1	0	1	0	1	0	1	2	-1
P12	Mec Eng	3	0	0	1	1	1	0	1	-1	1	2	1	0	0	0	1	1	0	0	1	1	0	1	1	1	1	1
P15	Math	1	3	2	-2	0	0	1	2	2	-1	-1	1	2	-1	1	2	2	1	0	-1	1	1	1	1	2	2	-2
P15	Math	2	1	1	1	0	1	0	1	0	1	-1	1	1	1	0	1	1	1	0	1	1	0	1	1	1	1	1
P15	Math	3	3	3	1	0	-1	-1	1	-1	-1	-1	1	1	1	1	1	1	-1	0	0	1	-1	1	0	0	-1	1
P17	Mat Eng	1	-1	-1	2	0	0	-1	0	0	2	2	-2	1	0	1	2	0	2	1	0	1	-1	2	2	2	1	2
P17	Mat Eng	2	-1	-1	2	0	1	-1	0	0	2	2	0	0	1	0	2	0	1	0	1	1	-2	2	2	2	0	2
P17	Mat Eng	3	-1	0	-1	0	1	-1	1	1	2	1	1	1	-1	0	1	0	1	-1	0	1	-1	1	1	1	0	1
P19	Mat Eng	1	3	1	-3	0	3	3	3	0	3	3	3	3	-1	3	3	3	3	3	3	3	2	3	3	3	3	3
P19	Mat Eng	2	3	2	3	0	3	2	3	0	2	3	3	3	-1	3	3	1	3	3	2	2	0	3	3	3	3	3
P19	Mat Eng	3	-2	-2	3	-3	3	-2	2	-3	0	3	3	0	-3	0	3	-3	3	0	0	3	-3	3	3	3	0	3
P23	Mat Eng	1	1	1	1	2	2	1	1	-2	2	1	2	1	2	1	1	1	2	1	2	2	2	1	2	1	2	1
P23	Mat Eng	2	1	2	-2	-1	0	2	1	0	-1	-1	2	0	1	2	0	1	-1	-1	0	0	-1	1	0	-1	0	2
P23	Mat Eng	3	2	2	-2	-2	-2	1	0	0	-1	0	0	0	1	0	1	1	0	0	-1	0	0	1	-1	0	-1	1
P24	Mat Eng	1	2	2	3	1	3	2	2	2	2	3	2	3	-1	2	2	2	2	2	1	3	0	3	2	2	2	2
P24	Mat Eng	2	0	-1	1	-2	1	-1	0	-2	2	1	-2	0	-3	0	1	-1	0	-1	-1	0	-2	-1	0	-2	-3	2

Final UEQ Data (After Removing Careless Respondents)																												
**ID**	**CLASS**	**ROUND**	**1**	**2**	**3**	**4**	**5**	**6**	**7**	**8**	**9**	**10**	**11**	**12**	**13**	**14**	**15**	**16**	**17**	**18**	**19**	**20**	**21**	**22**	**23**	**24**	**25**	**26**
P24	Mat Eng	3	0	1	3	-2	3	-1	2	-2	3	2	1	3	-1	2	3	2	3	2	-1	2	-2	3	1	2	2	-3
P25	Mat Eng	1	1	1	1	2	2	0	1	2	1	1	1	1	2	1	1	1	1	2	2	2	1	1	1	2	1	0
P25	Mat Eng	2	-1	0	-1	1	0	0	1	0	-1	0	-1	1	1	1	1	0	0	2	1	2	1	1	1	1	1	1
P25	Mat Eng	3	-1	0	1	1	1	1	1	1	0	1	1	1	0	1	0	0	0	1	1	1	1	1	1	1	1	1
P28	Mat Eng	1	2	2	1	1	2	1	2	-2	-1	3	3	3	0	2	3	2	2	2	2	3	-1	2	1	2	2	3
P28	Mat Eng	2	-1	-1	-1	1	3	-1	1	0	1	1	0	1	0	1	1	2	1	-1	1	1	-1	-1	1	0	1	2
P28	Mat Eng	3	1	1	-1	1	1	-1	1	1	1	3	2	3	1	1	3	2	1	1	2	1	1	1	2	1	1	3
P30	Mat Eng	1	3	3	0	-3	0	1	1	0	-1	-1	1	3	-1	3	3	3	3	3	3	3	0	3	3	3	0	-3
P30	Mat Eng	2	0	-2	-3	-2	-2	1	3	3	3	3	3	3	3	3	1	3	3	3	0	3	-2	3	3	-1	3	3
P30	Mat Eng	3	1	3	-1	-3	-1	1	2	1	2	0	0	-1	2	0	3	0	3	-1	2	1	-3	-2	1	0	-1	3
P40	Phys	1	2	2	-2	-2	1	0	2	0	2	2	2	2	1	2	1	2	1	2	2	2	2	2	2	2	2	2
P40	Phys	2	3	3	3	3	3	0	3	1	3	3	3	3	3	3	3	3	3	3	3	3	3	3	3	3	3	3
P40	Phys	3	2	2	2	2	2	0	2	1	2	2	3	3	2	2	2	2	2	2	2	2	2	2	2	2	2	2
P44	Phys	1	1	1	0	0	0	-1	0	2	-2	0	0	0	1	1	0	0	1	-1	1	0	-1	2	0	0	1	0
P44	Phys	2	-3	-3	0	0	0	0	-2	-2	-2	0	-2	-2	-2	-2	-1	-2	-1	-3	0	-1	-1	-1	-1	0	-3	0
P44	Phys	3	-1	0	1	1	0	0	0	-2	0	0	-1	-1	0	-1	0	-1	-2	-3	0	0	-2	0	-1	1	1	0
P47	Phys	1	2	2	2	1	2	2	2	1	1	2	2	2	1	2	2	2	2	2	2	2	1	2	2	2	2	2
P47	Phys	2	2	2	2	2	1	1	2	0	-1	2	2	2	1	2	2	1	2	3	2	2	2	2	2	2	2	1
P47	Phys	3	2	1	1	1	2	1	1	2	2	2	1	2	0	2	2	2	2	0	1	1	1	2	2	2	1	1
P54	Math	1	3	2	3	2	1	1	2	0	1	2	2	3	1	2	3	2	2	3	2	2	2	3	2	2	1	1
P54	Math	2	2	2	2	2	1	1	2	1	1	2	2	3	1	2	2	2	1	2	2	3	1	2	2	1	2	3
P54	Math	3	2	3	2	1	2	0	2	0	2	3	1	2	0	2	2	2	3	2	1	2	1	2	3	2	3	2
P55	Math	1	1	0	0	1	1	0	0	0	0	0	0	1	0	1	1	1	1	1	1	1	0	0	1	0	0	0
P55	Math	2	0	0	0	0	1	0	0	-1	0	0	0	0	0	0	0	0	0	1	0	0	0	0	1	0	0	0
P55	Math	3	1	1	2	0	1	2	1	-1	-1	2	0	2	-1	1	1	1	1	1	0	1	1	0	1	1	1	1
P56	Math	1	2	1	3	1	2	2	1	-1	2	3	3	3	0	2	3	3	3	3	2	3	1	2	3	2	2	3
P56	Math	2	1	0	3	1	2	2	2	1	2	3	1	2	-3	3	3	3	3	2	3	3	1	3	2	1	2	3
P56	Math	3	2	2	3	2	3	3	3	0	2	3	3	3	2	2	3	3	3	3	1	3	3	3	3	2	3	3

Final UEQ Data (After Removing Careless Respondents)																												
**ID**	**CLASS**	**ROUND**	**1**	**2**	**3**	**4**	**5**	**6**	**7**	**8**	**9**	**10**	**11**	**12**	**13**	**14**	**15**	**16**	**17**	**18**	**19**	**20**	**21**	**22**	**23**	**24**	**25**	**26**
P57	Math	1	1	1	2	0	0	1	2	0	0	0	0	0	0	2	2	2	1	0	0	0	0	0	2	1	0	0
P57	Math	2	3	2	-2	1	2	2	3	2	-1	1	2	2	1	2	2	2	2	3	2	2	2	2	2	2	2	2
P57	Math	3	3	2	2	2	2	1	3	1	2	0	1	2	1	2	2	2	1	2	2	2	1	1	2	1	2	-1
P60	Math	1	3	3	1	-3	0	1	1	-1	0	1	0	1	-1	1	2	1	1	1	1	1	1	1	2	2	2	-2
P60	Math	2	3	-2	2	-3	2	2	2	-3	2	2	-2	2	-3	3	3	2	2	3	2	2	0	3	3	3	3	3
P60	Math	3	1	-1	1	-3	0	1	1	-1	0	1	-3	2	-3	1	2	1	3	0	-2	-1	-3	-3	0	1	2	0
P61	Math	1	0	2	2	1	2	2	1	1	1	2	2	2	2	2	1	1	2	2	1	2	2	2	3	2	2	-2
P61	Math	2	2	2	2	1	2	1	1	2	2	2	1	2	-1	1	0	1	0	-1	-3	1	1	1	1	0	1	-1
P61	Math	3	0	2	2	1	1	0	1	1	1	1	1	1	2	2	2	2	1	1	1	1	1	1	1	1	1	1
P64	Mec Eng	1	3	2	2	2	3	2	3	1	0	2	2	3	2	2	2	3	1	3	2	1	2	2	2	3	3	2
P64	Mec Eng	2	1	2	1	1	2	0	1	0	2	2	3	2	2	1	2	3	3	2	2	2	2	3	3	2	2	2
P64	Mec Eng	3	0	1	1	0	2	0	1	-1	1	2	3	2	1	1	2	2	2	1	1	1	1	1	2	2	1	1
P68	Mec Eng	1	1	1	0	1	0	0	1	0	0	1	1	2	1	1	-1	1	-1	0	1	1	1	1	2	2	2	1
P68	Mec Eng	2	-2	-2	2	1	2	-1	-1	0	1	0	-1	2	-3	-1	-2	-1	0	-1	-1	0	0	1	2	1	1	0
P68	Mec Eng	3	0	0	1	2	1	0	1	0	0	1	-1	0	1	1	0	1	-1	-1	0	1	0	1	1	0	0	-1
P72	Mec Eng	1	1	1	2	0	0	-1	-2	-1	1	1	1	2	1	1	1	1	-1	1	1	1	1	1	-1	-1	1	1
P72	Mec Eng	2	1	-1	1	1	1	1	1	1	1	1	1	2	2	1	2	2	1	1	2	2	-1	2	1	1	1	2
P72	Mec Eng	3	-1	0	-1	-1	3	1	0	0	0	1	-2	1	-1	0	1	-1	0	0	0	1	-2	-1	1	1	-1	1
P74	Mec Eng	1	3	3	2	2	3	2	3	0	0	3	2	3	1	2	2	2	3	3	3	3	2	2	3	3	2	2
P74	Mec Eng	2	2	2	1	2	2	1	2	-1	1	2	1	2	1	2	2	2	2	1	2	2	2	2	2	2	2	2
P74	Mec Eng	3	1	1	1	1	2	2	2	0	0	3	2	3	1	1	3	2	2	2	2	2	2	2	2	2	2	3
P75	Mec Eng	1	2	2	1	1	1	1	1	-1	1	1	1	2	1	1	1	1	1	2	1	1	1	1	1	1	1	-1
P75	Mec Eng	2	1	2	2	2	2	1	2	1	2	2	1	2	1	1	0	2	1	1	2	2	1	2	2	2	2	0
P75	Mec Eng	3	2	2	1	1	1	1	2	0	1	1	1	1	1	1	1	1	1	1	2	2	1	2	2	1	1	1
P77	Mec Eng	1	2	2	2	3	3	0	2	-3	2	3	3	3	2	3	3	3	3	3	3	2	3	3	3	3	3	3
P77	Mec Eng	2	2	3	2	2	3	2	3	2	2	-1	3	3	2	3	3	3	2	3	3	2	3	3	2	3	3	2
P77	Mec Eng	3	0	3	2	2	3	1	2	-2	2	2	3	2	0	3	3	3	3	1	2	3	3	3	2	2	3	3
P78	Mec Eng	1	2	2	2	1	3	2	3	1	1	3	1	3	0	2	3	2	2	2	2	2	2	2	3	3	3	3

Final UEQ Data (After Removing Careless Respondents)																												
**ID**	**CLASS**	**ROUND**	**1**	**2**	**3**	**4**	**5**	**6**	**7**	**8**	**9**	**10**	**11**	**12**	**13**	**14**	**15**	**16**	**17**	**18**	**19**	**20**	**21**	**22**	**23**	**24**	**25**	**26**
P78	Mec Eng	2	3	3	3	2	3	2	3	0	2	3	3	3	0	3	3	3	3	2	3	3	2	3	2	2	2	3
P78	Mec Eng	3	3	3	3	3	3	3	3	0	3	3	3	2	0	0	3	3	2	3	3	2	3	3	2	2	2	-2
P80	Mec Eng	1	1	1	1	1	2	0	2	0	1	2	-2	2	-1	-1	1	1	0	-1	0	1	1	1	-1	-2	0	0
P80	Mec Eng	2	0	-1	2	0	0	0	0	0	1	1	0	1	1	0	0	0	0	0	0	1	-1	1	1	0	1	-1
P80	Mec Eng	3	1	2	1	0	1	1	1	0	3	2	2	3	1	2	3	2	2	2	3	2	1	3	3	2	3	-2
P85	Mat Eng	1	3	0	3	0	2	2	3	0	2	2	2	3	0	3	3	2	2	3	2	2	0	2	2	2	1	3
P85	Mat Eng	2	1	0	3	-1	2	1	3	0	1	3	0	3	-1	3	3	1	3	3	2	2	-1	3	2	3	1	3
P85	Mat Eng	3	1	-1	2	-1	1	0	1	-1	1	3	1	2	-2	0	3	0	2	2	1	2	-1	0	2	1	0	2
P94	Mec Eng	1	0	-1	-2	-3	1	-2	0	2	0	0	0	0	-1	0	0	0	0	0	0	0	-1	0	0	0	0	0
P94	Mec Eng	2	0	1	0	1	0	0	0	3	3	0	0	2	-1	0	0	0	2	-1	2	1	1	1	0	0	0	0
P94	Mec Eng	3	-1	-1	0	-1	-2	-2	-1	1	-2	-1	0	1	1	-2	-1	0	0	-2	0	0	0	0	0	0	0	0
P99	Mat Eng	1	2	2	3	2	2	2	3	0	2	3	3	3	2	3	3	3	3	3	2	3	3	3	3	2	2	3
P99	Mat Eng	2	2	2	3	2	0	1	2	0	2	2	3	3	3	2	2	2	2	2	3	3	3	3	3	3	3	2
P99	Mat Eng	3	3	3	3	3	0	3	3	0	-1	3	3	3	3	3	3	0	3	3	3	3	3	3	3	3	3	3
P101	Mec Eng	1	0	1	1	0	0	0	1	-1	1	-1	1	1	1	1	-1	1	1	1	1	1	1	1	1	1	1	1
P101	Mec Eng	2	1	1	1	0	1	1	1	2	1	0	1	1	0	1	1	1	1	1	1	1	1	1	-1	1	1	1
P101	Mec Eng	3	1	1	1	0	0	1	1	0	1	-1	1	1	0	1	-1	1	0	1	1	1	1	1	-1	-1	1	1
P104	Mat Eng	1	0	0	2	2	2	0	2	0	2	0	2	2	0	2	0	2	0	2	0	0	0	3	3	3	2	2
P104	Mat Eng	2	1	1	0	1	1	0	1	1	2	0	1	1	0	1	1	1	0	1	1	1	0	2	2	2	1	1
P104	Mat Eng	3	1	1	1	1	1	0	1	1	1	0	1	1	0	1	1	1	1	0	0	0	0	0	0	0	0	0

Final AttrakDiff Data (After Removing Careless Respondents)

ID	CLASS	ROUND	1	2	3	4	5	6	7	8	9	10	11	12	13	14	15	16	17	18	19	20	21	22	23	24	25	26	27	28
P105	Phys	1	0	1	1	1	0	1	1	1	1	1	1	-2	0	0	0	1	2	2	2	1	0	0	1	2	1	1	0	0
P105	Phys	2	0	0	0	0	1	2	0	2	2	-2	1	0	-2	-2	2	-1	-2	-2	1	-2	-2	1	1	-1	-2	1	2	-1
P105	Phys	3	-1	1	2	2	2	1	1	1	1	-2	1	1	0	0	0	1	1	1	2	1	0	0	0	1	0	1	0	0
P111	Math	1	-1	1	3	2	2	1	2	-2	-1	0	0	0	2	2	0	2	2	3	3	1	1	0	2	2	2	2	0	2
P111	Math	2	-1	0	2	1	0	1	3	2	2	0	2	-1	2	2	-1	2	2	2	2	1	2	0	2	2	2	2	1	2
P111	Math	3	-1	0	2	2	0	1	2	2	2	1	1	-1	2	2	0	2	2	1	2	2	2	0	2	2	2	2	0	2
P121	Mat Eng	1	-1	2	2	2	1	1	2	2	3	2	1	1	2	2	3	2	2	2	3	2	1	1	2	1	2	2	1	2
P121	Mat Eng	2	-1	0	1	1	1	0	1	1	1	1	1	1	1	1	1	2	1	1	2	0	0	1	1	1	2	0	0	1
P121	Mat Eng	3	-1	1	2	2	1	1	2	1	1	1	1	1	2	2	2	2	2	2	2	2	1	1	1	2	2	2	1	1
P122	Mat Eng	1	-2	3	3	-3	0	0	3	3	3	2	2	0	2	0	0	2	0	2	3	0	0	0	0	3	1	0	0	0
P122	Mat Eng	2	0	1	0	1	0	0	0	1	1	0	1	0	1	0	0	1	1	1	1	1	-1	0	0	1	1	0	0	1
P122	Mat Eng	3	0	1	1	-1	0	0	0	1	1	0	0	1	0	0	0	1	0	0	1	0	0	0	0	0	0	0	0	0
P124	Mat Eng	1	-1	1	2	2	2	2	0	2	2	-2	0	1	1	3	1	0	2	2	2	2	2	-1	2	2	2	2	2	2
P124	Mat Eng	2	-1	1	0	1	-1	1	1	0	-1	-1	1	-1	1	-1	-3	1	1	1	1	-2	1	-1	-1	1	1	0	1	0
P124	Mat Eng	3	3	1	0	-1	-1	0	1	0	0	0	3	-1	0	1	0	2	-1	-1	-1	0	0	-3	-1	1	1	1	1	1
P126	Mat Eng	1	1	1	0	-1	0	1	1	2	2	0	0	2	1	2	1	2	0	2	2	1	2	1	0	1	3	2	1	2
P126	Mat Eng	2	-2	-1	0	1	-1	2	-1	-1	-1	2	1	2	0	1	-1	1	-1	1	-1	1	-2	1	1	0	2	1	1	1
P126	Mat Eng	3	-1	0	1	0	-1	1	0	0	0	2	1	-1	2	1	-2	2	-1	2	1	0	-1	-1	-1	-1	3	-1	0	0
P127	Mat Eng	1	-1	0	1	0	0	1	1	1	2	0	0	0	1	3	1	3	0	2	3	0	0	3	1	0	1	1	2	0
P127	Mat Eng	2	-2	2	1	2	0	1	3	3	3	1	2	0	3	2	1	2	0	3	3	1	0	0	3	1	1	0	1	2
P127	Mat Eng	3	0	1	1	0	0	0	2	2	1	0	1	0	1	1	1	1	0	1	2	0	0	0	1	1	1	1	1	1
P132	Phys	1	0	3	3	2	2	2	3	3	2	2	3	2	3	2	2	2	2	3	3	2	3	3	3	0	2	2	3	2
P132	Phys	2	3	2	3	-3	3	3	3	3	3	3	3	3	3	3	3	3	3	3	3	3	3	3	-3	3	3	3	-3	3
P132	Phys	3	3	3	3	3	2	3	3	3	3	3	3	3	3	3	3	3	3	3	3	3	3	3	3	2	2	2	2	2
P137	Phys	1	-1	1	2	2	2	2	2	2	2	0	2	-1	2	3	0	2	2	2	2	2	2	2	2	2	3	3	3	2
P137	Phys	2	-2	2	2	2	3	3	3	3	3	3	3	-2	3	3	2	3	3	3	2	3	3	2	2	3	3	3	3	3
P137	Phys	3	-2	2	2	3	-2	2	3	3	3	1	3	-2	3	3	2	3	3	3	3	2	2	2	2	2	2	2	2	2

Final AttrakDiff Data (After Removing Careless Respondents)																														
ID	CLASS	ROUND	1	2	3	4	5	6	7	8	9	10	11	12	13	14	15	16	17	18	19	20	21	22	23	24	25	26	27	28
P138	Phys	1	0	2	1	1	1	1	2	-1	2	1	2	2	2	1	1	1	1	1	1	1	1	2	2	1	-1	1	-2	1
P138	Phys	2	1	1	1	0	1	1	1	1	1	0	1	1	1	1	1	1	1	0	1	0	1	0	1	-1	-1	0	1	1
P138	Phys	3	1	1	1	1	0	1	2	2	1	1	1	1	1	1	2	2	2	2	1	3	1	1	1	2	2	2	2	2
P147	Math	1	0	3	2	2	1	2	2	1	1	-1	1	0	2	2	-2	1	1	3	2	2	2	0	1	-1	2	1	0	2
P147	Math	2	0	2	2	2	1	1	-1	2	1	2	0	-1	1	-1	-2	1	1	2	2	0	1	1	2	2	3	1	0	3
P147	Math	3	0	2	2	2	1	2	1	3	3	0	1	-2	1	2	3	1	2	2	3	2	2	-2	1	2	0	2	1	1
P148	Math	1	0	3	3	3	1	0	3	2	2	1	3	0	3	3	3	3	3	3	3	3	3	1	3	3	3	3	2	2
P148	Math	2	1	2	3	3	1	2	3	3	3	0	1	0	2	0	0	3	3	3	3	3	3	3	3	3	3	3	0	2
P148	Math	3	0	2	2	2	0	3	3	3	2	-1	2	-1	3	3	3	3	3	3	3	2	3	3	-3	3	3	3	2	3
P151	Math	1	1	1	1	0	1	0	1	2	2	2	0	0	1	0	0	1	1	1	1	2	0	0	1	1	1	1	1	1
P151	Math	2	1	1	2	1	2	2	1	2	2	1	1	0	1	0	0	2	2	2	2	1	2	1	3	1	2	2	1	1
P151	Math	3	1	0	2	2	3	0	1	2	2	2	0	0	2	0	0	1	2	2	2	2	1	0	1	1	1	1	1	1
P152	Math	1	0	0	2	2	1	0	0	1	1	0	0	0	1	0	2	1	1	1	2	1	0	0	2	2	0	0	1	0
P152	Math	2	0	2	2	0	1	1	2	2	1	0	0	0	0	0	2	0	0	2	2	2	0	0	2	2	2	2	0	0
P152	Math	3	0	2	3	0	3	3	3	3	3	2	2	0	2	2	0	2	2	3	3	3	2	0	3	2	2	2	2	2
P155	Math	1	3	-3	3	3	3	3	3	3	3	-3	3	0	3	-2	2	-2	0	-1	0	-1	0	0	0	-1	0	1	0	0
P155	Math	2	-1	3	3	0	0	3	3	3	3	3	3	-2	3	1	1	3	3	3	3	1	3	0	3	1	3	3	3	3
P155	Math	3	0	2	3	0	1	3	3	3	3	3	2	-1	3	2	0	1	2	2	3	1	2	0	3	2	3	2	3	2
P156	Math	1	-2	2	-2	-2	2	2	2	2	2	2	0	2	2	2	0	2	2	3	3	1	2	0	3	3	3	2	0	2
P156	Math	2	-3	3	3	3	0	3	3	3	3	-1	3	-3	3	2	3	3	3	3	3	3	2	2	3	2	3	3	1	3
P156	Math	3	0	2	3	3	2	3	3	3	3	-1	2	0	3	3	3	3	3	3	3	2	2	3	3	2	3	1	2	3
P157	Math	1	-1	1	-3	-3	-2	1	0	0	1	-2	-1	-1	1	2	1	1	1	1	1	0	0	1	1	-2	2	0	-1	3
P157	Math	2	-3	1	-2	0	-3	3	-3	3	-3	-3	1	-3	1	3	1	2	-3	-1	0	-2	-3	1	-2	-3	2	-1	-3	0
P157	Math	3	-3	0	-2	0	-3	3	-3	3	-2	0	0	0	0	0	0	-1	-2	-1	0	-3	-1	1	1	-3	3	1	0	0
P159	Mec Eng	1	0	1	-3	-2	0	-2	3	3	2	0	2	2	2	1	1	3	2	2	3	2	1	-1	2	1	2	-1	2	2
P159	Mec Eng	2	-1	0	1	2	1	2	2	-2	1	-1	2	0	2	1	1	3	2	1	2	1	2	0	1	1	3	0	1	1
P159	Mec Eng	3	-1	0	1	2	0	1	2	1	0	0	2	0	2	0	1	3	0	1	1	0	0	0	1	0	1	-1	2	0
P160	Mec Eng	1	-3	2	3	0	2	2	2	2	3	3	1	-1	2	2	1	3	1	2	2	3	1	0	2	2	3	2	1	2

Final AttrakDiff Data (After Removing Careless Respondents)																														
ID	CLASS	ROUND	1	2	3	4	5	6	7	8	9	10	11	12	13	14	15	16	17	18	19	20	21	22	23	24	25	26	27	28
P160	Mec Eng	2	-2	2	1	-1	0	1	1	2	2	2	1	0	1	3	2	2	3	1	2	1	0	0	0	0	1	1	0	1
P160	Mec Eng	3	-2	1	2	-2	-1	1	2	1	2	0	0	0	1	1	2	3	2	1	2	0	0	1	2	0	2	2	1	2
P161	Mec Eng	1	0	2	3	3	1	2	1	3	2	2	2	-2	2	3	2	3	2	3	3	3	2	2	2	3	3	3	3	2
P161	Mec Eng	2	2	2	3	3	3	2	2	3	3	1	3	1	2	3	2	2	3	3	3	2	2	2	2	2	2	3	3	2
P161	Mec Eng	3	2	2	3	3	2	2	2	3	3	2	2	3	2	2	3	2	3	3	3	3	3	2	3	3	2	2	2	2
P162	Mec Eng	1	-1	1	2	-2	0	3	3	2	1	2	3	-1	3	2	-2	2	1	0	2	2	1	-2	2	2	3	3	3	2
P162	Mec Eng	2	-1	3	3	2	1	2	2	2	2	2	2	-3	3	3	2	3	2	2	3	1	1	1	2	3	3	3	3	3
P162	Mec Eng	3	-2	2	2	3	2	3	3	2	2	2	2	-3	3	3	2	3	3	3	2	2	0	1	2	2	3	2	3	2
P163	Mec Eng	1	-1	1	2	3	1	2	2	2	3	2	2	2	2	3	2	2	2	3	3	2	3	2	3	1	1	2	2	2
P163	Mec Eng	2	-1	2	2	2	2	1	2	2	2	0	2	0	3	2	3	2	2	2	2	3	2	2	3	2	2	2	3	2
P163	Mec Eng	3	-1	1	3	3	0	1	2	2	2	2	2	0	3	2	1	2	2	3	2	2	1	2	3	2	2	2	2	2
P164	Mec Eng	1	2	2	2	2	-1	1	2	2	3	0	2	-1	2	1	-1	3	2	3	3	0	2	0	-3	3	1	3	2	2
P164	Mec Eng	2	-1	1	2	-2	-2	1	3	2	2	0	2	-1	1	0	-2	0	2	1	2	-2	1	2	-1	1	2	-1	2	2
P164	Mec Eng	3	-1	-1	2	1	1	1	2	2	2	1	0	1	1	1	0	2	2	2	2	0	1	1	2	1	1	2	2	1
P166	Mec Eng	1	0	1	-1	1	1	0	1	1	1	0	0	-1	1	0	0	0	1	2	1	1	1	0	1	2	1	1	0	0
P166	Mec Eng	2	0	1	2	2	1	1	1	2	2	1	1	-3	2	1	0	1	1	3	2	2	2	0	1	-2	3	1	2	1
P166	Mec Eng	3	0	0	1	1	1	1	-1	3	2	-1	0	0	1	1	0	1	0	1	0	1	1	0	1	1	3	1	0	0
P167	Mec Eng	1	-2	3	3	2	2	2	3	3	3	2	2	1	2	2	2	3	3	3	3	3	1	-1	2	2	2	2	3	2
P167	Mec Eng	2	-2	3	3	3	1	2	2	3	3	2	2	0	2	2	1	3	3	3	3	3	2	-2	2	2	2	2	2	2
P167	Mec Eng	3	-2	2	3	2	1	2	2	2	2	2	3	1	2	3	2	3	2	3	3	2	2	1	2	2	2	2	2	2
P168	Mec Eng	1	0	2	0	0	1	0	-1	-1	2	0	-1	0	0	0	2	1	1	2	1	-1	0	0	1	-1	2	2	-1	1
P168	Mec Eng	2	0	0	1	2	0	2	1	0	1	-1	0	-1	1	0	0	2	2	3	1	0	1	1	1	1	2	1	2	2
P168	Mec Eng	3	0	1	1	0	0	2	2	0	2	1	3	0	2	2	-2	2	2	2	2	0	0	1	0	2	1	2	2	1
P169	Mec Eng	1	0	0	3	0	-2	-1	0	1	1	-2	1	0	0	0	-3	0	0	0	0	0	0	0	0	0	0	0	0	0
P169	Mec Eng	2	2	2	2	0	2	-3	3	3	3	-3	3	-2	2	-2	0	2	2	2	2	1	2	-2	3	2	3	3	3	2
P169	Mec Eng	3	3	3	3	2	3	0	3	3	3	-1	3	0	3	0	3	3	1	3	3	3	0	-2	-2	2	2	3	2	2
P170	Mec Eng	1	0	3	3	3	1	3	3	3	3	1	3	-1	3	3	3	3	3	3	3	3	3	-3	3	3	3	3	3	3
P170	Mec Eng	2	2	2	2	2	0	2	2	3	2	0	2	0	2	2	0	3	2	2	2	2	0	-1	2	2	2	2	2	2

Final AttrakDiff Data (After Removing Careless Respondents)																														
ID	CLASS	ROUND	1	2	3	4	5	6	7	8	9	10	11	12	13	14	15	16	17	18	19	20	21	22	23	24	25	26	27	28
P170	Mec Eng	3	-3	3	3	3	-1	3	3	3	3	2	3	-2	3	3	3	3	3	3	3	3	3	0	3	3	3	3	3	3
P173	Mec Eng	1	-1	2	2	0	2	1	2	2	3	2	1	2	1	2	0	1	1	2	2	2	1	0	1	1	-1	2	2	1
P173	Mec Eng	2	-2	2	2	1	0	1	2	2	2	2	1	0	1	1	1	1	1	1	2	2	1	0	1	1	-1	1	1	-1
P173	Mec Eng	3	0	0	-1	0	0	-1	0	0	-1	-1	0	0	-2	0	0	-1	-1	1	-1	0	-1	1	1	1	-1	-1	0	-1
P174	Mec Eng	1	0	1	2	1	-2	2	0	2	2	0	2	1	2	0	1	3	2	2	3	2	0	2	2	0	2	2	2	1
P174	Mec Eng	2	-2	2	0	0	1	2	2	2	2	2	3	1	2	-2	2	2	2	1	2	1	0	0	1	2	0	2	0	2
P174	Mec Eng	3	0	0	0	0	0	1	2	2	1	1	2	0	2	0	1	2	2	1	2	2	1	0	0	1	0	0	-2	2
P175	Mec Eng	1	-3	0	0	1	-1	3	3	1	-3	0	1	-1	1	1	0	2	-2	3	0	-1	0	-1	3	1	3	2	1	2
P175	Mec Eng	2	-3	2	0	0	0	3	3	3	0	3	1	-3	3	-1	3	3	0	3	3	3	0	-3	2	3	3	1	2	1
P175	Mec Eng	3	-2	3	2	3	0	2	3	3	0	3	2	0	3	0	0	3	3	3	3	3	3	0	3	1	3	2	0	1
P183	Mat Eng	1	-1	1	2	-2	2	2	2	2	2	2	2	2	2	2	2	2	1	2	2	2	2	2	2	1	2	2	2	2
P183	Mat Eng	2	-2	-1	1	1	2	2	1	1	1	1	1	1	1	1	1	1	1	1	2	-1	1	1	1	1	1	2	1	1
P183	Mat Eng	3	-1	-1	-1	1	-2	1	-2	-1	-2	-2	-1	-1	-1	0	-1	-1	-1	1	-1	-2	0	0	0	-2	0	-1	1	0
P189	Mat Eng	1	-1	1	1	0	1	1	0	1	1	0	1	1	0	1	1	1	0	1	1	1	0	0	1	0	1	0	-1	1
P189	Mat Eng	2	-1	2	0	1	0	1	1	1	1	0	1	1	0	1	1	0	0	0	1	0	0	-1	-1	1	1	0	1	0
P189	Mat Eng	3	-1	1	0	1	0	1	1	1	1	1	1	1	0	-1	1	1	0	1	1	-1	0	-1	1	-1	1	1	0	2
P195	Mat Eng	1	2	2	2	2	-1	2	2	3	3	0	3	0	3	3	-1	3	3	3	3	3	3	0	3	3	3	3	3	3
P195	Mat Eng	2	3	3	3	3	1	0	3	3	3	-1	1	0	3	3	-1	3	3	3	3	3	0	3	3	3	3	0	3	3
P195	Mat Eng	3	-3	3	3	3	-2	3	3	0	3	-2	3	-1	3	2	-3	3	3	3	3	0	0	2	1	-1	-1	2	3	-2

5. Mean of Each UX Dimension per Method

In this section, we present the mean of each participant per round and UX dimension. Based on these data, we calculated the overall mean per UX dimension and round to analyze their variation over time and perform statistical analysis.

UX Dimensions from UEQ per Participant and Round							
		ATT	PERSP	EFF	DEP	STIM	NOV
P3	1	3,00	3,00	2,75	2,00	3,00	3,00

UX Dimensions from UEQ per Participant and Round							
		ATT	PERSP	EFF	DEP	STIM	NOV
P3	2	3,00	2,50	3,00	3,00	3,00	3,00
P3	3	1,83	1,50	1,75	1,25	1,75	2,00
P5	1	-0,33	-2,50	0,00	-0,75	-1,00	0,75
P5	2	0,50	-2,50	0,50	-0,50	0,50	0,50
P5	3	-0,50	-2,50	0,00	-1,50	-1,50	0,75
P10	1	2,00	0,25	2,00	0,50	2,50	0,75
P10	2	2,00	1,00	2,00	2,00	3,00	1,50
P10	3	0,83	-0,25	1,00	-0,75	1,00	0,25
P12	1	0,33	-1,50	0,00	-0,50	0,75	1,00
P12	2	1,50	0,50	1,00	1,00	1,50	1,50
P12	3	0,50	0,25	1,00	0,25	0,50	1,25
P15	1	2,00	0,50	0,50	0,75	0,75	-0,75
P15	2	1,00	0,50	1,00	1,00	0,50	1,00
P15	3	0,83	0,75	0,25	-0,25	-0,25	0,50
P17	1	0,67	-0,50	1,75	0,00	0,00	2,00
P17	2	0,00	-0,50	2,00	0,50	0,00	2,00
P17	3	0,17	-0,50	1,25	0,75	0,00	0,50
P19	1	3,00	0,50	3,00	2,25	3,00	1,50
P19	2	3,00	0,00	2,50	2,50	3,00	3,00
P19	3	-0,33	-2,75	2,25	0,75	0,75	3,00
P23	1	1,17	1,75	1,75	1,00	1,25	1,00
P23	2	0,50	0,00	0,00	0,00	0,50	-0,50
P23	3	0,33	0,25	-0,25	-0,25	-0,25	0,00
P24	1	2,17	0,50	2,50	1,75	2,25	2,50
P24	2	-0,50	-2,00	0,00	-1,50	-0,50	1,00
P24	3	1,83	-1,00	2,25	0,25	1,50	1,25
P25	1	1,17	1,50	1,25	1,50	1,25	0,75
P25	2	1,00	1,00	1,00	0,00	0,50	0,50
P25	3	0,50	0,50	0,75	0,75	1,00	0,75

UX Dimensions from UEQ per Participant and Round							
		ATT	PERSP	EFF	DEP	STIM	NOV
P28	1	2,17	0,50	1,25	1,25	1,75	2,50
P28	2	1,00	-0,50	1,00	0,50	0,00	1,00
P28	3	1,50	1,00	1,25	1,50	0,50	2,00
P30	1	2,50	-0,25	2,00	1,75	1,25	-0,25
P30	2	3,00	-2,00	3,00	3,00	2,00	2,00
P30	3	-0,17	-0,25	0,50	1,50	0,25	1,25
P40	1	2,00	0,75	2,00	1,25	1,25	0,75
P40	2	3,00	3,00	3,00	3,00	3,00	3,00
P40	3	2,17	2,00	2,00	2,00	1,50	2,00
P44	1	0,50	0,25	0,00	1,00	-0,50	0,00
P44	2	-2,00	-1,50	-1,00	-1,50	-1,00	0,00
P44	3	-0,33	-0,25	-0,25	-1,25	-0,75	0,25
P47	1	2,00	1,25	1,75	1,75	2,00	2,00
P47	2	2,00	2,00	2,00	2,00	1,50	2,00
P47	3	1,83	0,75	1,75	1,50	1,00	1,50
P54	1	2,17	1,75	2,00	1,50	1,75	2,25
P54	2	2,00	1,50	2,00	1,50	1,50	2,00
P54	3	2,17	1,25	2,25	1,25	1,50	2,25
P55	1	0,67	0,25	0,50	0,50	0,50	0,25
P55	2	0,00	0,00	0,00	0,00	0,50	0,00
P55	3	1,17	0,25	0,25	0,00	1,25	1,50
P56	1	2,33	0,75	2,50	1,75	2,00	3,00
P56	2	2,00	0,50	2,50	2,00	2,00	3,00
P56	3	2,50	2,25	2,75	1,75	3,00	3,00
P57	1	1,00	0,25	0,50	0,25	0,75	1,00
P57	2	2,00	1,50	2,00	2,00	2,50	1,50
P57	3	2,00	1,50	1,75	1,25	2,00	0,75
P60	1	1,67	0,00	1,00	0,25	0,75	0,50
P60	2	3,00	-2,50	2,50	0,00	2,00	2,50

UX Dimensions from UEQ per Participant and Round							
		ATT	PERSP	EFF	DEP	STIM	NOV
P60	3	1,33	-2,50	-1,00	-0,75	0,50	1,00
P61	1	1,50	1,75	2,00	1,50	1,75	0,75
P61	2	1,00	1,00	1,00	0,50	1,00	1,00
P61	3	1,17	1,50	1,00	1,00	0,75	1,50
P64	1	2,83	2,00	1,25	1,50	2,75	2,00
P64	2	2,00	2,00	2,50	2,50	1,50	2,00
P64	3	1,33	0,75	1,25	1,25	1,00	1,50
P68	1	1,50	1,00	1,00	0,25	0,25	0,25
P68	2	0,00	-1,00	1,00	-0,50	-1,00	0,00
P68	3	0,33	0,75	0,75	-0,50	0,25	0,25
P72	1	0,83	0,75	0,50	0,00	-0,50	1,25
P72	2	1,00	0,00	1,50	1,00	1,00	1,50
P72	3	-0,17	-1,00	0,25	-0,50	1,00	0,50
P74	1	2,50	2,00	2,00	2,00	2,75	2,25
P74	2	2,00	2,00	2,00	1,50	1,50	2,00
P74	3	1,83	1,25	1,50	1,50	2,00	2,50
P75	1	1,33	1,25	1,00	0,50	1,25	0,50
P75	2	2,00	1,50	2,00	1,00	1,50	1,00
P75	3	1,17	1,25	1,75	1,00	1,25	1,00
P77	1	2,83	2,50	2,50	1,50	2,00	2,75
P77	2	3,00	2,50	2,00	2,50	3,00	2,00
P77	3	2,17	2,00	2,50	1,50	1,75	2,50
P78	1	2,50	1,25	2,00	1,50	2,50	2,75
P78	2	3,00	2,00	2,50	3,00	2,50	3,00
P78	3	2,00	2,25	2,50	2,00	3,00	1,75
P80	1	0,17	0,50	0,50	-0,50	0,75	1,00
P80	2	0,00	-0,50	1,00	0,00	0,00	0,50
P80	3	2,17	1,00	2,75	1,75	1,25	1,00
P85	1	2,33	0,00	2,00	1,50	2,50	2,75

UX Dimensions from UEQ per Participant and Round							
		ATT	PERSP	EFF	DEP	STIM	NOV
P85	2	2,00	-1,00	2,00	1,00	2,50	3,00
P85	3	0,67	-1,25	1,25	0,75	1,00	2,50
P94	1	0,00	-1,50	0,00	0,50	-0,25	-0,50
P94	2	0,00	1,00	1,00	2,00	0,00	0,00
P94	3	-0,33	-0,25	-0,50	0,25	-1,75	-0,50
P99	1	2,50	2,25	2,75	2,00	2,50	3,00
P99	2	2,50	2,50	2,75	2,00	1,25	2,25
P99	3	2,50	3,00	2,00	2,25	2,25	3,00
P101	1	0,83	0,75	1,00	0,50	0,50	0,00
P101	2	1,00	0,50	0,50	1,25	1,00	0,75
P101	3	0,67	0,50	0,50	0,50	0,75	0,00
P104	1	1,83	0,50	2,00	0,50	1,50	1,00
P104	2	1,17	0,50	1,75	0,75	0,75	0,50
P104	3	0,67	0,50	0,25	0,75	0,50	0,50

UX Dimensions from AttrackDiff per Participant and Round					
		PQ	HQI	HQS	ATT
P105	1	0,14	0,57	1,00	1,14
P105	2	-0,29	0,00	-0,14	0,00
P105	3	0,29	0,57	0,57	1,14
P111	1	0,29	1,14	1,57	1,71
P111	2	0,43	1,14	1,43	2,14
P111	3	0,71	1,14	1,29	2,00
P121	1	1,29	1,86	1,57	2,14
P121	2	0,57	0,86	1,00	0,86
P121	3	0,86	1,57	1,57	1,71
P122	1	0,43	1,29	0,43	1,71
P122	2	0,43	0,57	0,57	0,29
P122	3	0,29	0,29	-0,14	0,43
P124	1	0,86	1,14	1,57	1,71
P124	2	-0,86	0,14	0,43	0,43
P124	3	0,29	1,00	-0,43	0,00
P126	1	1,14	1,14	1,00	1,29
P126	2	0,29	0,43	1,00	-0,71
P126	3	-0,14	0,71	0,29	-0,14
P127	1	0,00	1,29	1,29	1,14
P127	2	0,71	1,86	1,57	1,43
P127	3	0,43	0,86	0,71	1,00
P132	1	1,86	2,43	2,29	2,57
P132	2	3,00	2,86	0,43	3,00
P132	3	2,71	3,00	2,57	2,86
P137	1	0,86	1,71	2,29	2,14
P137	2	1,57	2,71	2,57	2,71

UX Dimensions from AttrackDiff per Participant and Round					
		PQ	HQI	HQS	ATT
P137	3	0,29	2,57	2,29	2,57
P138	1	0,71	1,43	0,57	1,29
P138	2	0,71	1,00	0,00	0,86
P138	3	1,43	1,29	1,57	1,43
P147	1	0,71	1,29	1,00	1,57
P147	2	1,00	0,29	1,71	1,00
P147	3	0,71	1,71	0,86	2,14
P148	1	1,29	2,57	2,57	2,86
P148	2	1,43	1,43	2,57	3,00
P148	3	0,86	2,71	1,86	2,71
P151	1	1,29	0,43	0,71	1,00
P151	2	1,14	1,00	1,57	1,86
P151	3	1,57	0,43	1,14	1,57
P152	1	0,43	0,57	1,14	0,86
P152	2	0,71	0,71	1,14	1,29
P152	3	1,86	1,86	1,71	2,57
P155	1	0,71	0,57	0,14	1,43
P155	2	1,00	2,43	1,86	3,00
P155	3	1,29	1,86	1,86	2,57
P156	1	1,29	1,43	1,43	1,57
P156	2	0,29	2,86	2,43	2,86
P156	3	1,29	2,71	2,71	2,57
P157	1	-0,43	0,86	-0,14	0,00
P157	2	-1,57	1,71	-0,86	-2,14
P157	3	-0,86	0,29	0,14	-1,29
P159	1	1,29	1,14	0,86	1,00
P159	2	-0,14	1,57	1,29	1,43

UX Dimensions from AttrackDiff per Participant and Round					
		PQ	HQI	HQS	ATT
P159	3	0,00	1,29	1,00	0,43
P160	1	1,14	1,86	1,43	2,00
P160	2	0,57	1,71	0,14	1,43
P160	3	0,00	1,29	0,71	1,71
P161	1	1,29	2,29	2,71	2,29
P161	2	2,00	2,29	2,43	2,71
P161	3	2,43	2,14	2,57	2,71
P162	1	0,86	1,71	0,86	1,86
P162	2	0,71	2,57	2,29	2,29
P162	3	0,71	2,57	2,43	2,00
P163	1	1,43	2,00	2,14	2,43
P163	2	1,14	2,14	2,29	2,00
P163	3	1,00	1,71	2,43	2,00
P164	1	0,57	1,43	1,14	2,43
P164	2	-0,29	0,43	0,71	1,57
P164	3	0,71	0,57	1,43	1,86
P166	1	0,29	0,29	1,00	0,71
P166	2	0,57	1,00	1,29	1,57
P166	3	0,57	0,57	1,00	0,57
P167	1	1,57	2,29	1,86	2,57
P167	2	1,29	2,14	1,71	2,57
P167	3	1,14	2,43	2,00	2,29
P168	1	0,00	0,57	0,43	0,71
P168	2	0,00	0,71	1,71	1,14
P168	3	0,29	1,43	1,14	1,57
P169	1	-0,43	-0,43	0,00	0,57
P169	2	0,71	0,57	1,57	2,43

UX Dimensions from AttrackDiff per Participant and Round					
		PQ	HQI	HQS	ATT
P169	3	1,86	2,14	1,00	2,29
P170	1	1,43	3,00	2,14	3,00
P170	2	1,29	1,86	1,57	1,71
P170	3	0,71	3,00	2,57	3,00
P173	1	1,43	1,14	0,71	1,86
P173	2	0,43	1,14	0,57	1,57
P173	3	-0,29	-0,57	0,43	-0,86
P174	1	0,57	1,57	1,57	1,57
P174	2	1,00	1,57	0,57	1,43
P174	3	1,00	1,14	0,00	1,14
P175	1	-0,43	1,14	1,57	0,00
P175	2	0,57	2,00	1,43	1,00
P175	3	1,14	1,86	1,86	2,29
P183	1	1,57	1,86	1,29	1,86
P183	2	0,43	0,86	1,00	1,29
P183	3	-1,29	-0,57	0,14	-1,14
P189	1	0,57	0,86	0,29	0,43
P189	2	0,14	0,86	0,29	0,43
P189	3	0,43	0,57	0,29	0,57
P195	1	1,43	2,14	2,43	2,71
P195	2	1,71	1,71	3,00	2,14
P195	3	-1,43	2,00	1,43	2,43

6. Sentence Completion Data

This section presents the data from Sentence Completion per group.

Sentence Completion Results from UEQ Group						
ID	ROUND	CLASS	WORST THING	CODE	MOST PLEASES	CODE

Sentence Completion Results from UEQ Group						
ID	ROUND	CLASS	WORST THING	CODE	MOST PLEASES	CODE
P3	1	Phys	notations	Content related	fun in learning	Hedonic
P3	3	Phys	some hints do not help	Learning support	the way how it is used	Use
P5	1	Phys	kinda incomprehensible	Perspicuity	the gamification	Gamification
P5	3	Phys	Everything	Not identified	the game	Gamification
P10	1	Math	to understand	Perspicuity	to learn	Learnability
P10	3	Math				
P12	1	Mec Eng	the confusion	Perspicuity	innovation	Innovation
P12	3	Mec Eng	mechanisms	Not identified	practicality	Practicality
P15	1	Math	Code errors	Identify errors	exercises resolution	Practicing
P15	3	Math				
P17	1	Mat Eng	confusing	Perspicuity	modernity	Innovation
P17	3	Mat Eng				
P19	1	Mat Eng	when it hangs	Performance	the easy way it teaches you how to program	Learning support
P19	3	Mat Eng	when it hangs	Performance	the lab	Not identified
P23	1	Mat Eng	to be slow	Performance	the game	Gamification
P23	3	Mat Eng	slow	Performance	the lab	Not identified
P24	1	Mat Eng	Structures are much complex	Content related	the instructions	Learning support
P24	3	Mat Eng	the non-facilitating platform	Learning support	the organization	Interface
P25	1	Mat Eng	the bugs that occur after the completion of an exercise.	Bug	the dynamic of the activities	Content related
P25	3	Mat Eng				
P28	1	Mat Eng	the time to check the program	Bug	efficiency	Performance
P28	3	Mat Eng	during the correction, sometimes it shows that the answer is wrong, even when it is correct	Bug	design	Interface
P30	1	Mat Eng	difficult	Easiness	it is modern	Innovation
P30	3	Mat Eng	Difficult	Easiness	Nothing	Not identified
P40	1	Phys	The assignments	Content related	The game	Gamification
P40	3	Phys	lockup	Performance	the game	Gamification

Sentence Completion Results from UEQ Group						
ID	ROUND	CLASS	WORST THING	CODE	MOST PLEASES	CODE
P44	1	Phys	too simple	Functionality	easiness to do the exercises	Easiness
P44	3	Phys	give error when results are correct	Bug	friendly interface	Interface
P47	1	Phys	that the IDEs could be more complete	Functionality	the gamification and all the story behing the game motivates me even more to keep performing the activities	Gamification
P47	3	Phys	that sometimes unfortunately becomes unmotivating and boring	Hedonic	that it is a way to motivate us to program even though there are still many points to be improved.	Gamification
P54	1	Math	Nothing	Not identified	Criativity	Innovation
P54	3	Math				
P55	1	Math	nothing	Not identified	the gamification	Gamification
P55	3	Math	that it hangs during the exam	Performance	gamification	Gamification
P56	1	Math	try to learn	Learnability	being modern	Innovation
P56	3	Math	a little difficult to learn	Learnability	it being facilitator	Learning support
P57	1	Math	some things	Not identified	it all	Not identified
P57	3	Math				
P60	1	Math	nothing	Not identified	the easiness	Easiness
P60	3	Math				
P61	1	Math	it does not have a chat in the game	Functionality	THE GAME	Gamification
P61	3	Math	it hangs during the exam	Performance	the easiness to code	Easiness
P64	1	Mec Eng	the game	Gamification	the easiness to learn the subjects	Learning support
P64	3	Mec Eng	the gamification	Gamification	be able to use it anywhere	Flexibility
P68	1	Mec Eng	slowness	Performance	hints	Learning support
P68	3	Mec Eng				
P72	1	Mec Eng	badly formulated questions	Perspicuity	the speed and efficiency in explaining our mistakes	Learning support
P72	3	Mec Eng	the correction	Bug	the hints and input examples	Learning support
P74	1	Mec Eng	it could have more challenges	Content related	the organized interface	Interface

Sentence Completion Results from UEQ Group						
**ID**	**ROUND**	**CLASS**	**WORST THING**	**CODE**	**MOST PLEASES**	**CODE**
P74	3	Mec Eng			The challenges	Content related
P75	1	Mec Eng	the gamification platform does not work outside the lab, on my pc for example	Compatiblity	to be able to program without the need for an instructor	Autonomy
P75	3	Mec Eng	sometimes it has some problems when you submit the code, it says that is wrong, and soon after, without changing anything and submitting again, it says that is right.	Bug	it is dynamic and easy	Easiness
P77	1	Mec Eng	bugs in the game	Bug	be able to train programming simply and easily	Practicing
P77	3	Mec Eng	some bugs	Bug	the possibility to study on my own easily	Autonomy
P78	1	Mec Eng	it doesn't have a mobile app of their own	Portability	the organization and practicality	Practicality
P78	3	Mec Eng	support	Not identified	innovation	Innovation
P80	1	Mec Eng	various codes	Content related	layout	Interface
P80	3	Mec Eng	when I make a mistake in a question during the exam	Personal	to get the answer right in the exam	Personal
P85	1	Mat Eng	to have to create codes	Practicing	it is easy to use	Easiness
P85	3	Mat Eng	after programming not printing, not knowing where the error is	Identify errors	the way it uses gamification to not get bored	Gamification
P94	1	Mec Eng	to program	Practicing	to program	Practicing
P94	3	Mec Eng	the codes	Content related	the interface	Interface
P99	1	Mat Eng	i don't know	Not identified	site clarity	Perspicuity
P99	3	Mat Eng	i don't know	Not identified	I don't know either	
P101	1	Mec Eng	still don't know	Not identified	the interface	Interface
P101	3	Mec Eng	wrong hints	Learning support	right hints	Learning support
P104	1	Mat Eng	still don't know what to say	Not identified	still don't know	
P104	3	Mat Eng	some bugs (delay)	Bug	to be able to program	Practicing

Sentence Completion Results from AttrakDiff Group						
ID	ROUND	CLASS	WORST THING	CODE	MOST PLEASES	CODE
P105	1	Phys	.		.	
P105	3	Phys	nothing	Not identified	creative	Innovation
P111	1	Math	to be so much perfect	Not identified	design	Interface
P111	3	Math	when I write the code correctly and it says it is wrong	Bug	the design is attractive	Interface
P121	1	Mat Eng	takes time to load some information	Performance	practicality	Practicality
P121	3	Mat Eng	When it takes time to load	Performance	practicality	Practicality
P122	1	Mat Eng	the fact that gamification doesn't work properly sometimes	Bug	the challenge of trying to create a program.	Practicing
P122	3	Mat Eng				
P124	1	Mat Eng	greater freedom in algorithms	Restrictive	easiness	Easiness
P124	3	Mat Eng				
P126	1	Mat Eng	bug in the game	Bug	to move through the steps	Practicing
P126	3	Mat Eng	it does not accept answer of same value	Bug		
P127	1	Mat Eng	little explanatory	Perspicuity	the easiness to access	Access
P127	3	Mat Eng	the gradings	Course related	when it ends	Not identified
P132	1	Phys	the bugs in the RPG game when performing the activities proposed	Bug	the way the activities are handled together with a rpg game	Gamification
P132	3	Phys	The bugs on google chrome	Bug	Didactic	Learning support
P137	1	Phys	sometimes I can't access exercises that should be available outside of class hours	Unavailability	The design is modern, likable, and easy to use	Interface Easiness
P137	3	Phys	the platform not being available as an app for android or ios	Portability	its presentation and practicality	Practicality Interface
P138	1	Phys	The lack of detailed information	Perspicuity	the automatic correction	Functionality
P138	3	Phys	it hangs	Performance	it is simple	Practicality
P147	1	Math	The bugs on google chrome	Bug	the integration of challenges into the game	Gamification
P147	3	Math	when it loops forever	Bug	everything	Not identified

Sentence Completion Results from AttrakDiff Group						
ID	ROUND	CLASS	WORST THING	CODE	MOST PLEASES	CODE
P148	1	Math	i don't know	Not identified	the fun	Hedonic
P148	3	Math	sometimes it bug	Bug	practicality	Practicality
P151	1	Math	lack of previous contact	Familiarity	learning	Learning
P151	3	Math				
P152	1	Math	I can't opine	Not identified	the simplicity	Practicality
P152	3	Math	Lockings	Performance	Interface	Interface
P155	1	Math	nothing		everything	Not identified
P155	3	Math	questions not in the study content	Content related	versatility	Flexibility
P156	1	Math	sometimes it hangs	Performance	practicality	Practicality
P156	3	Math	the lack of clarity on where the errors are located	Identify errors	the practicality	Practicality
P157	1	Math	The language	Content related	to test the codes	Practicing
P157	3	Math				
P159	1	Mec Eng	when the characters in the game locks you	Bug	the visual	Interface
P159	3	Mec Eng				
P160	1	Mec Eng	hangs too much	Performance	the easiness	Easiness
P160	3	Mec Eng				
P161	1	Mec Eng	explanation	Perspicuity	the learning method	Learning support
P161	3	Mec Eng	ambiguity	Perspicuity	its layout	Interface
P162	1	Mec Eng	i don't know		the challenges	Content related
P162	3	Mec Eng				
P163	1	Mec Eng	the gamification bugs	Bug	its friendly and intuitive interface	Interface
P163	3	Mec Eng	the gamification bugs since i stopped playing due to the amount of bugs	Bug	i can be inventive in my codes without any restrictions	Learning support
P164	1	Mec Eng	type of questions	Content related	practicality	Practicality
P164	3	Mec Eng				
P166	1	Mec Eng	the site has errors eventually	Bug	the easiness to practice programming	Easiness
P166	3	Mec Eng				
P167	1	Mec Eng	new	Innovative	platform	The system

Sentence Completion Results from AttrakDiff Group						
ID	ROUND	CLASS	WORST THING	CODE	MOST PLEASES	CODE
P167	3	Mec Eng				
P168	1	Mec Eng	graphics	Graphics	playability	Gamification
P168	3	Mec Eng				
P169	1	Mec Eng	To program	Practicing	Jad	Not identified
P169	3	Mec Eng	when it accepts my code then refuse	Bug	finish my lab	Not identified
P170	1	Mec Eng	instructions on how to create complex code	Learning support	the operational system itself	The system
P170	3	Mec Eng				
P173	1	Mec Eng	gamification bugs	Bug	practicality	Practicality
P173	3	Mec Eng				
P174	1	Mec Eng	for not having more tabs in the IDE to build various algorithms	Functionality	the interactivity	Interactivity
P174	3	Mec Eng				
P175	1	Mec Eng	the bugs	Bug	the creativity	Innovation
P175	3	Mec Eng	The bugs	Bug	The hints help a lot	Learning support
P183	1	Mat Eng	unattractive	Attractiveness	is the practice	Practicing
P183	3	Mat Eng	submit the programs	Bug	the beauty	Interface
P189	1	Mat Eng	i don't know		is practical	Practicality
P189	3	Mat Eng	manipulator	Obstruction	motivational	Hedonic
P195	1	Mat Eng	challenging	Stimulation	attractive	Attractiveness
P195	3	Mat Eng				
